# ﻿Monograph of *Ceratozamia* (Zamiaceae, Cycadales): an endangered genus

**DOI:** 10.3897/phytokeys.208.80382

**Published:** 2022-09-21

**Authors:** Lilí Martínez-Domínguez, Fernando Nicolalde-Morejón, Francisco Vergara-Silva, Dennis Wm. Stevenson

**Affiliations:** 1 Posgrado en Ciencias Biológicas, Instituto de Biología, Universidad Nacional Autónoma de México, 3er. Circuito Exterior, Ciudad Universitaria, 04510, Coyoacán, CDMX, Mexico; 2 Laboratorio de Teoría Evolutiva e Historia de la Ciencia, Instituto de Biología, Universidad Nacional Autónoma de México, 3er. Circuito Exterior, Ciudad Universitaria, 04510, Coyoacán, CDMX. Mexico; 3 Laboratorio de Taxonomía Integrativa, Instituto de Investigaciones Biológicas, Universidad Veracruzana, Xalapa, 91190, Veracruz. Mexico; 4 The New York Botanical Garden, Bronx, New York, 10458-5120, USA

**Keywords:** Circumscription, cryptic species, cycads, lectotypification, Mesoamerica, Mexico

## Abstract

*Ceratozamia* (Zamiaceae, Cycadales), is a member one of the most endangered seed plant groups. Species of *Ceratozamia* grow from near sea level up to 2,100 meters in Mexico and Mesoamerica. We present a modern taxonomic treatment of *Ceratozamia*, based on fieldwork combined with detailed study of herbarium specimens in and from Mexico and Central America. This new revision is based on incorporation of morphological, molecular and biogeographic data that have been previously published in circumscriptions of species complexes by our group. Detailed morphological descriptions of the 36 species of *Ceratozamia* are provided and relevant characters for the genus are discussed and described. A majority are endemic to Mexico and are concentrated at high elevations in mountainous areas. Synonymies, lectotypifications, etymologies, taxonomic notes, distribution maps, illustrations and detailed species-level comparisons are included, as well as a dichotomous key for identification of all species. Data on distributional ranges and habitats of all species are summarized. *Ceratozamiaosbornei* D.W.Stev., Mart.-Domínguez & Nic.-Mor., **sp. nov.** is described from evergreen tropical forests of Belize and we highlight new populations and distributional ranges for *C.subroseophylla* Mart.-Domínguez & Nic.-Mor. and *C.vovidesii* Pérez-Farr. & Iglesias in the Mesoamerican region.

## ﻿Introduction

*Ceratozamia* Brongn. (Zamiaceae, Cycadales) is characterized by being dioecious with both megasporophylls and microsporophylls having two distinct apical horns. The 36 species of *Ceratozamia* occur in the tropical region of Mega-Mexico II *sensu*Rzedowski (1991), which was established using floristic criteria and includes Mexico, Guatemala, Belize and northern Nicaragua. This genus inhabits in all these countries except Nicaragua and the greatest diversity and endemism in *Ceratozamia* are concentrated in the Sierra Madre Oriental and Southwest regions of Mexico (Nicolalde-Morejón et al. 2014; Martínez-Domínguez et al. 2018a). Species described as *Ceratozamia* from outside this region are properly recognized as other cycad genera (see Excluded names). The genus primarily occurs in oak forest, cloud forest and tropical rain forest on karstic rocks, at an elevational range of 19–2,050 m. *Ceratozamia* is morphologically distinguished from other genera of Zamiaceae by its two parallel adaxial canals along the rachis, entire leaflet margins, and two horns on the distal end of sporophylls in ovulate and pollen strobili.

*Ceratozamia* is monophyletic with a fossil record dating to the lower Oligocene (Kvaček 2014). Recent phylogenetic analyses of molecular data have supported *Stangeria* (Kunze) Baill. as sister to *Ceratozamia* (Nagalingum et al. 2011) while others have placed *Stangeria* as sister to *Microcycas* (Miq.) A.DC. + *Zamia* L. (Salas-Leiva et al. 2013; Condamine et al. 2015; Liu et al. 2022). The phylogenetic relationships within *Ceratozamia* have been explored using different molecular data sets. González and Vovides (2012) sampled half of the known species and used three genes (*nrITS*, *trn*L-F, *D-SCAR*). More recently, a phylogenetic analysis of this genus that included 28 species and a molecular data set composed of six genes (*nrITS*, rpoC1, matK, rbcL, *psbK/I*, *atpF/H*) produced a topology resolved with low support values (Medina-Villarreal et al. 2019); in turn, Martínez-Domínguez et al. (2020) sampled 32 species and only two genes (*nrITS* and matK) but produced a poorly resolved topology. Studies of diversification ages of Cycadales using phylogenomic analyses have included between 70 and 83% of currently accepted *Ceratozamia* species, which recovered better node support (Nagalingum et al. 2011; Condamine et al. 2015; Liu et al. 2022). As a result, species relationships within *Ceratozamia* are becoming clearer and more work will be needed with continued exploration that will undoubtedly lead to new discoveries.

Since the description of the genus by Brongniart (1846), there has been an explosion of interest in this group. Miquel (1848) described five new species using only vegetative characters. *Ceratozamia* poses a taxonomic challenge, however, due to its significant vegetative morphological variation, which was a fact realized by Miquel (1868, 1869a) when he lumped all of his previously described species into *C.mexicana* Brongn. These early taxonomic treatments varied greatly in the number of species recognized within the genus with contrasting findings in each treatment (Regel 1857a, b; Miquel 1861, 1868, 1869a, b; Regel 1876a, b; Thiselton-Dyer 1884; Schuster 1932). Particularly, the ‘Miquel taxonomic names’ have undergone a series of rearrangements in synonymy and recognition of infraspecific categories. Many of these names were clarified by Stevenson and Sabato (1986) when they focused on the typification of all described species in the genus at that time, but did not include a list of invalid names. More recently, a regional taxonomic review focused on the Mexican Sierra Madre Oriental provided new observations that have allowed detailed reassessment and designation of nomenclatural types for some of these names (Martínez-Domínguez et al. 2018a).

Explorations in Mexico during the past three decades have uncovered new species of *Ceratozamia*, thereby, rapidly increasing its known diversity. Nevertheless, the taxonomy of *Ceratozamia* has been mainly characterized by rearrangements of infrageneric taxa, treatments in floristic works and species-level treatments of species complexes (Vovides et al. 1983; Stevenson et al. 1986; Pérez-Farrera et al. 1999; Avendaño et al. 2003; Pérez-Farrera et al. 2009; Vovides et al. 2012; Martínez-Domínguez et al. 2016, 2017c). Recent taxonomic efforts based on analyses of quantitative and qualitative morphological variation, phenology as well as DNA sequences of nuclear, plastid and mitochondrial genomes have produced new insights on the circumscription of species complexes and the most comprehensive taxonomic reviews of *Ceratozamia* to date (Martínez-Domínguez et al. 2016, 2017a, b, 2018a, 2022a). In addition, work focusing on the morphometric variation of vegetative characters and anatomical features have contributed to the clarification of some species (Pérez-Farrera et al. 2014; Vovides et al. 2016; Medina-Villarreal et al. 2019; Gutiérrez-Ortega et al. 2021). These studies have greatly enhanced our understanding of morphological variation, particularly in vegetative characters. Recently, genomic data from 100 nuclear single-copy genes have provided evidence for future directions in the study of the ontogeny of reproductive structures and associated evolutionary processes (Liu et al. 2022).

Currently, quantitative vegetative morphological characters are considered as the most taxonomically informative characters within *Ceratozamia* (Vovides et al. 2004; Pérez-Farrera et al. 2009). Because these types of characters exhibit high intra- and interspecific variation, renewed morphological exploration into reproductive structures has contributed additional information. This is particularly the case for ovulate strobilus characters, such as the angle between the horns of the megasporophylls, the strobilus apex and the shape of the distal face of megasporophylls (Martínez-Domínguez et al. 2018a). Additional characters are also present in pollen strobili such as the shape of the microsporophylls, the size of the horns, and shape of the fertile and infertile part. However, the use of reproductive characters has been limited by the difficulty of collecting reproductive structures in the field and the lack of these in herbaria. Also, usually ovulate and/or pollen strobili are fragmented as a result of incorrect processing of these structures and damage during handling.

The reproductive phenology of *Ceratozamia* is annual (Stevenson 1981). To date, our knowledge of the phenological phases for ovulate and pollen strobili indicates four phases in each (Martínez-Domínguez et al. 2022a). In both pollen and ovulate strobili the cycle starts with emergence, which is characterized by emergence from among the cataphylls. In pollen strobili, the sequence goes from a closed strobilus, followed by an open strobilus with dehiscence of the sporangia and, finally, senescence. In ovulate strobili, opening cracks develop between sporophylls (indicating receptivity), then a late ovulate strobilus with seeds if pollinated and, finally, disintegration (Martínez-Domínguez et al. 2018b). The receptivity and open pollen phases are critical for pollination and subsequent fertilization of ovules because this genus is pollinated by “pleasing fungus beetles” of family Erotylidae (Norstog and Nicholls 1997).

In this paper, as part of our comprehensive systematic review of the genus, we describe in detail ovulate and pollen strobili, including internal structures and their character states. This was possible by broad sampling in wild populations. We also examine overlooked morphological characters, conduct a nomenclatural review of species, and offer a comprehensive identification key for the genus. Here, we synthesize biogeographic information to account for their distributional range. By default, *Ceratozamia* and all of its species are listed and covered by CITES Appendix 1, but only 21 of the 36 species have had asessments (Calonje et al. 2013–2022; IUCN 2021). Those data are included in species treatments when available. Thus, this monograph is particularly timely and valuable in this era of habitat destruction and illegal collecting. Having a complete taxonomic treatment will allow for appropriate identifications that will lead to informed conservation assessments and actions (Stevenson et al. 2003; Rutherford et al. 2013; Martínez-Domínguez et al. 2021).

## ﻿Materials and methods

The species circumscriptions for *Ceratozamia* presented here are based on extensive fieldwork, as well as review of specimens from a substantial number of herbaria, including BIGU, BM, BRH, CAS, CH, CHAPA, CHIP, CIB, EAP, ENCB, F, FCME, FTG, GH, HEM, HNT, IBUG, IEB, K, L, LE, LSU, MICH, MEXU, MO, NAP, NY, P, SERO, SLPM, TEFH, U, US, XAL, XALU, UAT, USCG (acronyms according to Thiers 2022), either by examining the specimens physically or electronically through the Global Plants web portal (http://plants.jstor.org/). Because most cycad specimens are sterile, we have taken the opportunity to record the presence of cones when they are present in a collection. We also examined the protologues of all species described and exhaustively searched for type specimens in the registered herbaria. It should be noted, however, that the type specimens for *C.chimalapensis* Pérez-Farr. & Vovides, *C.dominguezii* Pérez-Farr. & Gut.Ortega, *C.sancheziae* Pérez-Farr., Gut.Ortega & Vovides, and *C.zoquorum* Pérez-Farr., Vovides & Iglesias could not be found. Decisions on lectotypes were taken only when we confirmed that there was no evidence of the holotype’s existence at the herbaria cited in the protologue. The specimens collected by us were under scientific collection permit SGPA/DGVS/5506 from SEMARNAT (Mexico) and MP-0209-2021 from ICF (Honduras). The geographical coordinates of each specimen were compiled in a database in ArcMap 10.2 (Esri, Redlands, USA) to determine the distribution of *Ceratozamia* using the biogeographic regionalization of the Neotropical region scheme of Morrone et al. (2022). Because species are all CITES listed, the specimens examined only indicate Municipality and elevation.

**Criteria to delimit species.** The taxonomic treatment presented here is partly based on our previously published work on species complexes, considered from an integrative taxonomy perspective for corroboration or refutation of taxonomic hypotheses and employing the “taxonomic circle” *sensu*DeSalle et al. (2005), in which multiple data sources are analyzed independently for hypotheses testing (Martínez-Domínguez et al. 2016; 2017a, b, 2018a, 2020, 2022a, b). For taxa we have not treated previously, species were recognized by a unique combination of morphological characters evaluated at the population level, considering their biogeographic distribution patterns as an additional source of evidence. We have recognized neither subspecies nor varieties, but have rather described the variability when it was present.

**Figure 1.**
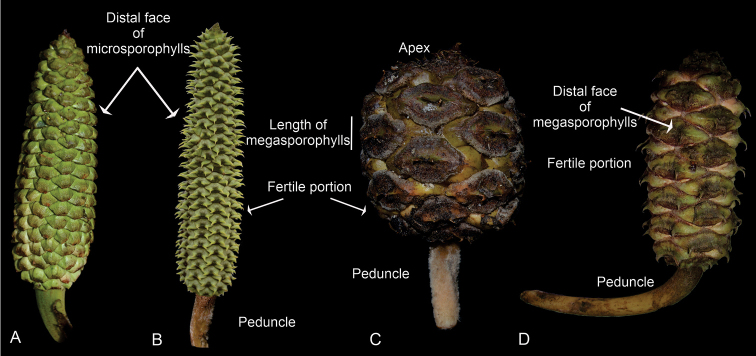
Characters evaluated in reproductive structures **A** pollen strobilus of *Ceratozamiasabatoi***B** pollen strobilus of *C.santillanii***C** ovulate strobilus of *C.aurantiaca***D** ovulate strobilus of *C.zoquorum*.

**Figure 2.**
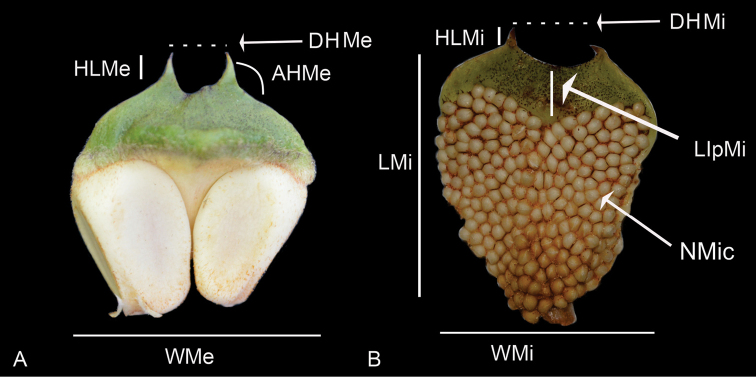
Characters evaluated in sporophylls **A** megasporophylls of *Ceratozamiadelucana***B** microsporophylls of *C.delucana*. HLMe = Horn length of megasporophylls; DHMe = Distance between horn of megasporophylls; AHMe = Angle between horns of megasporophylls; WMe = Width of megasporophylls; LMi = Length of microsporophylls; WMi = Width of microsporophylls; NMic = Number of microsporangia; LlpMi = Length infertile portion of microsporophylls; DHMi = Distance between horn of microsporophylls; HLMi = Horn length of microsporophylls.

**Specialized characters and terminology.** The morphological terminology used in the descriptions was standardized according to Moreno (1984) and Harris and Harris (2000). Most *Ceratozamia* reproductive structures and characters have been recently described (Martínez-Domínguez et al. 2020). We evaluated 20 qualitative and 23 quantitative reproductive characters in pollen and ovulate strobili (Figs 1, 2; Table 1; Suppl. material 1). Generally, the terminology for the orientation of ovulate strobilus is given using the terms “decumbent” or “erect”. However, the fertile portion of a strobilus could be inclined at an angle of 180° or prostrate, but not curved. Usually, this condition is derived from weight at maturity. Only the peduncle is curved or erect; the definition for decumbent is reclined with tip ascending, but the peduncle in this genus is not ascending. Here, we have used “pendulous”, which is a more appropriate term and is defined as drooping downward, rather than “decumbent”, as often appears in previous literature.

**Table 1.** Qualitative and quantitative morphological characters evaluated in *Ceratozamia* reproductive structures.

Character	Character state
Pollen strobilus position	(0) erect; (1) pendulous
Pollen strobilus shape	(0) angulate; (1) cylindrical
Pollen strobilus color	(0) greenish yellow; (1) greenish brown (2) reddish brown; (3) yellowish brown
Distal face of microsporophylls	(0) non-recurved; (1) recurved
Microsporophylls shape	(0) discoid; (1) obconic; (2) elliptic
Microsporophylls horns type	(0) thin; (1) robust
Infertile portion shape	(0) orbicular; (1) rounded; (2) linear
Fertile portion shape	(0) lobate; (1) deeply lobate
Orientation of horns microsporophylls	(0) straight; (1) curved
Angle between horns of microsporophylls	(0) acute; (1) obtuse; (2) right
Ovulate strobilus position	(0) erect; (1) pendulous
Ovulate strobilus color (trichomes)	(0) light brown; (1) blackish to dark; (2) brown; (3) reddish brown; (4) greyish; (5) wine
Ovulate strobilus apex	(0) acuminate; (1) acute; (2) mucronate; (3) apiculate
Ovulate strobilus shape	(0) cylindrical; (1) globose
Angle between horns of megasporophylls	(0) acute; (1) right; (2) obtuse
Megasporophylls horns type	(0) thin; (1) robust
Direction of horns megasporophylls	(0) non-recurved; (1) recurved
Orientation of horn horns megasporophylls	(0) straight; (1) curved
Distal face of megasporophylls shape	(0) truncate; (1) prominent
Sarcotesta color	(0) whitish yellow; (1) whitish red
Seed shape	(0) ovate; (1) globose; (2) spherical

**Figure 3.**
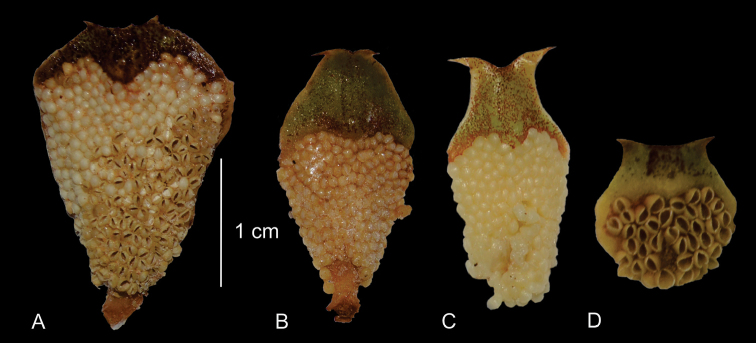
Comparison of qualitative characters of microsporophylls **A***Ceratozamiafuscoviridis***B***C.brevifrons***C***C.chamberlainii***D***C.kuesteriana*.

In microsporophylls, seven qualitative characters have been described: 1) microsporophyll shape, 2) distal face of the microsporophyll, 3) microsporophyll horn type, 4) infertile apical portion shape, 5) fertile portion shape, 6) direction of microsporophyll horns, and 7) angle between the horns of the microsporophylls. The direction of horns was coded in relation to the base of the infertile portion in the microsporophyll. In addition, six quantitative characters have been evaluated: 1) length of microsporophylls, 2) width of microsporophylls, 3) number of microsporangia per microsporophyll, 4) horn length of microsporophylls, 5) distance between the horns of a microsporophyll, and 6) length of the infertile apical portion of a microsporophyll (Figs 2, 3; see Suppl. material 1 for details).

**Figure 4.**
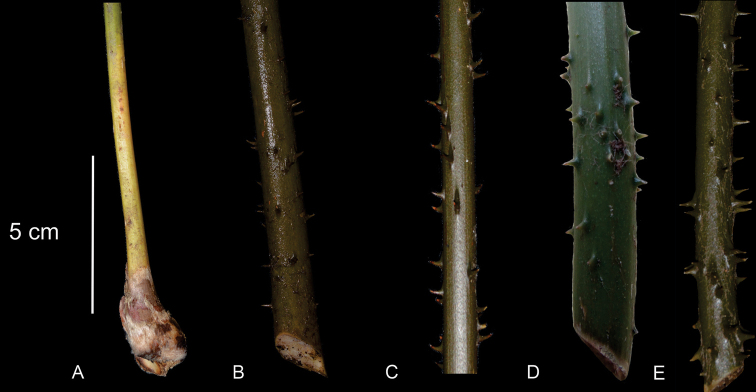
Type and number of prickles on the petiole **A** unarmed **B** thin prickles **C** robust and long prickles **D** robust and short prickles **E** bifurcate.

The position of leaves is a character that refers to the arrangement of the leaves in relation to the stem apex. The leaves are ascending when they are oriented obliquely upward or with the upper half of the leaf curved apically. The prickles on the petioles of leaves can be robust when the prickle is abruptly tapered from the base to the apex or thin when the shape is homogenous or is gradually tapering. In several species, the prickles are grouped and can bifurcate. The number of prickles differs among species; this character is most conspicuous at the base of the petiole. Here, the prickles were counted along 10 cm from the base to distal end on the petiole (Fig. 4).

## ﻿Results

### ﻿Circumscription

**Figure 5.**
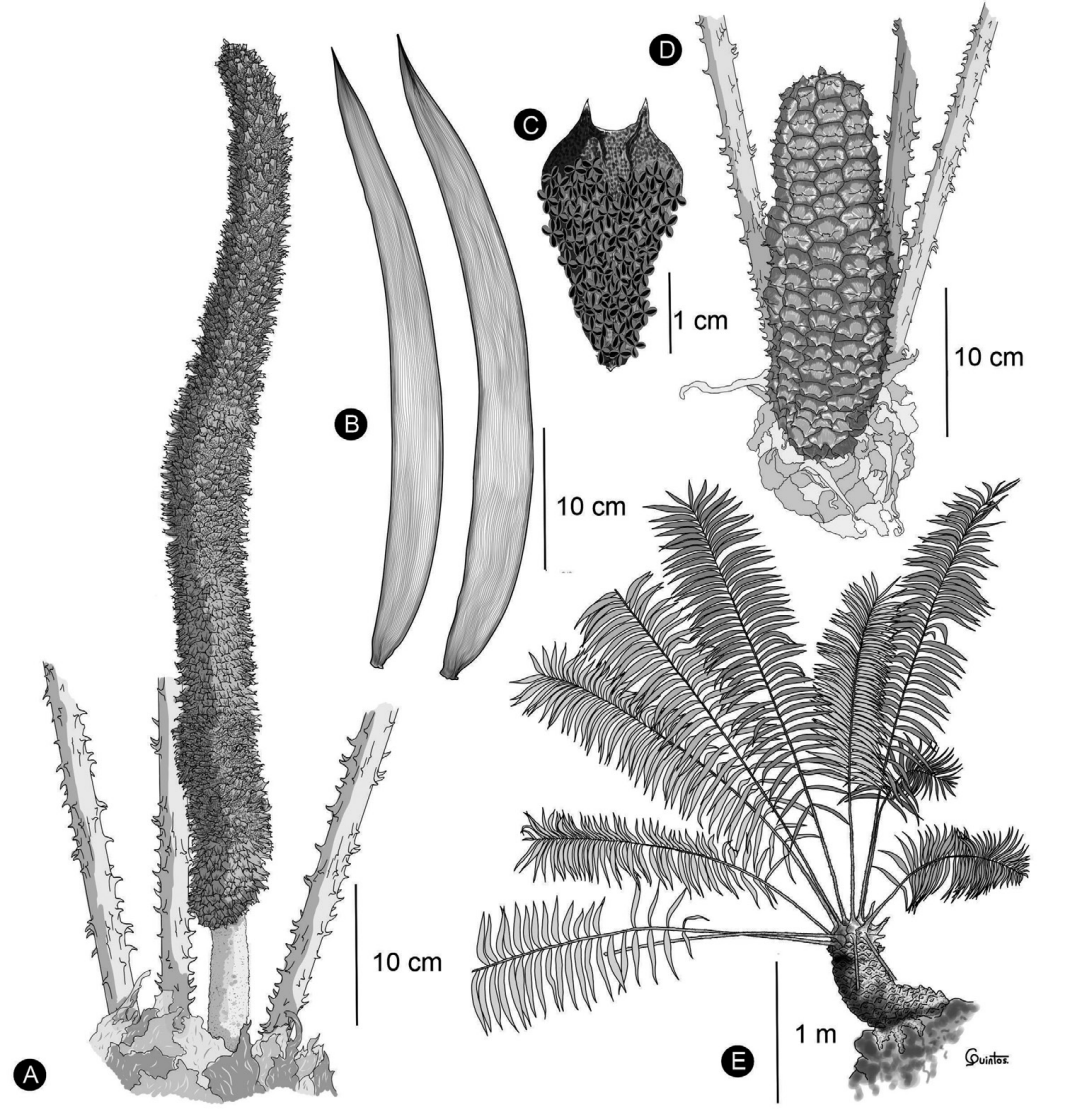
Illustration of *Ceratozamiaosbornei***A** pollen strobilus at maturity **B** variation of leaflets **C** microsporophyll **D** ovulate strobilus at maturity **E** adult plant **A–C** based on *B. K. Holst 4105* and **D, E** based on cultivated plant in Fairchild Tropical Botanic Garden.

**Figure 6.**
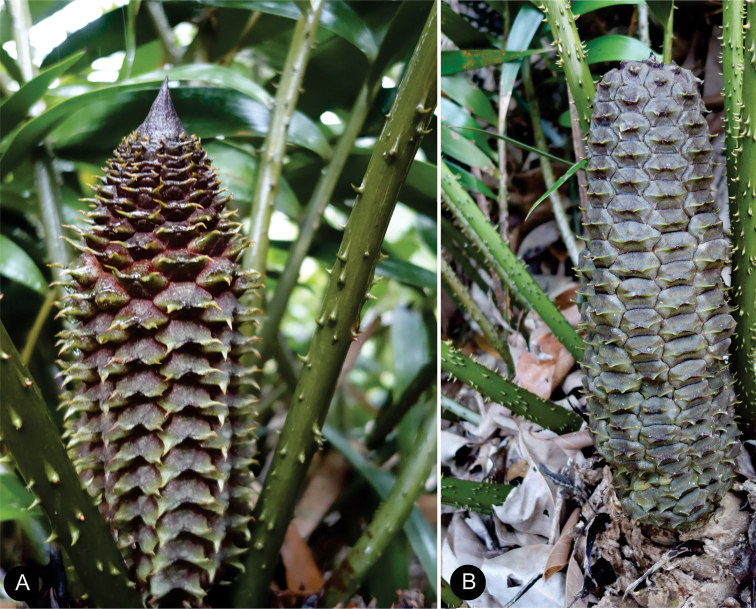
Ovulate strobilus of *Ceratozamiaosbornei***A** immature ovulate strobilus **B** mature ovulate strobilus. These photos were taken in Fairchild Tropical Botanic Garden.

We recognize 36 species in *Ceratozamia*, including one new species from Belize, characterized by megasporophylls covered by abundant purple to wine-colored trichomes present throughout the ontogeny of the strobilus, the acuminate apex of its ovulate strobili, and pollen strobili with a long infertile portion from 0.65 to 0.80 cm long (Figs 5, 6).

### ﻿Habit

**Figure 7.**
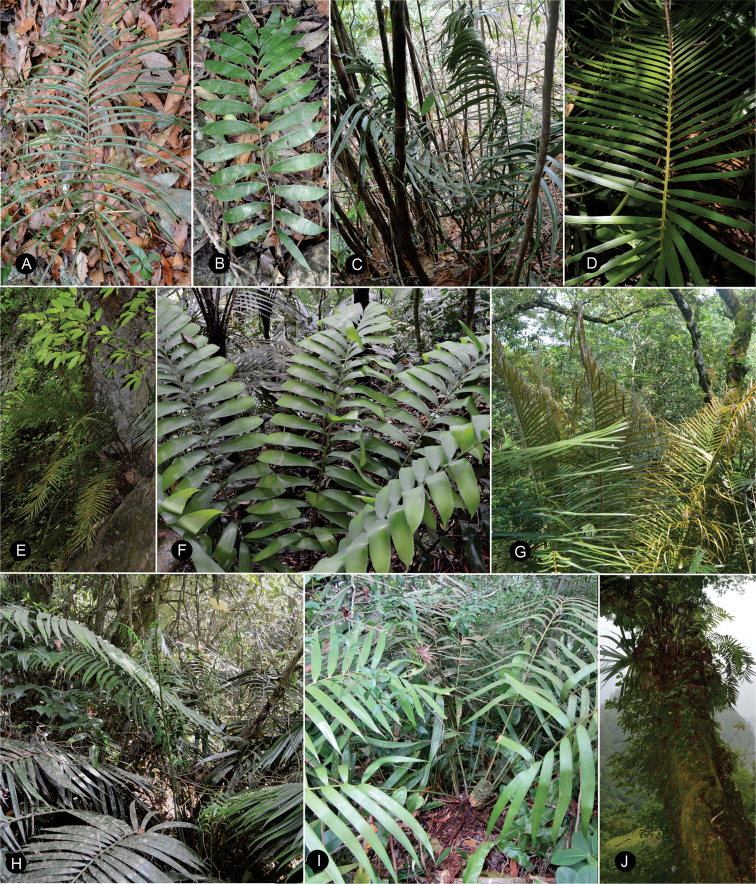
Plants of *Ceratozamia* in habitat **A***C.kuesteriana***B***C.latifolia***C***C.leptoceras***D***C.matudae***E***C.mexicana***F***C.miqueliana***G***C.mirandae***H***C.mixeorum***I***C.morettii***J** Epiphytic *C.morettii*.

The species are terrestrial or rupicolous. *Ceratozamiabrevifrons* Miq., *C.morettii* Vázq.Torres & Vovides and *C.tenuis* (Dyer) D.W.Stev. & Vovides are terrestrial, but all three species can occasionally be found as epiphytes (Fig. 7J). We found ovuliferous plants of *C.morettii* growing as epiphytes in Veracruz, Mexico. Adult individuals have been recorded growing up to 8 meters in height from the base of a host tree. All *Ceratozamia* species have a pachycaulous stem and are epigeous or semi-epigeous. Most of the species have erect stems, which may branch dichotomously with age. Only four species have an arborescent appearance, with stems of up to 2.5 meters long (*C.aurantiaca* Pérez-Farr., Gut.Ortega, J.L.Haynes & Vovides, *C.osbornei* sp. nov., *C.robusta* Miq., and *C.subroseophylla* Mart.-Domínguez & Nic.-Mor.). Some plants of *C.subroseophylla* have a stem up to 5 meters long but decumbent.

### ﻿Vegetative morphology

**Figure 8.**
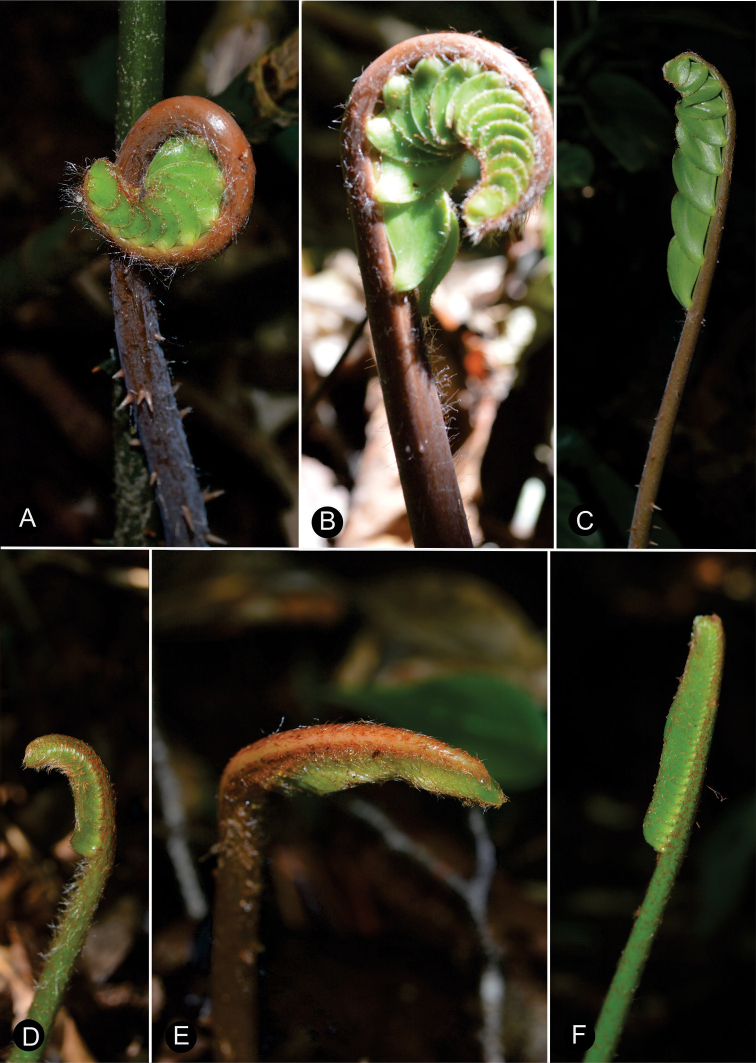
Ptyxis in *Ceratozamia***A–C***Ceratozamiamiqueliana***D–F***C.matudae*.

**Figure 9.**
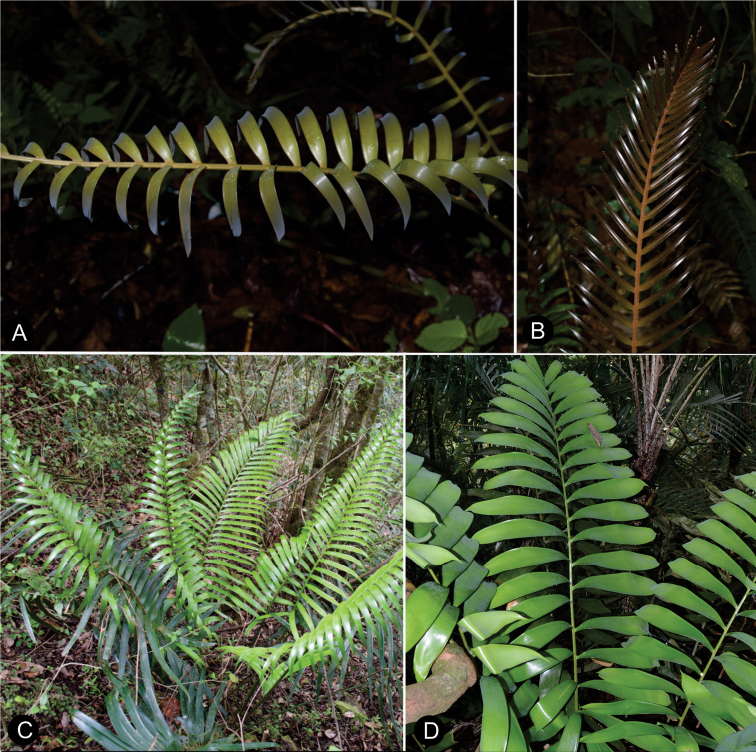
Color of leaves at emergence **A***Ceratozamiasancheziae***B***C.kuesteriana***C***C.fuscoviridis***D***C.miqueliana*.

The ptyxis of leaves can be inflexed as in *C.matudae* Lundell or circinate as in *C.miqueliana* H.Wendl. (Fig. 8). The petiole of leaves elongates during leaf growth while the leaflets are progressively expanded. In the early stages of elongation, the apical end of the leaf becomes reflexed and subsequently straightens. During their development, the leaves have trichomes that are unbranched and brown or white in color. The trichomes are shed during elongation of the leaf. The color of new leaves can be green to yellowish or brown to reddish brown (Fig. 9). This leaf coloration disappears at maturity, except for a few species such as *C.chamberlainii* Mart.-Domínguez, Nic.-Mor. & D.W.Stev., in which the color remains at the base of the leaflets, margin and rachis. The emerging leaf color is generally homogeneous among individuals in a population and within a species. However, this character is polymorphic in *C.fuscoviridis* W.Bull. and *C.sancheziae* Pérez-Farr., Gut.Ortega & Vovides where different individual plants may have brown or green leaves at emergence.

**Figure 10.**
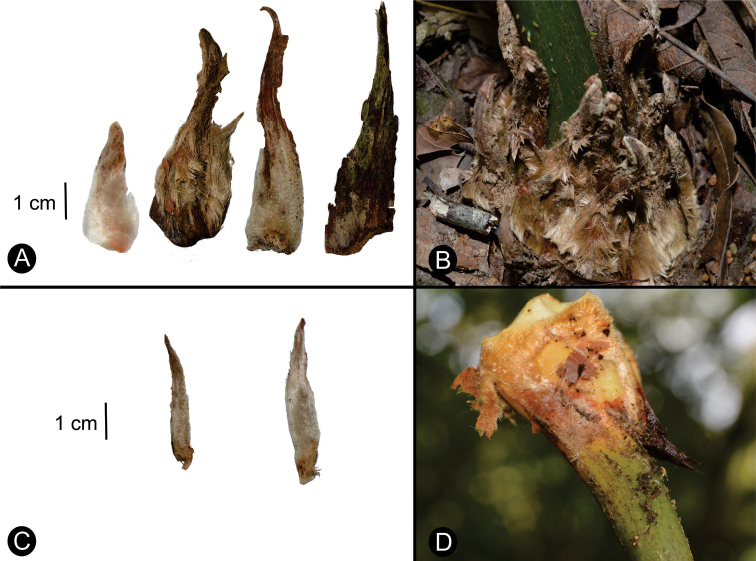
Shapes and pubescence of cataphylls and stipules **A** from left to right: *Ceratozamialatifolia*, *C.delucana*, *C.morettii* at emergence and *C.morettii* at maturity **B** cataphylls of *C.delucana***C** stipules of *C.morettii***D** stipules of *C.matudae*.

**Figure 11.**
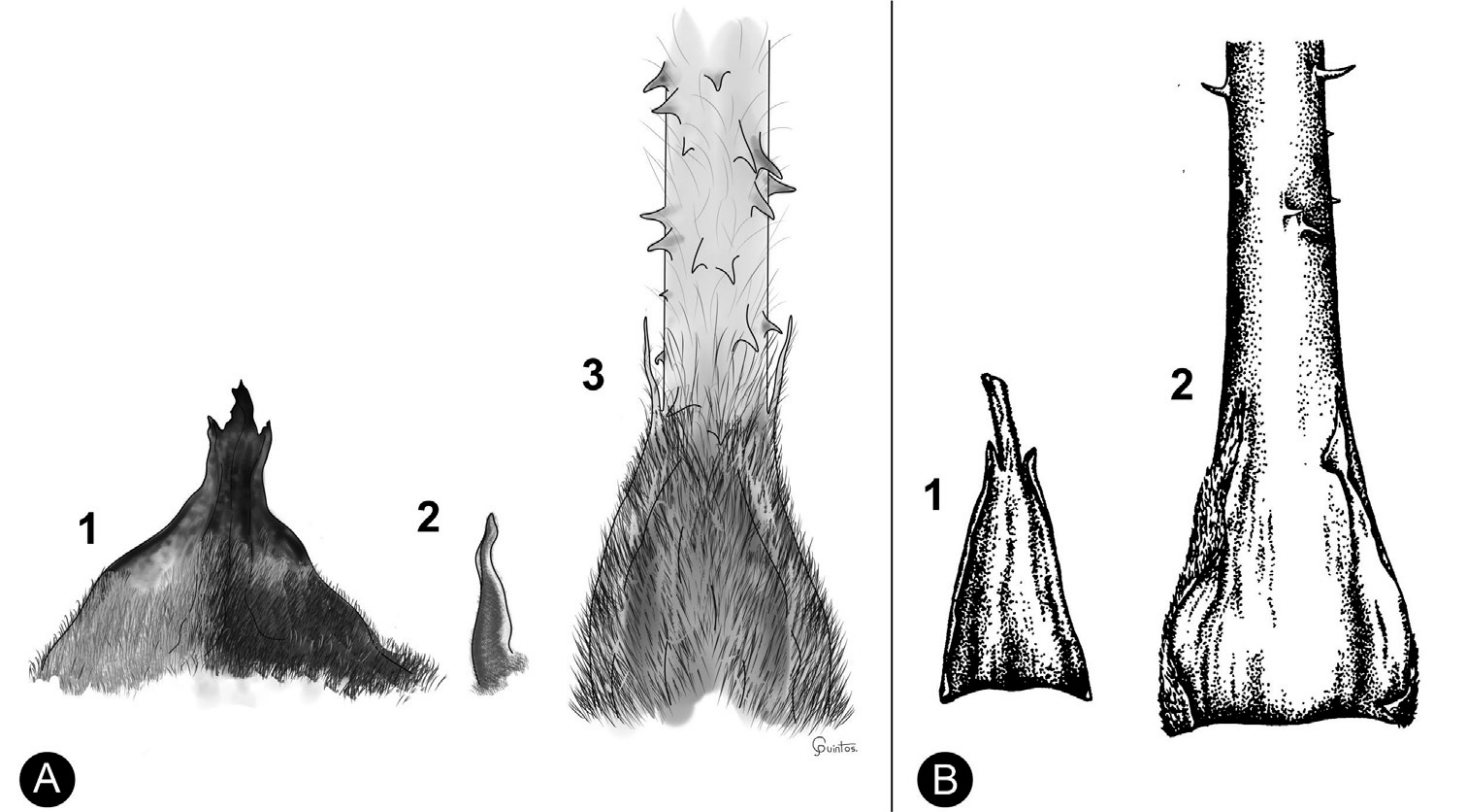
Shape and position of cataphylls and stipules of *Ceratozamia***A***C.miqueliana* (1) Cataphylls (2–3) Stipules **B***C.mexicana* from Stevenson (1981). (1) Cataphylls. (2) Stipules.

*Ceratozamia* has cataphylls and well-developed stipules (Stevenson 1981, 1992). The stipules are positioned on the base of petioles as a winglike structure with each stipule having two free tips. These stipules, which encircle shoot apex, have a linear shape and are covered by light brown trichomes (Figs 10C, D, 11) and are usually between 1.5 to 5 cm long and 0.4 to 2 cm wide. Cataphylls are foliar organs that emerge from apex of stem associated with reproductive events. Their development occurs in a series of two or more cataphylls. These are brown to reddish brown and triangular to narrowly triangular and covered by scarce or abundant brown or dark brown trichomes (Figs 10A, B, 11). They become glabrous or partially glabrous at maturity (Fig. 10A).

### ﻿Reproductive morphology

Pollen grains are sulcate, and exine surface and sexine nearly identical (Dehgan and Dehgan 1988). Some slight differences in measurements of grains have been found; however, the ornamentation and shape of pollen are similar in all species (Vovides et al. 2021).

The ovulate and pollen strobili have two horns at the distal end of each sporophyll. The ovulate strobili show minimal variation at the species level. In most species, the ovulate strobilus shape is cylindrical, whereas in *Ceratozamiamorettii* and *C.matudae* it is globose. However, the color of trichomes, apex shape, angle between horns and the distal face of megasporophylls are useful for discriminating between species (Table 1). In contrast, the pollen strobili possess distinctive characters for identifying several species including closely related taxa (Table 1). The microsporophyll horns can be thin or robust, and show different orientations (i.e., straight or recurved). *C.sabatoi* Vovides & Vázq.Torres is the only species with recurved microsporophylls with a downward distal face (Fig. 1A).

Generally, the characters of reproductive structures exhibit little variation within populations. However, these characters are polymorphic in some species such as *Ceratozamiadelucana* Vázq.Torres, A.Moretti & Carv.-Hern., in which the infertile apical portion shape of microsporophylls can be orbicular or discoid in the same population. Other species with a similar variation pattern are *C.alvarezii* Pérez-Farr., Vovides & Iglesias and *C.mirandae* Vovides, Pérez-Farr. & Iglesias. Both of these species have an angle between the horns of microsporophylls that is either obtuse or acute.

**Figure 12.**
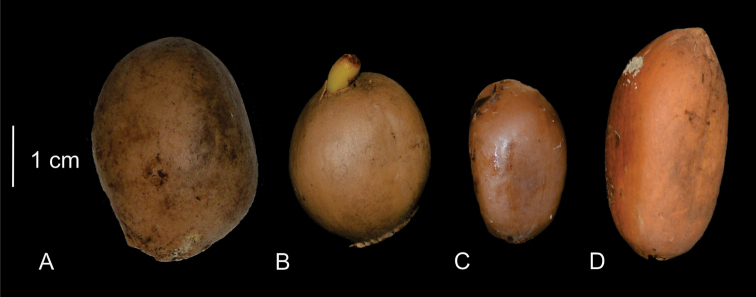
Seeds of *Ceratozamia***A***C.sancheziae***B***C.alvarezii***C***C.brevifrons.***D***C.subroseophylla*.

The seeds are very similar in all species. The most common shape is ovate that can be somewhat irregular, whereas some species such as *Ceratozamiaalvarezii* and *C.mirandae* have spherical seeds, and *C.matudae* and *C.sancheziae* have globose seeds (Fig. 12). Seeds are brown to light brown and are between 1.5 and 2.5 cm long, although some species have seeds up to 3.8 cm long (e.g., *C.subroseophylla*).

### ﻿Phenology

Plants produce a new flush of leaves annually or biannually in a low spiral. The leaves emerge in flushes of from 2, as *C.latifolia* Miq., to 20 as in adult plants of *C.osbornei* and *C.subroseophylla*. However, the plants of most species produce between six and 12 leaves. Generally, the leaves emerge during January to March. Some plants at population level may produce leaves during August to September.

The ovulate and pollen strobili emerge from among the cataphylls. Polliniferous plants produce between 1 to 2 strobili per apex, whereas ovulate plants produce only 1 strobilus per apex and very rarely two in vigorous large plants such as *Ceratozamiaosbornei* and *C.mexicana*, particularly in cultivation. We found up to 5 pollen strobili in the same plant during a reproductive season; generally, these pollen strobili emerge within 1 to 2 months of each other. We have recorded very few ovulate plants with more than one strobilus. The phenology shows a slightly different pattern between species. Generally, the strobili emerge between July and September. However, in some species the emergence can occur two months later (e.g., *C.miqueliana*).

### ﻿Distribution, endemism and habitat

**Figure 13.**
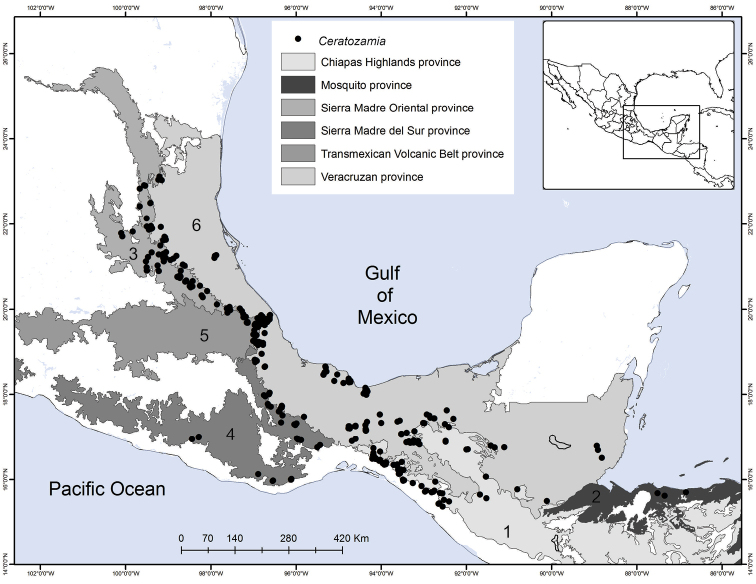
Distribution of Ceratozamia genus. Biogeographic regionalization of the Neotropical region according to Morrone et al. (2022).

*Ceratozamia* occurs from Mexico to Guatemala, Belize and Honduras in Central America. According to the biogeographic regionalization of the Neotropical region (Morrone et al. 2022) *Ceratozamia* occurs in Mexican Transition Zone and Mesoamerican dominion of Brazilian subregion (Fig. 13). In the first, there are 69% (25 of 36 species) of the species, which are distributed in Sierra Madre Oriental (SMO), Sierra Madre del Sur (SMS), Transmexican Volcanic Belt Province (TVBP), and Chiapas Highlands (CH). The SMO and CH are the provinces with the highest concentration of species richness in the genus (8 species in each province). The SMS and TVBP have 5 (*C.aurantiaca*, *C.leptoceras* Mart.-Domínguez, Nic.-Mor., D.W.Stev. & Lorea-Hern., *C.mixeorum* Chemnick, T.J.Greg. & Salas-Mor., *C.oliversacksii* D.W.Stev., Mart.-Domínguez & Nic.-Mor., and *C.whitelockiana* Chemnick & T.J.Greg.) and 4 species, respectively (*C.delucana*, *C.mexicana*, *C.morettii* and *C.tenuis*).

In the Mesoamerican dominion of the Brazilian subregion, species occur in two provinces: Veracruzan province and Mosquito province. In Veracruzan province 14 species are present (*C.becerrae* Pérez-Farr., Vovides & Schutzman, *C.brevifrons*, *C.chimalapensis*, *C.decumbens* Vovides, Avendaño, Pérez-Farr. & Gonz.-Astorga, *C.delucana*, *C.euryphyllidia* Vázq.Torres, Sabato & D.W.Stev., *C.huastecorum* Avendaño, Vovides & Cast.-Campos, *C.mexicana*, *C.miqueliana*, *C.robusta*, *C.santillanii* Pérez-Farr. & Vovides, *C.subroseophylla*, *C.osbornei*, and *C.zoquorum* Pérez-Farr., Vovides & Iglesias), whereas in Mosquito province only a single species (*C.hondurensis* J.L.Haynes, Whitelock, Schutzman & R.S.Adams) occurs. In particular, *C.mexicana* and *C.delucana* also occur in TVBP and *C.robusta* in CH and Veracruzan provinces. *Ceratozamia* has not been reported from the Yucatán Peninsula province (Fig. 13); we believe this is due to lack of collections in the southwest of Yucatán (Mexico). The apparent disjunct pattern from El Petén Department (Guatemala) may be explained by this collection deficiency.

With 32 endemic species of *Ceratozamia*, Mexico is the center of endemism of the genus. Only four species are occur in Central American countries: *C.hondurensis* from Honduras; *C.osbornei* from Belize; and *C.robusta* and *C.vovidesii* Pérez-Farr. & Iglesias from Guatemala. Some species have a broad geographic range, while others occur in a very limited area. Most of the species that have narrower distributions can be considered micro-endemics, such as *C.alvarezii* and *C.morettii* in Chiapas States and Central Veracruz from Mexico, respectively. In contrast, *C.robusta* has a broad distribution, with a range from northwest Chiapas (Mexico) to Guatemala. Other species of the genus with broad distributions are *C.miqueliana* and *C.subroseophylla*. The latter species has been considered endemic to the montane region of Los Tuxtlas (State of Veracruz, Mexico). However, we found populations at southwest of Veracruz in the Uxpanapa region and Tabasco State, which represent an extension of its geographical range. In particular, the population of *C.subroseophylla* from Tabasco represents a new record for this Mexican state.

Montane regions show the highest diversity of *Ceratozamia* species. The distributional pattern is congruent with the existence of the main mountain systems of Mexico. The mean elevation for *Ceratozamia* species is 1,100 m (range 19–2,000 m), with the majority of the species occurring between 800 to 1,200 m. *Ceratozamiamiqueliana*, occurs at 19 meters of elevation in lowland vegetation of Veracruz, while *C.mixeorum* and *C.zaragozae* Medellín are the species that occur at higher elevations above 1,200 m of Oaxaca and San Luis Potosí, respectively.

Species richness in *Ceratozamia* tends to be correlated with moister habitats, principally in limestone rock areas. The genus is found in four vegetation types: cloud forest, evergreen tropical forest, oak forest and oak-pine forest. Most species inhabit cloud forest, whereas in oak-pine forest there are only a few species. No species is known from dry forest. Some species do grow near to rivers or lagoons, e.g., *C.aurantiaca* and *C.miqueliana* near the Santo Domingo River and Majahual lagoons, respectively.

## ﻿Discussion

The cycad genus *Ceratozamia* as here circumscribed includes 36 species, 34 of which are found in Mexico with 32 of those endemic to Mexico. *C.robusta* is the species with the widest distribution in the genus, occurring continuously from the central region of Chiapas State in Mexico to Guatemala. Unfortunately, herbarium specimens from Guatemala are infertile and there are few sterile specimens in herbaria in general, which has led to ambiguous and/or conflicting taxonomic identifications. Comparative vegetative morphology with these specimens suggests that *C.robusta* also has a wide elevational range between 400 meters and up to 1,300 meters of elevation. However, recently collected reproductive material has been observed for plants from Belize, which have revealed features that demonstrate that the Belize plants can be distinguished from those in Mexico and Guatemala resulting in our description of *C.osbornei*. Further population level studies of this species are needed to evaluate genetic differentiation and gene flow throughout the entire ranges of both *C.osbornei* and *C.robusta*. *C.matudae* was reported for Guatemala from the Sierra de Cuchumatanes in the 1940s, but no past or recently collected specimens have been located. Considering this paucity of information and the exceptionally rapid loss of forest cover in the region, a thorough exploration and study of these localities is essential.

Recently, Vovides et al. (2020) recircumscribed the *Ceratozamiamiqueliana* complex using leaflet anatomy and other macromorphological characters. As a result, *C.zoquorum* was recircumscribed with a much narrower taxonomic concept. They considered the presence or absence of girder sclerenchyma and a lignified hypodermis to be a significant trait to distinguish between *C.zoquorum* and *C.becerrae*, and the latter was removed from synonymy under *C.zoquorum*. However, we found that the corresponding taxonomic key failed to properly identify these closely related species with anatomical characters that are contradictory to the descriptions. Thus, there is a lack of correspondence between the key entries and homologous character states in both supposed species (Vovides et al. 2020; p. 11).

Therefore, the only two remaining anatomical characters relevant in this case are: 1) girder sclerenchyma present in *Ceratozamiazoquorum* and mostly absent in *C.becerrae*, and 2) a lignified adaxial hypodermis in *C.zoquorum* that is also absent in *C.becerrae*. According to the descriptions provided by the authors, the hypodermis in *C.becerrae* is absent only in the revolute leaflet margin with up to three layers of fibres, whereas in *C.zoquorum* such margin is discontinuous with 1–7 lignified isodiametric fibres.

In their anatomical evaluation, Vovides et al. (2020: 2) studied two cultivated individuals for evaluating phenotypic variation. The anatomical characters should therefore be characterized and reassessed under a broader approach by testing the morphological variation through different populations at the intra- and inter-population level: most leaflet macromorphological characters —including qualitative ones— often exhibit polymorphisms, as well as wide variation at the population level. Generally, leaflets exhibit high variation mainly in quantitative characters (Pérez-Farrera et al. 2009; Martínez-Domínguez et al. 2017a, c). Considering then that leaflets in *Ceratozamia* species show contrasting shapes between geographically close species, but similar shapes even when the biogeographic pattern is disjunct, we suggest that the leaflet anatomy should be re-assessed through broader sampling.

Based on our research, the taxonomic value of some traditional morphological characters —such as leaflet size— should only be secondarily important, and the focus should be put on defining qualitative characters in both leaves and leaflets, and characters derived from reproductive structures as proposed in Table 1. Regarding the latter, we observed that the position of ovulate strobilus was not consistent throughout the different ontogenetic states. Ovulate strobili can recline due to the weight of the fertile portion of ovulate strobilus and because of the position of the plant growing on a rocky wall. This is mainly because the ovulate strobilus position is correlated with the length of the peduncle of strobili and the fact that the peduncle curves at maturity. Our data support that other characters such as those of the microsporophylls and the shape of their horns can be coded into discrete character states and, therefore, provide more taxonomic utility than previously understood.

This modern taxonomic treatment is drawn from wide sampling, thorough review of specimens in collections and molecular data (Martínez-Domínguez et al. 2016, 2017c, 2020), thus establishing a framework for future research and providing a resource for disentangling confusing species. Ultimately, this work contributes to the conservation of this threatened genus. Despite advances in the taxonomy and systematics of this genus, taxon-level data are required for a complete systematic evaluation. We suggest that the use of phenological information as well as a more nuanced understanding of phenotypic evolution —including, for example, instances of plasticity and other ontogenetic development-related phenomena— that might impact diversification in species complexes or other groups of closest related species in this gymnosperm genus, will be increasingly relevant for the task.

### ﻿Taxonomic treatment

#### ﻿ Ceratozamia

Taxon classificationPlantaeCycadalesZamiaceae

Brongn., Ann. Sci. Nat., Bot. ser. 3, 5: 7, t. 1. 1846.

3884E28F-65AC-5719-9CD9-9EF5F189E17E

##### Type species.

*Ceratozamiamexicana* Brongn.

##### Description.

***Stem*** 10–250 cm long, 8–40 cm in diameter, epigeous or semi-hypogeous, erect or decumbent. ***Cataphylls*** persistent, triangular to narrowly triangular, reddish brown, tomentose to densely tomentose at emergence, partially tomentose at maturity, apex acuminate. ***Leaves*** stipulate, ascending to descending, light green or reddish brown at emergence with whitish gray or brown trichomes, generally glabrous at maturity; stipulate 2–6 cm long, linear, tomentose at maturity. ***Petiole*** straight or twisted, sometimes brown in mature leaves, without prickles or heavily to lightly armed with prickles; prickles can be bifurcate. ***Rachis*** straight or twisted, without prickles or armed with prickles up to half the length of the leaves. ***Leaflets*** articulate, sessile, membranaceous to coriaceous, linear to obovate, opposite to subopposite or clustered, not imbricate, generally acuminate at apex, attenuate at base, margins entire; articulations green to brown. ***Pollen strobili*** 1–2, with sterile tip, erect, cylindrical, green to cream with blackish to reddish brown trichomes at maturity; pollen sporangiophores deltoid to cuneate, basally stalked, distal face bicornate, fertile abaxial surface with 24–280 sporangia in clusters of (2)3(4–5), dehiscent by longitudinal slit; peduncle pubescent to tomentose. ***Ovulate strobili*** usually solitary, globose to cylindrical; green with pale pink to blackish trichomes at maturity, acute to apiculate apex; ovulate sporangiophores peltate with a narrow basal stalk and transversely hexagonal tips, bicornate at distal end; peduncle pubescent to tomentose, erect to pendulous. ***Seeds*** (ovules) 2 per megasporophyll projecting inward toward the strobilus axis, spherical, sarcotesta pink to yellowish when immature, light brown at maturity, sclerotesta smooth with several furrows longitudinal from micropylar end.

##### Distribution and habitats.

The 36 species of *Ceratozamia* are only found from Mexico to Central America, usually in montane habitats on limestone soils at elevations from 19 to 2,000 m. Most of the species are narrowly endemic, and all are on CITES Appendix I.

### ﻿Artificial key for *Ceratozamia*

**Table:** 

1	Petiole and rachis twisted	**2**
–	Petiole and rachis straight	**3**
2	Petiole and rachis with prickles	***C.norstogii***
–	Petiole and rachis unarmed	***C.zaragozae***
3	Leaflets fasciculate	***C.hildae***
–	Leaflets opposite to subopposite	**4**
4	Leaflets obovate; veins prominent	**5**
–	Leaflets oblong to linear; veins not prominent	**6**
5	Leaflets with sinuate margin at the distal end and an asymmetrical apex	***C.euryphyllidia***
–	Leaflets with entire margin at the distal end and a symmetrical apex	***C.hondurensis***
6	Leaflets oblong to oblanceolate	**7**
–	Leaflets linear to lanceolate	**19**
7	Leaflets papyraceous	**8**
–	Leaflets coriaceous	**12**
8	Petiole and rachis unarmed	***C.latifolia***
–	Petiole and rachis with prickles	**9**
9	Leaflets with a symmetric apex	***C.totonacorum***
–	Leaflets with an asymmetric or abruptly symmetrical apex	**10**
10	New leaves reddish brown at emergence; ovulate strobilus green with brown trichomes at maturity; microsporophylls with the infertile apical portion partially covered with trichomes	***C.aurantiaca***
–	New leaves green at emergence; ovulate strobilus green with black trichomes at maturity; microsporophylls with the infertile apical portion completely covered with trichomes	**11**
11	Petiole armed with robust prickles; microsporophylls rounded; ovulate strobilus with an acuminate apex	***C.miqueliana***
–	Petiole armed with thin prickles; microsporophylls orbicular; ovulate strobilus with an acute apex	***C.delucana***
12	New leaves reddish brown at emergence; ovulate strobilus with abundant wine-colored to reddish brown trichomes	**13**
–	New leaves green at emergence; ovulate strobilus with blackish brown trichomes	**14**
13	Petiole armed with robust prickles; rachis armed with prickles; ovulate strobilus more than 20 cm long with an acuminate apex	***C.chamberlainii***
–	Petiole armed with thin prickles; rachis unarmed to armed with few prickles; ovulate strobilus up to 15 cm long with an acute apex	***C.decumbens***
14	Petiole prickles less than or equal to 0.20 cm long	**15**
–	Petiole prickles more than 0.20 cm long	**18**
15	Apical leaflets less than or equal to 3.5 cm wide; brown trichomes of leaves at emergence; ovulate strobilus with an apiculate apex	***C.morettii***
–	Apical leaflets more than 3.5 cm wide; whitish gray trichomes of leaves at emergence; ovulate strobilus with an acute apex	**16**
16	Peduncle of ovulate strobilus 3 cm long or shorter	***C.santillanii***
–	Peduncle of ovulate strobilus more than 3 cm long	**17**
17	Plants from Sierra Madrigal at the border of Tabasco and Chiapas States (Mexico)	***C.becerrae***
–	Plants from northern mountains of Chiapas State (Mexico)	***C.zoquorum***
18	Leaves with 20–43 pairs of leaflets; microsporophylls 1.3 cm long or longer, 1.3 cm wide or wider; trichomes of leaves at emergence brown; 10 or more leaves per leaf crown	***C.delucana***
–	Leaves with 8–18 pairs of leaflets; microsporophylls less than 1.3 cm long, less than 1.3 cm wide; trichomes of leaves at emergence whitish gray; less than 10 leaves per leaf crown	***C.huastecorum***
19	Leaflets coriaceous	**20**
–	Leaflets papyraceous to membranaceous	**22**
20	Leaflets keeled; petiole armed with robust prickles	***C.brevifrons***
–	Leaflets plane; petiole armed with thin prickles	**21**
21	Petiole sparsely prickly (30 prickles or fewer); microsporophylls with infertile apical portion more than 0.45 cm long, horns more than 0.25 cm long	***C.mexicana***
–	Petiole abundantly prickly (more than 35 prickles); microsporophylls with infertile apical portion less than 0.45 cm, horns less than or equal to 0.25 cm	***C.delucana***
22	Leaflets involute to lightly involute or canaliculate	**23**
–	Leaflets flat	**26**
23	New leaves at emergence dark green with brown trichomes; basal leaflet more than or equal to 1 cm wide	***C.tenuis***
–	New leaves at emergence brown to reddish brown with whitish gray trichomes; basal leaflet less than 1 cm wide	**24**
24	Petiole sparsely prickly (less than or equal to 10 prickles); prickles short (less than or equal to 0.15 cm); microsporophylls discoid, up to 1.1 cm long	***C.kuesteriana***
–	Petiole abundant prickly (more than 10 prickles); prickles long (more than 0.15 cm); microsporophylls elliptic, more than 1.2 cm long	**25**
25	Leaves with rachis the same length or shorter than the petiole; plants from Cintalapa and Juquipilas municipalities in Chiapas State (Mexico)	***C.alvarezii***
–	Leaves with rachis longer than petiole; plants from Sierra Morena and Tres Picos in Chiapas State (Mexico)	***C.mirandae***
26	Petiole armed with robust prickles	**27**
–	Petiole armed with thin prickles	**29**
27	Leaflets abaxially curved in both median and apical portions; ovulate strobilus dark green with scarce blackish trichomes or glabrous at maturity; microsporophylls with curved horns	***C.robusta***
–	Leaflets abaxially curved in the median and mostly planar in the apical portion; ovulate strobilus green with abundant trichomes reddish brown to purple at maturity; microsporophylls with straight horns	**28**
28	Ovulate strobilus with base pale pink megasporophylls and dark brown to reddish brown trichomes at maturity; microsporophylls with a lobate fertile portion and infertile portion up to 0.65 cm long	***C.subroseophylla***
–	Ovulate strobilus with green megasporophylls base and dark purple to wine trichomes at maturity; microsporophylls with a deeply lobate fertile portion and infertile portion more than 0.65 cm long	***C.osbornei***
29	Leaflets linear	**30**
–	Leaflets lanceolate	**31**
30	Leaflets membranaceous	***C.leptoceras***
–	Leaflets papyraceous	**32**
31	Base of leaflets and articulations yellow	***C.matudae***
–	Base of leaflets and articulations green to brown	**34**
32	Prickles on the petiole more than or equal to 0.50 cm long; horns of microsporophylls thin	***C.oliversacksii***
–	Prickles on the petiole less than or equal 0.34 cm long; horns of microsporophylls robust	**33**
33	Leaflets less than or equal 1.5 cm wide; recurved downward distal face of microsporophylls	***C.sabatoi***
–	Leaflets more than 1.5 cm wide; non-recurved distal face of microsporophylls	***C.sancheziae***
34	New leaves green to yellowish green	**35**
–	New leaves brown to reddish brown	**36**
35	Rachis unarmed	***C.whitelockiana***
–	Rachis with prickles	**37**
36	Microsporophylls with an orbicular infertile portion; megasporophylls with a truncate distal face	***C.fuscoviridis***
–	Microsporophylls with a rounded to linear infertile portion; megasporophylls with a prominent distal face	**39**
37	Leaves descending	***C.mixeorum***
–	Leaves ascending	**38**
38	Leaflets more than or equal 2.3 cm wide	***C.delucana***
–	Leaflets less than 2.3 cm wide	***C.fuscoviridis***
39	Microsporophylls elliptic with curved horns	***C.chimalapensis***
–	Microsporophylls obconic with straight horns	***C.vovidesii***

#### ﻿1. Ceratozamia alvarezii

Taxon classificationPlantaeCycadalesZamiaceae

Pérez-Farr., Vovides & Iglesias, Novon 9: 410. 1999

9A200C6C-5D7A-52C0-BA61-39507296112C

Figs 12B , 15A

##### Type.

Mexico. Chiapas: Mun. Cintalapa, Rancho El Cafetal, 950 m, 4 Mar 1996, *M.A. Pérez-Farrera 889* (holotype: CHIP! [acc. # 14306]; isotypes: F! [acc. # 2193633], HEM! [acc. # HEM004830], MEXU! [MEXU00827362], MO! [acc. # 04882667]).

##### Description.

***Stem*** 20–60 cm long, 15–30 cm in diameter, epigeous, erect and decumbent. ***Cataphylls*** 2.0–5.0 × 1.5–3.0 cm wide at the base, persistent, triangular, reddish brown, densely brownish tomentose at emergence, partially tomentose at maturity, apex acuminate. ***Leaves*** 2–30 (40), 36–125 cm long, ascending, brown at emergence with whitis gray trichomes, glabrous at maturity. ***Petiole*** 10–60 cm long, terete, linear, brown in mature leaves; with 11–40 thin prickles, 0.19–0.37 cm long. ***Rachis*** 25–87 cm long, terete, linear, brown to greenish brown in mature leaves, with prickles. ***Leaflets*** 25–68 pairs, opposite to subopposite, insertion in one plane, linear to lanceolate, generally longitudinally planar, not basally falcate, papyraceous, slightly involute, green with adaxial and abaxial sides glabrous, distal end with entire margins, acuminate and symmetric at the apex, attenuate at base, with conspicuous and light-green veins; median leaflets 12–38 × 0.3–0.9 cm, 0.14–1.30 cm between leaflets; articulations 0.14–0.75 cm wide, brown. ***Pollen strobili*** 15–40 cm long, 3.5–5.1 cm in diameter, solitary, cylindrical, erect, yellowish green with brown trichomes at emergence, yellowish cream with blackish brown trichomes at maturity; peduncle 4–10 cm long, 1.5–2.1 cm in diameter, reddish brown to brown pubescent; microsporophylls 1.24–1.56 × 0.59–0.83 cm, elliptic with a non-recurved distal face and a lobate fertile portion, infertile portion 0.50–0.81 cm long and linear with straight horns 0.14–0.30 cm long, 0.45–0.90 cm and an obtuse to acute angle between the horns. ***Ovulate strobili*** 17–27 cm long, 7.2–12.3 cm in diameter, solitary, cylindrical, erect, yellowish green with abundant blackish trichomes at emergence, glaucous green with reddish brown to blackish trichomes at maturity, acuminate apex; peduncle 4.0–10 cm long, 1.1–2.2 cm in diameter, erect, with scarce brownish tan trichomes; megasporophylls 20–80, 4–8 orthostichies with 5–10 sporophylls per orthostichy, 2.15–2.80 × 3.90–6.30 cm, with a prominent distal face, horns straight and thin and 0.32–0.51 cm long, 0.69–1.80 cm between horns with an obtuse angle between the horns. ***Seeds*** 2.5–3.0 cm long, 2.3–2.9 cm in diameter, spherical, sarcotesta whitish pink when immature, light brown at maturity.

##### Distribution and habitat.

**Figure 14.**
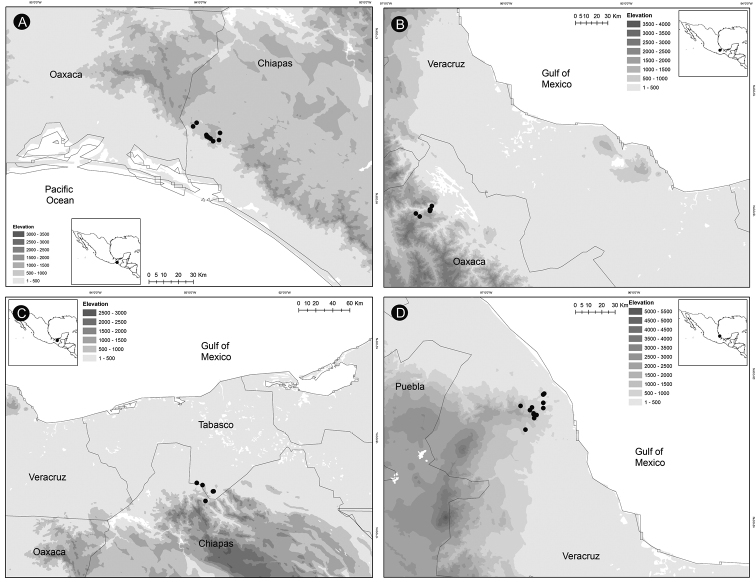
Distribution of *Ceratozamia* species **A***C.alvarezii***B***C.aurantiaca***C***C.becerrae***D***C.brevifrons*.

*Ceratozamiaalvarezii* is endemic to Mexico and only known from Cintalapa and Jiquipilas municipalities in Chiapas State, at the transition zone between pine and oak forest and oak forest; plants occur on karstic rocks between 900 and 1,450 m elevation (Fig. 14A).

##### Etymology.

The specific epithet honors Miguel Álvarez del Toro in recognition of its outstanding contributions for conservation in Chiapas and the establishment of its first reserves (Pérez-Farrera et al. 1999).

##### Common names.

Mexico. Chiapas: Espadaña cimarrona (*L. Martínez-Domínguez et al. 1370*); palma, palmita (*M.A. Pérez-Farrera 889*).

##### Uses.

The seeds are used as food (*M.A. Pérez-Farrera 889*).

##### Conservation status.

(IUCN 2021). Endangered; A2ac; B1ab(I,iii)+2ab(i,iii); C1.

##### Discussion.

The morphology of *Ceratozamiaalvarezii* is not very different from *C.mirandae*. At population level, this species differs from *C.mirandae* by having generally shorter leaves, smaller ovulate strobilus (up to 27 cm long and 5 to 10 sporophylls per orthostichy), and longer seeds.

##### Specimens examined.

Mexico. **Chiapas: Mun. Cintalapa**, 1,100 m, 10 Mar 1993, *A.P. Vovides 1234* (XAL); 920 m, 21 Jun 2018, *F. Nicolalde-Morejón et al. 2791–2799* (CIB); 980 m, 22 Jun 2018, *F. Nicolalde-Morejón et al. 2830* (CIB); 1,107 m, 18 Jun 2019, *F. Nicolalde-Morejón & L. Martínez-Domínguez 3177–3183* (CIB); 1,350 m, 18 Jun 2019, *F. Nicolalde-Morejón & L. Martínez-Domínguez 3186–3196* (CIB); 1,450 m, 11 Oct 1994, *J. Castillo et al. 445* (CHIP); 920 m, 21 Jun 2018, *L. Martínez-Domínguez et al. 1359–1369* (CIB), *1370* (CIB, MEXU); 980 m, 22 Jun 2018, *L. Martínez-Domínguez et al. 1402* (CIB, MEXU); 925 m, 18 Jun 2019, *L. Martínez-Domínguez & F. Nicolalde-Morejón 1770* (CIB); 1,107 m, 18 Jun 2019, *L. Martínez-Domínguez & F. Nicolalde-Morejón 1771,1772* (CIB, MEXU), *1773* (CIB), *1774* (CIB, MEXU), *1775–1777* (CIB); 1,350 m, 18 Jun 2019, *L. Martínez-Domínguez & F. Nicolalde-Morejón 1784*–*1794* (CIB); 900 m, 10 Jul 1994, *M.A. Pérez-Farrera 71* (CIB, CHIP, MEXU); 950 m, 5 Sep 1995, *M.A. Pérez-Farrera 776* (CIB, XAL); 1,000 m, 27 Mar 2000, *O. Farrera S. 2068* (CHIP). **Mun. Jiquipilas**, 1,200 m, 6 Jun 2002, *A. Reyes-García 5017* (MEXU); 1,380 m, 17 Feb 2000, *E. Palacios E. 2469* (CHIP); 1,170 m, 7 Jul 1994, *M.A. Pérez-Farrera 68* (CIB).

#### ﻿2. Ceratozamia aurantiaca

Taxon classificationPlantaeCycadalesZamiaceae

Pérez-Farr., Gut.Ortega, J.L.Haynes & Vovides, Taxonomy 1: 249. 2021

5E0430B3-E8B7-5713-A346-5D69A948237E

Figs 1C , 15B

Ceratozamia martinezii Mart.-Domínguez, Nic.-Mor. & D.W.Stev., Nordic J. Bot. 1: 2. 2021 [2022]. Type. Mexico. Oaxaca: Mun. San Pedro Teutila, El Faro, 615 m, 28 Sep 2020, *L. Martínez-Domínguez et al. 2049* ♀ (holotype: CIB! [acc. # 22845UV]; isotypes: MEXU!, NY!).

##### Type.

Mexico. Oaxaca: Mun. San Pedro Teutila, Sierra Norte, 30 May 2021, *M.A. Pérez-Farrera & P. Díaz-Jiménez 4014* (holotype: HEM [n.v.]; isotype: XAL [n.v.]).

##### Description.

***Stem*** 30–250 cm long, 10–15 cm in diameter, epigeous, erect or sometimes decumbent. ***Cataphylls*** 3.6–6.5 × 2–3.3 cm wide at the base, persistent, triangular, reddish brown, densely brown tomentose at emergence, glabrous at maturity, apex acuminate. ***Leaves*** 7–30 (50), 117–240 cm long, ascending, reddish brown at emergence, with whitish gray trichomes, glabrous at maturity. ***Petiole*** 45–85 cm long, terete, linear, reddish brown or greenish brown at emergence, dark green in mature leaves; with 16–47 thin prickles, 0.30–0.66 cm long. ***Rachis*** 70–160 cm long, terete, linear, dark green in mature leaves, with prickles in lower third. ***Leaflets*** 12–38 pairs, opposite to subopposite, insertion in one plane, oblong, generally longitudinally planar, not basally falcate, papyraceous, flat, green with adaxial and abaxial sides glabrous, distal end with entire margins, abruptly acuminate and symmetrical at the apex, attenuate at base, veins conspicuous and light-green; median leaflets 21–42.5 × 3.3–4.8 cm, 3.2–6.0 cm between leaflets; articulations 0.9–1.5 cm wide, green. ***Pollen strobili*** 20–35 cm long, 3.1–4.6 cm in diameter, solitary, cylindrical, erect, greenish with reddish trichomes at emergence, greenish yellow with reddish brown trichomes at maturity; peduncle 7.0–15 cm long, 1.5–2.1 cm in diameter, pubescent, reddish brown; microsporophylls 1.50–2.2 × 0.98–1.30 cm, obconic with a non-recurved distal face and a lobate fertile portion, infertile portion rounded and 0.47–0.84 cm long with horns straight and 0.16–0.38 cm long, 0.50–0.80 cm between horns with an acute angle between the horns. ***Ovulate strobili*** 11–36 cm long, 9.4–12.5 cm in diameter, solitary, cylindrical, erect, greenish yellow with an abundant reddish trichomes at emergence, green with brown trichomes at maturity, with an acute apex; peduncle 5.0–19.5 cm long, 1.3–2.3 cm in diameter, erect, light brown pubescent; megasporophylls 20–200, 5–11 orthostichies with 4–19 sporophylls per orthostichy, 2.25–2.72 × 3.6–4.3 cm, with a truncate distal face, horns curved and 0.54–0.90 cm long, 1.05–1.77 cm between horns with an acute angle between the horns. ***Seeds*** 2.4–3.0 cm long, 1.4–1.7 cm in diameter, ovate, sarcotesta whitish yellow to yellow when immature, light brown at maturity.

##### Distribution and habitat.

*Ceratozamiaaurantiaca* occurs in highlands south of Río Santo Domingo and north of Sierra Norte in Oaxaca State, Mexico (Fig. 14B), where it occurs in the evergreen tropical forest on karstic rocks at 458–800 m.

##### Etymology.

The specific epithet alludes to the leaf color at emergence. This is derived from Latin word aurantiacus for the orange color of emerging leaves.

##### Common names.

None recorded.

##### Uses.

People use the leaves of *Ceratozamiaaurantiaca* to make flower arrangements in wreaths (Martínez-Domínguez et al. 2022a).

##### Preliminary conservation status.

We visited three populations for *Ceratozamiaaurantiaca* in which we recorded between 100 to 300 adult plants. We observed seedlings, juveniles and reproductive individuals. However, one of these populations is in a risk area due to anthropogenic land-use changes and was affected by fire in 2018. In addition, it is not found within protected areas. Based upon this information, *C.aurantiaca* should be considered “Endangered (EN)” in accordance with IUCN criteria.

##### Discussion.

*Ceratozamiaaurantiaca* is most similar to *C.whitelockiana*, however, differs from it in its oblong leaflets abruptly acuminate with a symmetric apex, its petiole with abundant (16–24) and long prickles (0.32–0.55 cm), and its rachis that has prickles in the proximal third of the leaf. In addition, the ovulate strobili have an acute apex, and the pollen strobili have obconic microsporophylls with a rounded infertile portion. The leaves are reddish brown at emergence, whereas in *C.whitelockiana* they are green.

##### Specimens examined.

Mexico. **Oaxaca: Mun. Chiquihuitlán de Benito Juárez**, 861 m, 26 Nov 2004, *C.A. Cruz-Espinosa & G. Juárez-García 1945* (MEXU). **Mun. San Felipe Jalapa de Díaz**, 500 m, 28 Jun 2008, *J.A. Pérez de la Rosa et al. 1966* (IBUG). **Mun. San Pedro Teutila**, 458 m, 23 Sep 2020, *F. Nicolalde-Morejón et al. 3323–3333* (CIB); 500 m, 23 Sep 2020, *F. Nicolalde-Morejón et al. 3334–3337* (CIB); 615 m, 28 Sep 2020, *F. Nicolalde-Morejón et al. 3415–3419* (CIB); 708 m, 10 Jun 2004, *G. Juárez-García 425* (MEXU); 458 m, 23 Sep 2020, *L. Martínez-Domínguez et al. 1946–1950* (CIB), *1951* (CIB, MEXU), *1952–1957* (CIB); 500 m, 23 Sep 2020, *L. Martínez-Domínguez et al. 1958–1960* (CIB, MEXU), *1961* (CIB); 615 m, 28 Sep 2020, *L. Martínez-Domínguez et al. 2050, 2052* (CIB, MEXU), *2051, 2053* (CIB); 539 m, 6 Apr 2021, *L. Martínez-Domínguez et al. 2141, 2142, 2144* (CIB), *2143* (CIB, MEXU, NY). **Mun. Santa María Tlalixtac**, 675 m, 25 Nov 2004, *G. Juárez-García & C.A. Cruz-Espinosa 868* (MEXU).

#### ﻿3. Ceratozamia becerrae

Taxon classificationPlantaeCycadalesZamiaceae

Pérez-Farr., Vovides & Schutzman, Bot. J. Linn. Soc. 146: 124. 2004

960BDC31-034E-5808-8BBC-A73C600FFCAC

Fig. 15C

##### Type.

Mexico. Tabasco: Mun. Teapa, hill Madrigal, Aug 2003, *A.P. Vovides 1458* (holotype: XAL [XAL0148355]).

##### Description.

***Stem*** 13–38 cm long, 9–15 cm in diameter, epigeous, semi-hypogeous, erect. ***Cataphylls*** 2.6–5.7 × 1.3–4.5 cm wide at the base, persistent, triangular, reddish brown, densely brownish tomentose at emergence, glabrous at apex when mature, apex acuminate. ***Leaves*** 2–12 (17), 49–210 cm long, descending, light green, glaucous at emergence with whitish gray trichomes, glabrous at maturity. ***Petiole*** 35–111.5 cm long, terete, linear, pink at emergence, yellowish green when mature; unarmed to armed with 3–27 thin prickles, 0.02–0.13 cm long. ***Rachis*** 30–105 cm long, terete, linear, pink at emergence, yellowish green at mature leaves, generally unarmed. ***Leaflets*** 6–15 pairs, opposite to subopposite, insertion in one plane, oblong to oblanceolate, longitudinally curved abaxially to planar, generally basally falcate, coriaceous, flat, green with adaxial and abaxial sides glaucous or glabrous, distal end with entire margins, acuminate to acute, and asymmetrical (rarely symmetrical in apical leaflets) at the apex, attenuate at base, with conspicuous and light-green veins; median leaflets 16–30 × 4.5–8.8 cm, 5.5–11.1 cm between the leaflets; articulations 0.5–1.5 cm wide, green and yellowish. ***Pollen strobili*** solitary, 10.8–20 cm long, 2.8–4.0 cm in diameter, cylindrical, erect, green with blackish trichomes at emergence, yellow-cream with blackish trichomes at maturity; peduncle 5–10 cm long, 1.5–2.0 cm in diameter, tomentose, light brown; microsporophylls 0.9–1.37 × 0.7–0.9 cm, discoid with a non-recurved distal face and a deeply lobate fertile portion, infertile portion 0.35–0.39 cm long and linear with straight horns and 0.30–0.45 cm long, 0.70–0.80 cm and an acute angle between the horns. ***Ovulate strobili*** 15–23 cm long, 7.5–8.5 cm in diameter, solitary, cylindrical, erect, green with reddish brown trichomes at emergence, green with brown to blackish trichomes at maturity, acute apex; peduncle 5–12 cm long, 1.6–2 cm in diameter, pendulous and erect, tomentose, light brown; megasporophylls 43–56, 6–8 orthostichies with 5–7 sporophylls per orthostichy, 1.7–2.0 × 3.5–4.0 cm, with a prominent distal face, horns straight and 0.70–0.87 cm long, 0.92–1.50 cm between horns with a right angle between the horns. ***Seeds*** 1.5–2.4 cm long, 1.2–2.0 cm in diameter, ovate, sarcotesta whitish pink at emergence, light brown at maturity.

##### Distribution and habitat.

*Ceratozamiabecerrae* is endemic to the mountains of Sierra Madrigal in southern Tabasco and Chiapas States, Mexico (Fig. 14C), where it occurs on karstic outcrops in evergreen tropical forest and oak forest from 100–800 m.

##### Etymology.

The specific epithet was established in honor of Professor Marco E. Becerra for his relevant contributions in ethnology, archaeology and floristic research in Tabasco (Vovides et al. 2004).

##### Common names.

None recorded.

##### Uses.

None recorded.

##### Conservation status.

(IUCN 2021). Endangered; A2ac+4ac; B2ab(i,ii,iv); C1.

##### Discussion.

*Ceratozamiabecerrae* has oblong and coriaceous leaflets and leaves that have a few thin and short prickles. This species belongs to a cryptic taxonomic group with *C.zoquorum* and *C.santillanii* (c.f. Martínez-Domínguez et al. 2017c; Vovides et al. 2020) and is morphologically similar in both vegetative and reproductive characters to *C.zoquorum*. This species differs from *C.santillanii* by its peduncle of ovulate strobili more than 3 cm long, but lacks morphological diagnoses with *C.zoquorum*.

##### Specimens examined.

Mexico. **Chiapas: Mun. Amatán**, 300 m, *J.M. Lázaro Z. 376* (CHIP). **Tabasco: Mun. Tacotalpa**, 260 m, 12 Apr 2014, *F. Nicolalde-Morejón et al. 1999, 2000* (CIB); 260 m, 12 Apr 2014, *L. Martínez-Domínguez et al. 108–127* (CIB); 19 Jan 2001, *S. Avendaño R. 5214b* (XAL). **Mun. Teapa**, 100 m, 29 Jan 1985, *B.M. Schutzman 645, 648–650* (XAL); 204 m, 22 Feb 2014, *F. Nicolalde-Morejón et al. 1968, 1969* (CIB); 800 m, 11 Jun 1989, *J.A. Alejandre Rosas 494* (CIB); 204 m, 22 Feb 2014, *L. Martínez-Domínguez et al. 35–40* (CIB); 400 m, 16 Apr 1996, *M.A. Pérez-Farrera 901* (CHIP, HEM, MEXU); 800 m, 7 Apr 1914, *M.E. Becerra s/n* (MEXU).

#### ﻿4. Ceratozamia brevifrons

Taxon classificationPlantaeCycadalesZamiaceae

Miq., Tijdschr. Wis-en natuurk Wet. 1: 41. 1847

8D2FCB5E-FA52-5DE3-AD0D-AEBDF3537D6E

**Figure 15.**
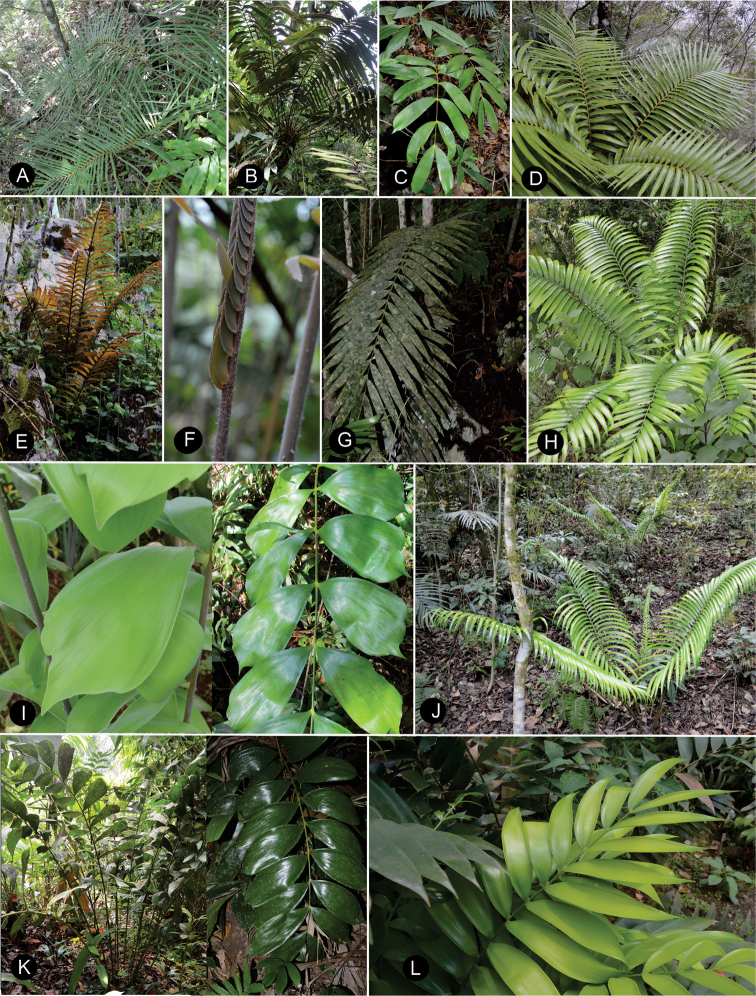
Plants of *Ceratozamia* in habitat **A***C.alvarezii***B***C.aurantiaca***C***C.becerrae***D***C.brevifrons***E***C.chamberlainii***F***C.chimalapensis***G***C.decumbens***H***C.delucana***I***C.euryphyllidia***J***C.fuscoviridis***K***C.hondurensis***L***C.huastecorum*.

Figs 3B , 12C , 14D , 15D

##### Type.

Mexico. Veracruz: Mun. Alto Lucero de Gutiérrez Barrios, Apr 2005, *S. Avendaño R. 5699* (neotype, designated by Vovides et al. 2012, pg. 38: XAL! [XAL0132508]).

##### Description.

***Stem*** 20–70 cm long, 15–40 cm in diameter, epigeous, erect. ***Cataphylls*** 2–5 × 1.5–4 cm wide at the base, persistent, triangular, reddish brown, densely brownish tomentose at emergence, glabrous at maturity with an acuminate apex. ***Leaves*** 6–36, 58–173.5 cm long, descending, yellowish green at emergence with brown trichomes, glabrous at maturity. ***Petiole*** 20–56 cm long, terete, linear, green in mature leaves; with 40–90 robust prickles, 0.24–50 cm long. ***Rachis*** 35–125.5 cm long, terete, linear, green in mature leaves, with prickles. ***Leaflets*** 13–38 pairs, opposite to subopposite, insertion keeled, sometimes imbricate, lanceolate, abaxially curved, basally falcate, coriaceous, flat, light green with adaxial and abaxial sides glabrous, distal end with entire margins, acuminate and symmetrical to asymmetrical at the apex, attenuate at base, with conspicuous and green-light veins; median leaflets 15.5–41 × 2–4.1 cm, 0.5–3.2 cm between leaflets; articulations 0.6–1.7 cm wide, yellow. ***Pollen strobili*** 18–31 cm long, 4–7 cm in diameter, solitary, cylindrical, erect, greenish yellow at emergence, greenish yellow with brown to blackish trichomes at maturity; peduncle 4–10.3 cm long, 1.7–2.2 cm in diameter, reddish brown to light-brown pubescent; microsporophylls 1.55–2.3 × 0.80–1.5 cm, obconic with a non-recurved distal and a lobate fertile portion, infertile portion 0.50–0.70 cm long and rounded with straight horns 0.24–0.40 cm long, 0.50–1.02 cm and an acute angle between the horns. ***Ovulate strobili*** 25–33 cm long, 9.8–12.5 cm in diameter, solitary, cylindrical, erect, green with blackish trichomes at emergence, greenish yellow with brown to blackish trichomes at maturity and with an acuminate and apiculate apex; peduncle 6–14 cm long, 2.0–2.4 cm in diameter, pendulous and erect, brown to reddish brown pubescent; megasporophylls 80–224, 8–16 orthostichies with 10–15 sporophylls per orthostichy, 1.5–2.8 × 2.5–3.2 cm, with a prominent distal face, horns straight and robust and 0.75–0.95 cm long, 1.15–1.70 cm between horns with an acute angle between the horns. ***Seeds*** 2.0–3.0 cm long, 1.3–2.0 cm in diameter, ovate, sarcotesta whitish yellow to yellow when immature, light brown at maturity.

##### Distribution and habitat.

*Ceratozamiabrevifrons* is known only from Sierra de Chiconquiaco in Veracruz State, Mexico (Fig. 14D) where it occurs in the transition zone between cloud forest and oak forest at 450 to 1,370 m.

##### Etymology.

The epithet is derived from its relatively short leaves.

##### Common names.

Mexico. Veracruz: Palma (J. Rees 1636); palmilla (A.P. Vovides et al. 682).

##### Uses.

None recorded.

##### Conservation status.

The area of distribution of *Ceratozamiabrevifrons* is small and populations are close. Martínez-Domínguez et al. (2021) using ecological niche model estimated approximately 817 km2 of potential distribution with 558 km2 of transformed habitat. In addition, this species is not in a protected area. All data suggest that *C.brevifrons* could be assigned as “Endangered (EN)”.

##### Discussion.

*Ceratozamiabrevifrons* is easily distinguished from its congeners by having adaxially keeled and coriaceous leaflets, petioles armed with abundant short and robust prickles and greenish yellow ovulate strobili with brown to blackish brown trichomes at maturity.

##### Specimens examined.

Mexico. **Veracruz**: **Mun. Alto Lucero de Gutiérrez Barrios**, 24 Aug 1976, *A.P. Vovides 119* (XAL); 8 Jan 2009, *D. Jimeno-Sevilla 694* (XAL); 1,052 m, 12 Jan 2013, *F. Nicolalde-Morejón et al. 1711*–*1731* (CIB); 1,052 m, 22 Aug 2014, *F. Nicolalde-Morejón & L. Martínez-Domínguez 2027*–*2046* (CIB); 1,250 m, 6 Apr 1981, *G. Castillo-Campos 1297* (XAL); 700 m, 3 Dec 1974, *J. Rees 1636* (MO, XAL), *1641, 1642*, 850 m, 21 Sep 1976, *1675* (XAL); 1,052 m, 21 Jun 2014, *L. Martínez-Domínguez & F. Nicolalde-Morejón 130*–*133* (CIB); 842 m, 6 Feb 2015, *L. Martínez-Domínguez & F. Nicolalde-Morejón 216*–226 (CIB); 1,052 m, 22 Mar 2015, *L. Martínez-Domínguez & F. Nicolalde-Morejón 298*–*309* (CIB); 450 m, 14 Jul 1995, *M. Vázquez-Torres 4790* (CIB); 24 Jun 2010, *M. Vázquez-Torres et al. 9186* (CIB); 850 m, 10 Jan 2001, *T.W. Walters 2001-02-A, B* (XAL). **Mun. Chiconquiaco**, 1,268 m, 10 April. 2015, *F. Nicolalde-Morejón & L. Martínez-Domínguez 2237*–*2241* (CIB); 1,340 m, 2 May 2019, *F. Nicolalde-Morejón et al. 3138–3147* (CIB); 1,268 m, 10 Apr 2015, *L. Martínez-Domíguez & F. Nicolalde-Morejón 556*–*560* (CIB); 1,340 m, 2 May 2019, *L. Martínez-Domíguez et al. 1729–1738* (CIB). **Mun. Colipa**, *Marts 1841* (MO). **Mun. Juchique de Ferrer**, 850 m, 30 Aug 1981, *A.P Vovides 682* (XAL); 1,250 m, 6 May 1981, *G. Castillo-Campos 1710, 1763, 1768* (XAL); 1,300 m, 7 May 1981, *G. Castillo-Campos 1815, 1824, 1981* (XAL); 1,370 m, 24 Jul 2008, *M. Vazquez-Torres 8633* (CIB). **Mun. Vega de Alatorre**, 650 m, 21 Jul 1981, *B. Guerrero & J.I. Calzada 1826* (XAL); 550 m, 21 Jul 1981, *G. Castillo-Campos 2033* (XAL).

#### ﻿5. Ceratozamia chamberlainii

Taxon classificationPlantaeCycadalesZamiaceae

Mart.-Domínguez, Nic.-Mor. & D.W.Stev., Phytotaxa 317(1): 22. 2017

26260370-1959-5B50-B5DF-F05114953E32

Figs 3C , 15E

##### Type.

Mexico. San Luis Potosí: Mun. Xilitla, 1,044 m, 20 Mar 2016, *L. Martínez-Domínguez et al. 933* ♀ (holotype: CIB! [acc. # 17766UV]; isotypes: MEXU! [MEXU1492226, MEXU1492227, MEXU14922278], NY!).

##### Description.

***Stem*** 20–60 cm long, 15–30 cm in diameter, epigeous, erect and decumbent. ***Cataphylls*** 2.5–5.5 × 1.5–3.5 cm wide at the base, persistent, narrowly triangular, reddish brown, densely brownish tomentose at emergence, glabrous at maturity, apex acuminate. ***Leaves*** (5)10–40, 100–207 cm long, descending, reddish brown at emergence with whitish gray trichomes, glabrous at maturity. ***Petiole*** 30–69 cm long, terete, linear, blackish brown in mature leaves; with 9–30 robust prickles, 0.03–0.30 cm long. ***Rachis*** 65–144 cm long, terete, linear, reddish brown in mature leaves, with prickles. ***Leaflets*** 20–42 pairs, opposite to subopposite, insertion in one plane, oblong, longitudinally curved abaxially to planar, not basally falcate, coriaceous, flat, dark green with adaxial and abaxial sides glabrous, distal end with entire margins, acuminate and asymmetric at the apex, attenuate at base, with conspicuous and reddish brown veins; median leaflets 20–37 × 2.3–4 cm, 1.6–3.5 cm between leaflets; articulations 0.5–1.3 cm wide, generally reddish brown. ***Pollen strobili*** 20–31 cm long, 4.5–6 cm in diameter, generally solitary (1–2), cylindrical, erect, greenish brown at emergence with reddish trichomes becoming greenish with reddish brown trichomes at maturity; peduncle 5.2–8.1 cm long, 1.6–2.3 cm in diameter, reddish brown pubescent; microsporophylls 1.6–2.3 × 0.9–1.5 cm, obconic with a non-recurved distal face and a deeply lobate fertile portion, infertile portion 0.44–0.55 cm long and linear with curved horns 0.25–0.40 cm long, 0.40–0.80 cm and an acute angle between the horns. ***Ovulate strobili*** 25.5–30 cm long, 7.5–10.5 cm in diameter, cylindrical, erect, greyish green with reddish brown trichomes at emergence and becoming light grayish brown with reddish brown trichomes at maturity with an acuminate apex; peduncle 4.5–11.5 cm long, 1.3–2.5 cm in diameter, erect, tomentose, brown to reddish brown; megasporophylls 49–180, 7–12 orthostichies with 7–15 sporophylls per orthostichy, 2.0–3.0 × 2.3–4.0 cm, with a prominent distal face, horns straight and 0.40–0.80 cm long, 2.0–2.70 cm between horns with an acute angle between the horns. ***Seeds*** 2.2–3.5 (4) cm long, 0.6–1.7 cm in diameter, ovate, sarcotesta whitish red to pink when immature, light brown at maturity.

##### Distribution and habitat.

**Figure 16.**
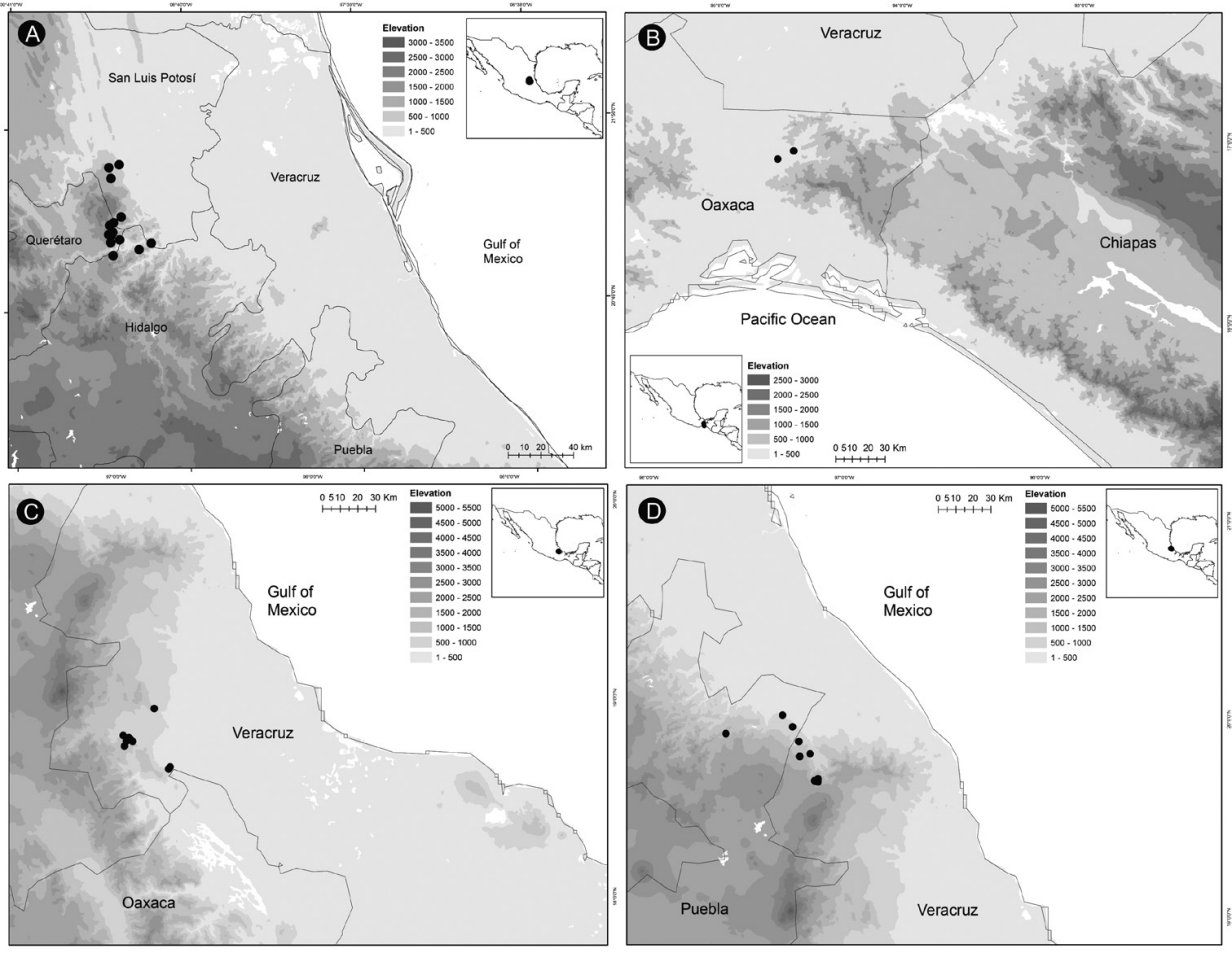
Distribution of *Ceratozamia* species **A***C.chamberlainii***B***C.chimalapensis***C***C.decumbens***D***C.delucana*.

*Ceratozamiachamberlainii* is distributed in the northern montane region of Carso Huasteco in San Luis Potosí, Querétaro and Hidalgo States, Mexico (Fig. 16A), where it occurs in cloud forest and pine-oak forest on rocky outcrops between 900–1,200 m.

##### Etymology.

The specific epithet is in honor of Charles Joseph Chamberlain in recognition of his remarkable contributions to knowledge of the biology of the cycads and his fieldwork on Mexican Cycads (Martínez-Domínguez et al. 2017a).

##### Common names.

Mexico. San Luis Potosí: Chamal (A.P. Vovides 1288), chamalillo (H. Puig 3979); Hidalgo: teocintle (A. Vite-Reyes et al. 23).

##### Uses.

None recorded.

##### Preliminary conservation status.

According to the IUCN criteria, the data suggest “Endangered (EN)”. *Ceratozamiachamberlainii* is included in Biosphere Reserve Sierra Gorda and have 9 populations recorded by us with several adult plants, juveniles and seedlings.

##### Discussion.

*Ceratozamiachamberlainii* is close geographically and morphologically to *C.fuscoviridis* and *C.latifolia*; however, it differs by its petioles and rachis bearing abundant and short prickles, and its oblong and coriaceous leaflets with conspicuous, reddish brown veins. In addition, the leaves are reddish brown at emergence and at maturity have this color in the leaflet articulations and at the base of leaflets in contrast to the yellowish to greenish articulations in *C.fuscoviridis* and *C.latifolia*.

##### Specimens examined.

Mexico. **Hidalgo: Mun. Chapulhuacán**, 1,500 m, 21 Feb 1998, *Alcántara-Ayala 3650* (FCME); 8 Jan 2009, *A. Vite-Reyes et al. 23* (XAL); 1,157 m, 30 Mar 2015, *F. Nicolalde-Morejón et al. 2200*–*2203* (CIB); 20 Sep 1964, *L. González-Quintero 4634* (ENCB); 1,157 m, 30 Mar 2015, *L. Martínez-Domínguez et al. 429*–*454* (CIB). **Mun. La Misión**, 1,120 a 1,400 m, 5 Oct 2007, *A. Castro-Castro et al*. *1017* (IBUG, XAL); 7 Jan 2009, *A. Vite-Reyes et al. 20* (XAL). **Querétaro**: **Mun. Landa de Matamoros**, 1,050 m, 28 May 1999, *A.P. Vovides 1288, 1289, 1290, 2000* (XAL), *1291* (XAL, MEXU); 2 Apr 1991, *E. Carranza 6333* (IEB); 940 m, 2 Apr 1991, *3119* (MEXU); 1,335 m, 30 Mar 2015, *F. Nicolalde-Morejón et al. 2192*–*2197* (CIB); 1,145 m, 30 Mar 2015, *F. Nicolalde-Morejón et al. 2198, 2199* (CIB); 1,335 m, 30 Mar 2015, *L. Martínez-Domínguez et al. 379* (CIB, MEXU), 380–*407* (CIB); 1,145 m, 30 Mar 2015, *L. Martínez-Domínguez et al. 408–428* (CIB); 1,070 m, 9 May 1989, *R. Hiram 647* (XAL). **San Luis Potosí**: **Mun. Aquismón**, 600 m, 10 Feb 1969, *H. Puig 3979* (ENCB, P); 27 May 1979, *J.A. Alcorn 3093* (MEXU); 1,125 m, 9 Jun 2015, *T. Diego-Vargas & M. Bonta 35* (XAL). **Mun. Tamazunchale**, Jul 1952, *A. Wilson 273, 274* (US); Jul 1937, *C.L. Lundell & A.A. Lundell 7235* (CIB, IEB); 600–900 m, 29 Jun 1959, *J. Rzedowski 11087* (ENCB). **Mun. Xilitla**, 1,044 m, 20 Mar 2016, *F. Nicolalde-Morejón et al. 2407*–*2420* (CIB); 1,044 m, 20 Mar 2016, *L. Martínez-Domínguez et al. 924*–*932, 934*–*937* (CIB), *938* (CIB, MEXU), *939* (CIB); 1,948 m, 12 Jan 2001, *T.W. Walters, TW-2001-04-A,B* (XAL), *TW-.2001-04-C* (MEXU).

#### ﻿6. Ceratozamia chimalapensis

Taxon classificationPlantaeCycadalesZamiaceae

Pérez-Farr. & Vovides, Bot. J. Linn. Soc. 157: 169. 2008

F5DCCDFE-D990-51DA-B219-ACF618415FE8

Fig. 15F

##### Type.

Mexico. Oaxaca: Chimalapa, 21 Jan 2002, *M.A. Pérez-Farrera 2622* ♀ (holotype: HEM [n.v.]; isotypes: XAL [XAL0146074], MEXU [n.v.], MO [n.v.]).

##### Description.

***Stem*** 20–60 cm long, 15–30 cm in diameter, epigeous, erect and decumbent. ***Cataphylls*** 4–7.5 × 2.5–4.3 cm wide at the base, persistent, triangular, reddish brown, scarce brown tomentose at emergence, glabrous at maturity, apex acuminate. ***Leaves*** 7–24, 66–250 cm long, ascending, brown at emergence, glabrous at maturity. ***Petiole*** 40–70 cm long, terete, linear, green in mature leaves; with 20–30 thin prickles, 0.05–0.30 cm long. ***Rachis*** 60–150 cm long, terete, linear, green in mature leaves, with prickles. ***Leaflets*** 36–70 pairs, opposite to subopposite, insertion in one plane, lanceolate, longitudinally curved abaxially to planar, not basally falcate, papyraceous, flat, light green with adaxial and abaxial sides glabrous, distal end with entire margins, acuminate and symmetrical at the apex, attenuate at base, with conspicuous and green-light veins; median leaflets 27–46 × 1.2–1.8 cm, 0.8–1.3 cm between leaflets; articulations 0.5–0.9 cm wide, brown to yellowish brown. ***Pollen strobili*** 25–40 cm long, 3–5 cm in diameter, solitary, cylindrical, erect, greenish yellow at emergence with few brown trichomes, greenish to cream at maturity; peduncle 5–11 cm long, 1.5–3 cm in diameter, reddish brown pubescent; microsporophylls 1.3–2 × 0.6–1 cm, elliptic with a non-recurved distal face and a lobate to slightly lobate fertile portion, infertile portion 0.7–1.1 cm long and linear with curved horns 0.30–0.43 cm long, 0.8–1.1 cm and an acute angle between the horns. ***Ovulate strobili*** 35–40 cm long, 7.3–10.6 cm in diameter, solitary, cylindrical, erect, green with blackish trichomes at emergence, green with blackish trichomes at maturity, acuminate apex; peduncle 7–11 cm long, 1.7–2 cm in diameter, erect, brown to reddish brown pubescent; megasporophylls 99–192, 11–12 orthostichies with 9–16 sporophylls per orthostichy, 4–5.9 × 1.8–2.5 cm, with a prominent distal face, horns straight and thin and 0.78–0.98 cm long, 0.98–2.0 cm between horns with an acute angle between the horns. ***Seeds*** 2.0–3.0 cm long, 1.4–1.9 cm in diameter, ovate, sarcotesta whitish yellow to yellow when immature, light brown at maturity.

##### Distribution and habitat.

*Ceratozamiachimalapensis* is endemic to Oaxaca State (Mexico) in the Sierra Atravesada mountain range at 290–1,000 m (Fig. 16B) in oak forest on clay soils.

##### Etymology.

The specific epithet is in honor of the Chimalapa region renowned for its biological richness, particularly its floristic diversity.

##### Common names.

Mexico. Oaxaca: Mazacopa (Vovides et al. 2008).

##### Uses.

The sarcotesta of seeds is used as food; the ground seed is used as a rodenticide and with honey added, used as an insecticide (Vovides et al. 2008).

##### Preliminary conservation status.

*Ceratozamiachimalapensis* is only known from a narrow area with several individuals. Data are insufficient to propose a conservation status at this time.

##### Discussion.

*Ceratozamiachimalapensis* is similar to *C.mirandae* and *C.alvarezii* in leaf morphology; however, there are differences in reproductive structures; the ovulate strobilus of *C.chimalapensis* is longer than in these species. The ovulate strobili have between 11–12 orthostichies with 9–16 sporophylls per orthostichy, and more than 90 seeds per strobilus. The seeds are ovate, whereas in the other two species, they are spherical.

##### Specimens examined.

Mexico. **Oaxaca: Mun. Santa María Chimalapa**, 290 m, 11 Jun 1995, *R. García S. 319* (SERO, XAL). **Mun. Santiago Niltepec**, 1000 m, 3 Apr 1946, *E. Hernández Xolocotzi & A.J. Sharp 1277* (MEXU).

#### ﻿7. Ceratozamia decumbens

Taxon classificationPlantaeCycadalesZamiaceae

Vovides, Avendaño, Pérez-Farr. & Gonz.-Astorga, Novon 18 (1): 110. 2008

3E10BF94-F815-5980-9A07-8DF5A3A280B1

Fig. 15G

##### Type.

Mexico. Veracruz: Naranjal, Near Cueva de Los Muñecos, 700 m, 8 Apr 2005, *S. Avendaño & G. Alducin 5706* (holotype: XAL! [XAL0005416, XAL0005418]; isotypes: HEM [n.v.], MO!).

##### Description.

***Stem*** 10–40 cm long, 10–25 cm in diameter, epigeous, erect and decumbent. ***Cataphylls*** 1.5–3 × 2–4.2 cm wide at the base, persistent, triangular, densely tomentose at emergence, reddish brown and partially tomentose at maturity, apex acuminate. ***Leaves*** 2–7, 80–190 cm long, descending, reddish brown at emergence with whitish gray trichomes, glabrous at maturity. ***Petiole*** 40–100 cm long, terete, linear, greenish brown in mature leaves; with 2–22 (28) thin prickles, 0.02–0.23 cm long. ***Rachis*** 40–123 cm long, terete, linear, brown and green in mature leaves, with prickles and occasionally unarmed. ***Leaflets*** 8–24 pairs, opposite to subopposite, insertion in one plane, oblong, in general longitudinally planar, not basally falcate, coriaceous, flat, green, adaxial and abaxial sides glabrous, distal end with entire margins, acuminate and symmetrical to asymmetrical at the apex, attenuate at base, with conspicuous and light-green veins; median leaflets 23–47.5 × 2.8–5 cm, 2.6–6.5 cm between leaflets; with articulations 0.7–1.2 cm wide, brown. ***Pollen strobili*** 20–23 cm long, 3.8–4.5 cm in diameter, solitary, cylindrical, erect, greenish yellow at emergence with reddish brown trichomes at maturity; peduncle 8–10.8 cm long, 1.2–1.6 cm in diameter, reddish brown to brown pubescent; microsporophylls 1–1.9 × 1–1.4 cm, obconic with a non-recurved distal face and lobate fertile portion, infertile portion 0.59–0.65 cm long and linear with straight horns 0.27–0.33 cm long, 0.67–0.80 cm and an acute angle between the horns. ***Ovulate strobili*** 9–11 cm long, 7–8 cm in diameter, solitary, cylindrical, erect, wine at emergence, wine with blackish brown trichomes at maturity, with an acute apex; peduncle 3–4 cm long, 1–1.2 cm in diameter, erect, brown pubescent; megasporophylls 18–49, 6–7 orthostichies with 3–7 sporophylls per orthostichy, 2.3–2.5 × 2–3 cm, with truncate distal face, horns straight and thin and 0.45–0.58 cm long, 0.99–1.48 cm between horns with a right angle between the horns. ***Seeds*** 1.2–2 cm long, 1.2–1.5 cm in diameter, ovate, sarcotesta whitish red when immature, light brown at maturity.

##### Distribution and habitat.

*Ceratozamiadecumbens* is endemic to a small central mountain range in Veracruz State, Mexico (Fig. 16C) growing on karstic rocks in mountain tropical forest and cloud forest at 450–1,100 m.

##### Etymology.

The epithet alludes to the decumbent nature of trunks in older mature plants.

##### Common names.

None recorded.

##### Uses.

None recorded.

##### Preliminary conservation status.

*Ceratozamiadecumbens* has not been listed in the IUCN Red List of Threatened Species (https://www.iucnredlist.org/). Its distribution area has been severely affected by anthropogenic land-use changes (Martínez-Domínguez et al. 2021). However, known populations have between 100 to 150 adult plants with juvenile and seedlings. All data suggest that the conservation status should be “Endangered (EN)”.

##### Discussion.

*Ceratozamiadecumbens* is distinguished from its most similar species (*C.mexicana* and *C.morettii*) by the ovulate strobili which are are wine red with blackish brown trichomes at maturity and an acute apex, whereas in *C.mexicana* they are green with blackish brown and gray trichomes with an acuminate apex, and *C.morettii* are green with with blackish brown trichomes with an apiculate apex. In terms of vegetative morphology, it differs from these species by its reddish brown leaves with whitish gray trichomes at emergence.

##### Specimens examined.

Mexico. **Veracruz: Mun. Atoyac**, 900 m, 28 Jan 1986, *R. Acevedo R. 728* (XAL). **Mun. Coetzala**, 650 m, 30 Nov 2001, *A. Rincón G. 2798* (MEXU, XAL); 870 m, 15 Jul 2015, *L. Martínez-Domínguez et al. 655*–*683* (CIB). **Mun. Córdoba**, 1,100 m, 10 Jun 1985, *A. Espíritu & J.L. Martínez 94* (XAL). **Mun. Ixtaczoquitlán**, 1,090 m, 25 May 1985, *A. Pérez P. 282* (XAL) **. Mun. Naranjal**, 11 Sep 1982, *A.P. Vovides 751* (XAL); 10 Oct 1993, *Brigada T. Walters s/n* (XAL); 11 Sep 1982, *J. Rees 1690* (XAL); 10 Oct 1993, *T.W. Walters 41277, 41308, 41397* (XAL). **Mun. Tequila**, 445 m, 28 Oct 2007, *J.E. Rivera Hdez. & A. Vergara V. 4195* (MEXU, XAL); 959 m, 15 Jul 2015, *F. Nicolalde-Morejón et al. 2259, 2260* (CIB); 959 m, 15 Jul 2015, *L. Martínez-Domínguez et al. 684*–*703* (CIB). **Mun. Tezonapa**, 1 Dec 1995, *M.A. García B. 980* (XAL); 475 m, 24 Jun 1986, *R. Robles G. 882* (XAL). **Mun. Zongolica**, 11 Mar 2011, *L. Hermann Bojórquez G. et al. 2337* (CIB).

#### ﻿8. Ceratozamia delucana

Taxon classificationPlantaeCycadalesZamiaceae

Vázq.Torres, A.Moretti & Carv.-Hern., Delpinoa 50–51: 129. 2013 (“2008-2009”)

DAFB9C6F-463C-540F-9EF5-31F67394F01C

Figs 2A, B , 10A, B , 15H

##### Type.

Mexico. Veracruz: Mun. Atzalan, road Atzalan- Tlapacoyan, 3 km NE from Atzalan, 20 Jan 2012, *M. Vázquez-Torres & C. Carvajal-Hernández 10200* ♀ (holotype: CIB! [acc. # 13915UV]; isotypes: XAL [n.v.], XALU!).

##### Description.

***Stem*** 20–90 cm long, 25–40 cm in diameter, epigeous, erect and decumbent. ***Cataphylls*** 2–5.5 × 2.5–4.5 cm wide at the base, persistent, narrowly triangular, reddish brown, densely tomentose at emergence, partially tomentose at maturity, apex acuminate. ***Leaves*** 10–100, 106–223 cm long, ascending, yellowish green at emergence with brown trichomes, glabrous at maturity. ***Petiole*** 30–87 cm long, terete, linear, light green at mature leaves; with 35–76 thin prickles, 0.21–0.76 cm long. ***Rachis*** 60–150 cm long, terete, linear, green in mature leaves, with prickles. ***Leaflets*** 20–43 pairs, opposite to subopposite, insertion in one plane, lanceolate and oblong, longitudinally curved abaxially to planar, basally falcate to non-basally falcate, papyraceous to coriaceous, flat, green, adaxial side glaucous and glabrous and abaxial side glaucous, distal end with entire margins, acuminate and symmetrical to asymmetrical at the apex, attenuate at base, with conspicuous and light-green veins; median leaflets 22–45 × 2.3–4.6 cm, 1.5–5 cm between leaflets; articulations 0.6–1.6 cm wide, green. ***Pollen strobili*** 24–32 cm long, 5.5–7.6 cm in diameter, solitary, cylindrical, erect, greenish yellow at emergence, greenish yellow with blackish trichomes at maturity; peduncle 3.5–12.5 cm long, 1.3–2 cm in diameter, tomentose, reddish brown to light-brown; microsporophylls 1.5–2.5 × 1.3–2 cm, discoid and obconic with a non-recurved distal face and a lobate fertile portion, infertile portion 0.30–0.45 cm long and orbicular with straight horns 0.15–0.25 cm long, 0.40–0.60 cm and a right angle between the horns. ***Ovulate strobili*** 17–40 cm long, 10–13.5 cm in diameter, solitary, cylindrical and globose, erect, dark green with blackish trichomes at emergence, green, generally glabrous at maturity, acute apex; peduncle 5.2–15 cm long, 1.8–2.2 cm in diameter, erect and pendulous, tomentose, brown to reddish brown; megasporophylls 48–266, 7–14 orthostichies with 6–19 sporophylls per orthostichy, 2.2–4.2 × 3.4–4.5 cm, with a truncate distal face, horns straight and 0.45–0.72 cm long, 1.05–1.67 cm between horns with a right angle between the horns. ***Seeds*** 2.0–3 cm long, 1.35–2.1 cm in diameter, ovate, sarcotesta whitish yellow to yellow when immature, light brown at maturity.

##### Distribution and habitat.

*Ceratozamiadelucana* occurs in Veracruz and Puebla States, Mexico (Fig. 16D) and grows on karstic rocks in evergreen tropical forest at 500–1,650 m.

##### Etymology.

The epithet is in honor of Dr. Paolo De Luca, Professor at University of Naples Federico II and a researcher into the biology of Mexican cycads (Vázquez-Torres et al. 2013).

##### Common names.

None recorded.

##### Uses.

None recorded.

##### Conservation status.

During the last 6 years, we have monitored the 6 populations for *Ceratozamiadelucana*, in which no loss of individuals and regeneration has been observed. These populations are periodically producing ovulate and pollen strobili. The main problem is the loss of habitat in the central area of Veracruz, where there are no protected natural areas (Martínez-Domínguez et al. 2021). These data suggest the category “Endangered (EN)” under (B1ab(iii)) criteria.

##### Discussion.

*Ceratozamiadelucana* is highly variable and in vegetative morphology similar to *C.morettii*. However, there are clear differences in their ovulate strobili. In *C.delucana*, ovulate strobili are green and generally without trichomes at maturity and have an acute apex, whereas in *C.morettii* they are green with blackish trichomes at maturity and have an apiculate apex. Additionally, *C.delucana* is a larger plant than *C.morettii*, with *C.delucana* having leaves up to 223 cm long with up to 43 pairs of leaflets and ovulate strobili 17–40 cm long.

##### Specimens examined.

Mexico. **Puebla: Mun. Hueytamalco**, 520 m, 25 Feb 2008, *G. Ibarra Manríquez et al. 5485* (MEXU, MO). **Mun. Xochitlán de Vicente Suárez**, 1,644 m, 9 Jun 2015, *F. Nicolalde-Morejón & L. Martínez-Domínguez 2244, 2245* (CIB); 850 m, 25 Apr 1991, *G. Villalobos & E. Guerrero C*. *325* (MEXU); 1,644 m, 9 Jun 2015, *L. Martínez-Domínguez & F. Nicolalde-Morejón 587*–*616* (CIB). **Veracruz: Mun. Atzalan**, 1,400 m, 20 Feb 2013, *F. Nicolalde-Morejón et al. 1740–1742* (CIB); 1,400 m, 16 Aug 2014, *F. Nicolalde-Morejón et al. 2125*–*2144* (CIB); 1,400 m, 13 Mar 2015, *F. Nicolalde-Morejón et al. 2168* (CIB); 1,400 m, 27 Oct 2008, *L. Lagunes-Galindo et al. 153* (CIB); 1396 m, 28 Oct 2008, *L. Lagunes-Galindo et al. 155* (CIB); 1,400 m, 16 Aug 2014, *L. Martínez-Domínguez et al. 163* (CIB); 863 m, 13 Mar 2015, *L. Martínez-Domínguez et al. 228*–*238* (CIB); 1,400 m, 13 Mar 2015, *L. Martínez-Domínguez et al. 239*–*248* (CIB). **Mun. Las Minas**, 2 Jun 1979, *A.P. Vovides 427* (XAL); 1,500 m, 22 Sep 1988, *C. Durán et al. 658* (XAL), *660* (MEXU, XAL); 1,500 m, 22 Sep 1988, *C. Durán E. 659* (MEXU, XAL); 1,586 m, 20 Feb 2013, *F. Nicolalde-Morejón et al. 1735*–*1739* (CIB); 1,621 m, 16 Aug 2014, *F. Nicolalde-Morejón & L. Martínez-Domínguez 2107*–*2124* (CIB); 1,621 m, 16 Aug 2014, *L. Martínez-Domínguez et al. 162* (CIB); 1,621 m, 18 Mar 2015, *L. Martínez-Domínguez et al. 249–260* (CIB); 1 Mar 2005, 1,430 m, *L.H. Bojórquez-Galván 1349* (CIB); 1,420 m, 16 Mar 2005, *L.H. Bojórquez-Galván 1374* (CIB); 1,470 m, 28 Apr 2009, *M. Vázquez-Torres et al. 8972* (CIB). **Mun. Tlapacoyan**, 900 m, 10 Jun 1970, *Nevling & A. Gómez-Pompa 1083* (MEXU).

#### ﻿9. Ceratozamia euryphyllidia

Taxon classificationPlantaeCycadalesZamiaceae

Vázq.Torres, Sabato & D.W.Stev., Brittonia 38(1): 17. 1986

BA3DBA66-D678-5AAB-BC57-8BC7DE0749FD

Fig. 15I

##### Type.

Mexico. Veracruz: Mun. Minatitlán, 21 Jun 1984, *M. Vázquez-Torres 2842* ♀ (holotype: NY! [acc. # 1157–1166]; isotypes: CHAPA [n.v.], NY! [♂ acc. # 00001167– 00001173], XALU [n.v.]).

##### Description.

***Stem*** 25–50 cm long, 8–15.3 cm in diameter, semi-hypogeous, erect and decumbent. ***Cataphylls*** 4.5–7 × 4.2–7.5 cm wide at the base, persistent, triangular, reddish brown, densely tomentose at emergence, glabrous at maturity, apex acute. ***Leaves*** 2–22, 95–337.5 cm long, ascending, light green, glaucous at emergence, with whitish gray trichomes, glabrous at maturity. ***Petiole*** 40–193 cm long, terete, linear, green to yellowish (pink in new leaves); with 8–35 thin prickles, 0.13–0.50 cm long. ***Rachis*** 53–218.5 cm long, terete, linear, green to yellowish in mature leaves, with prickles. ***Leaflets*** 6–17 pairs, opposite to subopposite, insertion in one plane, broadly obovate, in general longitudinally planar, not basally falcate, membranaceous, flat, green with adaxial and abaxial sides glabrous, distal end with sinuate margins, acuminate and asymmetrical at the apex, attenuate at base, with prominent and light-green veins; median leaflets 19.9–35.7 × 8.5–17.6 cm, 9–22 cm between leaflets; articulations 0.9–1.4 cm wide, yellow and green. ***Pollen strobili*** 27–35 cm long, 3–4.3 cm in diameter, solitary, cylindrical, erect, greenish yellow with reddish trichomes at emergence, greenish with black trichomes at maturity; peduncle 6–12 cm long, 1.5–2 cm in diameter, tomentose, reddish brown to brown; microsporophylls 1.0–1.5 × 0.5–1.3 cm, discoid with a non-recurved distal face and a lobate fertile portion, infertile portion 0.37–0.45 cm long and orbicular and rounded with straight horns 0.35–0.50 cm long, 0.60–0.72 cm and an acute angle between the horns. ***Ovulate strobili*** 15–20 cm long, 5–6 cm in diameter, solitary, cylindrical, erect, yellowish green to green with abundant, deep red trichomes at emergence, greenish brown with abundant, dark reddish brown trichomes at maturity, acuminate apex; peduncle 5–11.5 cm long, 1–2.5 cm in diameter, erect, tomentose, light brown; megasporophylls 35–64, 1.5–3 × 1.2–1.8 cm, 7–8 orthostichies with 5–8 sporophylls per orthostichy, 2.0–4.0 × 2.8–3.4 cm wide, with a truncate distal face, horns straight and 0.60–0.80 cm long, 0.99–1.40 cm between horns with an acute angle between the horns. ***Seeds*** 2.3–2.5 cm long, 1.5–1.7 cm in diameter, ovate, sarcotesta whitish red when immature, light brown at maturity.

##### Distribution and habit.

**Figure 17.**
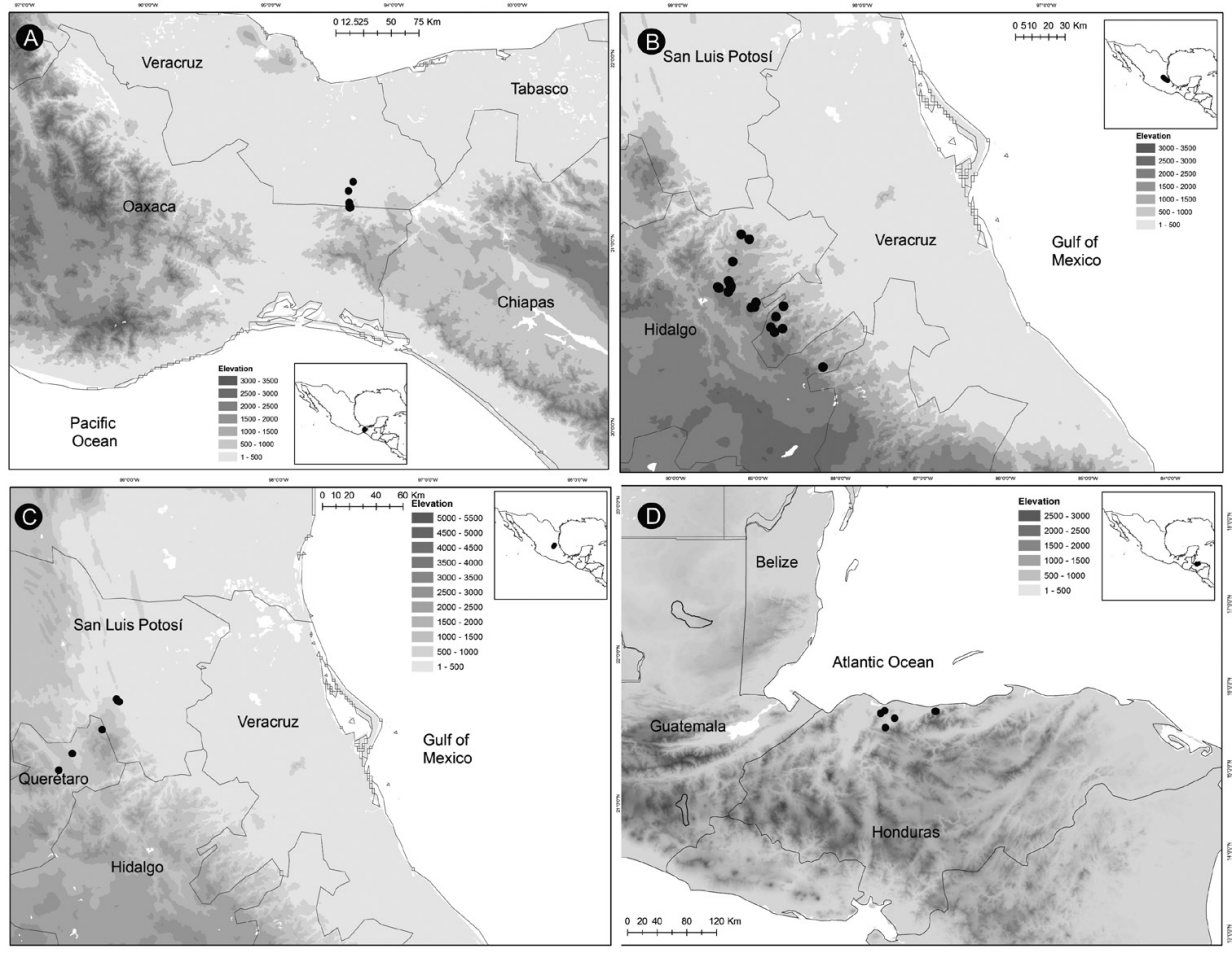
Distribution of *Ceratozamia* species **A***C.euryphyllidia***B***C.fuscoviridis***C***C.hildae***D***C.hondurensis*.

*Ceratozamiaeuryphyllidia* is endemic to the forest of Uxpanapa-Chimalapas in Oaxaca and Veracruz States, Mexico (Fig. 17A) where it inhabits evergreen tropical rain forest on clay soils at the top and sides of hills between 100 and 630 m elevation.

##### Etymology.

The specific epithet represents the very wide, diagnostic leaflets of this species. It comes from the Greek euryphyllos, which means, “wide leaflet”.

##### Common names.

None recorded.

##### Uses.

None recorded.

##### Conservation status.

(IUCN 2021). *Ceratozamiaeuryphyllidia* is listed as “Critically Endangered (CR)” under criteria B2ab(i,ii,iii,iv,v). We carried out a census in two populations for this species, in which we found between 20 to 60 adult plants. The population in Veracruz has reproductive plants, juvenile and seedlings; however, it is a small population and the surrounding area has been transformed to anthropogenic landscapes. We suggest that this status should be maintained.

##### Discussion.

*Ceratozamiaeuryphyllidia* is easily diagnosable from its congeners in Mexico by its broadly obovate, lustrous and membranaceous leaflets asymmetrical apex of leaflets. Also, the ovulate strobili are greenish brown with an acuminate apex, and its megasporophylls are green with abundant dark brown trichomes. This species is most similar to *C.hondurensis*, but it can be distinguished by its sinuate margins at distal end of leaflets, whereas in *C.hondurensis* they are entire.

##### Specimens examined.

Mexico. **Veracruz**: **Mun. Minatitlán**, 100 m, 24 Jul 1986, *A.P. Vovides & E.R. Acosta 1108* (MO, XAL); 21 Jun 1984, *M. Vázquez-Torres 1157* (NY). **Mun. Jesús Carranza**, 24 May 1982, *M. Vázquez-Torres 2532* (CIB, MEXU); 26 Mar 1982, *M. Vázquez-Torres et al. 2374* (CHAPA, MEXU, NY); 100 m, 5 Jun 1989, *M. Vázquez-Torres & J.P. Sclavo* 4037 (CIB); 18 Apr 1982, *M. Vázquez-Torres 2451* (CHAPA, MEXU, MO, NY); 120 m, 28 Jul 1983, *M. Vázquez-Torres 2614* (CIB, NY); 21 Jun 1984, *M. Vázquez-Torres 4126* (NY); 120 m, 28 Dec 1984, *T. Wenth et al. 4732* (CHAPA). **Mun. Uxpanapa**, 398 m, 18 Mar 2014, *F. Nicolalde-Morejón & L. Martínez-Domínguez 1984* (CIB); 19 Mar 2014, *L. Martínez-Domínguez & F. Nicolalde-Morejón* 71–77 (CIB). **Mun. Hidalgotitlán** (plant cultivated at JBC-INECOL), *V.E. Luna M. 1520* (XAL). **Oaxaca: Mun. Santa María Chimalapa**, 232 m, 13 May 1995, *E. Torres B. 687* (SERO, XAL); 398 m, 18 Mar 2014, *F. Nicolalde-Morejón & L. Martínez-Domínguez 1982, 1983* (CIB); 335 m, 5 May 1996, *J.E. Rivera H. & S. Escobedo 102* (XAL); 630 m, 16 May 1998, *J.E. Rivera H. et al. 700* (MEXU); 475 m, 21 Mar 1999, *J.E. Rivera H. et al. 1195* (SERO); 398 m, 18 Mar 2014, *L. Martínez-Domínguez & F. Nicolalde-Morejón 51–70* (CIB).

#### ﻿10. Ceratozamia fuscoviridis

Taxon classificationPlantaeCycadalesZamiaceae

W.Bull., Retail List. 154: 4. 1879

39A45C84-3C3E-50AC-BCF2-9799D3EB3B28

Figs 3A , 9C , 15J

Ceratozamia mexicana Brongn. f. fuscoviridis (W.Bull.) J.Schust., Pflanzenr. (Engler) Heft 99, 4 fam 1: 132. 1932, as C.mexicanavar.longifoliaf.fuscoviridis. Type: Based on Ceratozamiafuscoviridis W.Bull.

##### Type.

Cultivated at Glasnevin, Ireland “Hort. Bot. Glasnevin”, 21 Mar 1878 (accessioned 1881), *D. Moore s.n.* (neotype, designated by Calonje and Sennikov 2017, pg. 161: K! [K000501714, K000501712, K000501713]). Mexico. Hidalgo: Mun. Molango, km 3 carretera Molango-Xochicoatlán, 1,860 m, 31 Mar 2015, *L. Martínez-Domínguez et al. 493* ♀ (epitype, designated by Martínez-Domínguez et al. 2018a: 105: CIB! [acc. # 17465UV]; isoepitype: MEXU! [acc. # 1520508, 1520282, 1520519]).

##### Description.

***Stem*** 20–90 cm long, 25–40 cm in diameter, epigeous, erect and decumbent. ***Cataphylls*** 2–4.5 × 2–3.5 cm wide at the base, persistent, narrowly triangular, reddish brown, densely brownish tomentose at emergence, tomentose at maturity, apex acuminate. ***Leaves*** 10–70, 92–215 cm long, ascending, light green and dark brown at emergence, with whitish gray trichomes, glabrous at maturity. ***Petiole*** 40–95 cm long, terete, linear, dark green in mature leaves; with 14–60 (66) thin prickles, 0.09–0.33 cm long. ***Rachis*** 65–150 cm long, terete, linear, green in mature leaves, with prickles. ***Leaflets*** 28–67 pairs, opposite to subopposite, insertion in one plane, lanceolate, abaxially curved longitudinally, basally falcate, papyraceous, flat, green, with adaxial and abaxial sides glabrous, distal end with entire margins, acuminate and symmetrical at the apex, attenuate at base, with conspicuous and green-light brown veins; median leaflets 16.6–42 × 1.3–2.1 cm, 0.6–2 cm between leaflets; articulations 0.6–1.3 cm wide, green and brown. ***Pollen strobili*** 22–30 cm long, 5–8 cm in diameter, solitary, cylindrical, erect, brownish yellow at emergence, greenish brown with reddish brown trichomes at maturity; peduncle 5–14.5 cm long, 1.6–2.3 cm in diameter, tomentose, reddish brown to brown; microsporophylls 1.6–2.4 × 1.2–1.6 cm, obconic with a non-recurved distal face and a deeply lobate fertile portion, infertile portion 0.45–0.56 cm long and orbicular with straight horns 0.16–0.29 cm long, 0.53–0.80 cm and a right angle between the horns. ***Ovulate strobili*** 24–35 cm long, 8.5–15 cm in diameter, solitary, cylindrical, erect, green with brown trichomes at emergence, brown-green with dark brown trichomes at maturity, acuminate apex; peduncle 4–15.5 cm long, 1.8–2.5 cm in diameter, erect or pendulous, tomentose, brown to reddish brown; megasporophylls 49–195, 7–15 orthostichies with 7–15 sporophylls per orthostichy, 2.3–3.5 × 3.0–4.0 cm, with a truncate distal face, horns straight and 0.70–0.99 cm long, 1.7–2.1 cm between horns and an obtuse angle between the horns. ***Seeds*** 2–2.6 cm long, 1.5–2 cm in diameter, ovate, sarcotesta whitish yellow to yellow when immature, light brown at maturity.

##### Distribution and habitat.

*Ceratozamiafuscoviridis* is endemic to south to central Carso Huasteco in Mexico from Hidalgo to northwest of Veracruz States (Fig. 17B) in cloud forests between 1,300–1,900 m elevation.

##### Etymology.

The epithet refers to the dark-brown color of the leaf at emergence.

##### Common names.

Mexico. Veracruz: teocintle, teocintli and tepecintli (Bonta et al. 2019).

##### Uses.

This species has decorative uses associated with religious ceremonies and national holidays. The leaves are commonly used to make arches in the entrances of some Roman Catholic churches or decorate the walls during national holidays.

##### Conservation status.

(IUCN 2021). *Ceratozamiafuscoviridis* is listed as “Critically Endangered” under criteria B1ab(i,iii,iv,v). This species requires a reassessment of its conservation status because new populations have recently been recorded, including its current circumscription that includes populations from Veracruz State. Based on the number of populations, modelled potential geographical distribution from ecological niche and its occurrence in a Natural Protected Areas (Los Mármoles National Park), *C.fuscoviridis* could be listed as “Endangered (EN)” (Martínez-Domínguez et al. 2021).

##### Discussion.

*Ceratozamiafuscoviridis* is polymorphic within populations because individual plants may have either a light green or dark-brown leaf color at emergence. Individuals with dark-brown leaf at emergence also have a brownish abaxial side. This species is most geographically proximate to *C.chamberlainii*, but it can be distinguished by lanceolate and papyraceous leaflets, a petiole armed with long and thin prickles; and ovulate strobili that are brownish green with dark trichomes at maturity.

Osborne et al. (2006) intended to validate *Ceratozamiafuscoviridis* D.Moore, which was invalidly published in 1878. However, in 2017, Calonje & Sennikov published the correction to this name using a brief description by William Bull in horticultural catalogues. This work is the valid publication for the specie and the name “*C.fuscoviridis* D.Moore” is an isonym with no nomenclatural status.

##### Specimens examined.

Mexico. **Hidalgo: Mun. Eloxochitlán**, 18 Mar 1995, *I. Luna-Vega 54716* (FCME). **Mun. Metztitlán**, 30 Dec 1992, *J.L. López-García 449* (ENCB, IBUG, MEXU). **Mun. Molango de Escamilla**, 1,380 m, 29 May 1999, *A.P. Vovides 1298* (XAL); 1,400 m, 29 May 1999, *A.P. Vovides 1301* (XAL); 1,500 m, 24 Jul 2008, *A. Vite-Reyes et al. 6* (XAL); 1,860 m, 31 Mar 2015, *F. Nicolalde-Morejón et al. 2209*–*2211* (CIB); 1,860 m, 31 Mar 2015, *L. Martínez-Domínguez et al. 485*–492, 494–*514* (CIB); *T.W. Walters 2001-03-A* (XAL). **Mun. Tenango de Doria**, 1,700 m, 12 Mar 1993, *I. Luna-Vega 914* (FCME). **Mun. Tlanchinol**, 1,450 m, 25 Aug 1992, *I. Luna-Vega s/n* (FCME); 1,420 m, 7 Oct 1992, *I. Luna-Vega 625* (FCME, XAL); 25 Aug 1992, *I. Luna-Vega 789* (XAL); 1,312 m, 31 Mar 2015, *F. Nicolalde-Morejón et al. 2204–2208* (CIB); 1,312 m, 31 Mar 2015, *L. Martínez-Domínguez et al. 455–484* (CIB). **Mun. Zacualtipán de Ángeles**, 23 Jan, 1983, *J. Rees 389* (CHAPA, FCME, MEXU); 1,360 m, 4 Dec 1974, *J. Rees 1611* (CHIP, XAL); 4 Dec 1974, *J. Rees 6339* (IEB). **Veracruz: Mun. Huayacocotla**, 1,913 m, 23 Feb 2005, *D. Saavedra Millán 64* (FCME); 1,850 m, 13 Mar 1980, *J. Palma G. 63* (XAL); 1,844 m, 1 Apr 2015, *F. Nicolalde-Morejón et al. 2212*–*2214* (CIB); 1,700 m, 24 Mar 1981, *L. Ballesteros & F. Ballesteros 460* (XAL); 1,550 m, 23 Apr 1981, *L.G. Juárez G. 47* (XAL); 1,844 m, 1 Apr 2015, *L. Martínez-Domínguez et al. 515*–*544* (CIB); 1,900 m, 11 Feb 1972, *R. Hernández M. 1507* (MEXU, XAL); 26 Feb 1975, *V. Sosa 59* (XAL).

#### ﻿11. Ceratozamia hildae

Taxon classificationPlantaeCycadalesZamiaceae

G.P.Landry & M.C.Wilson, Brittonia 31(3): 422. 1979

2B539FB3-369F-5343-B7C0-62BA3841291F

Fig. 18

##### Type.

Cultivated in Baton Rouge, Louisiana USA at 5988 South Pollard Parkway (plants originally from several km N of Xilitla, San Luis Potosí, Mexico), *G. Landry 76521*♂ (holotype: GH! [00003274]; isotypes: FTG!, LSU! [LSU00048484], MEXU! [MEXU00443083, MEXU00443084, MEXU00443085], MICH! [1050284A, 1050284B], NY! [00001153–00001156], US! [00011993]).

##### Description.

***Stem*** 10–20 cm long, 10–15 cm in diameter, semi-hypogeous, erect. ***Cataphylls*** 2.1–4.2 × 0.8–1.9 cm wide at the base, persistent, triangular, reddish brown, densely brown tomentose at emergence, partially tomentose at maturity, apex acuminate. ***Leaves*** 2–7, 95–202 cm long, ascending, reddish brown at emergence with whitish gray trichomes, glabrous at maturity. ***Petiole*** 43–89 cm long, terete, linear, greenish brown and green in adult leaves; with 2–12 thin prickles, 0.01–0.2 cm long. ***Rachis*** 60–130 cm long, terete, linear, greenish brown in mature leaves, with prickles and occasionally unarmed. ***Leaflets*** in 5–11 fascicles, 16–56 leaflets in total, clustered, insertion in one plane, oblong, in general longitudinally planar, basally falcate to non-basally falcate, membranaceous, flat, green with adaxial and abaxial sides glaucous, distal end with entire margins, acuminate and symmetrical to asymmetrical at the apex, attenuate at base, with conspicuous and green-light veins; median leaflets 14.5–24 × 2.4–5 cm, 6–15 cm between leaflets; articulations 0.2–0.5 cm wide, brown and green. ***Pollen strobili*** 8–12.5 cm long, 2–2.5 cm in diameter, solitary, cylindrical, erect, brown with reddish brown trichomes at emergence, reddish brown at maturity; peduncle 6–8 cm long, 0.8–1 cm in diameter, tomentose, reddish brown to brown; microsporophylls 0.8–1.2 × 0.6–1 cm, discoid with non-recurved distal face and a lobate fertile portion, infertile portion 0.23–0.29 cm long and rounded with curved horns, 0.20–0.25 cm long, 0.39–0.50 cm and an acute angle between the horns. ***Ovulate strobili*** 10–15 cm long, 6–9 cm in diameter, solitary, cylindrical, erect, green at emergence with brown trichomes, green with brown to blackish trichomes at maturity, acuminate apex; peduncle 7.5–16 cm long, 1.2–1.5 cm in diameter, erect, tomentose, brown; megasporophylls 20–56, 5–8 orthostichies with 4–7 sporophylls per orthostichy, 2–3.7 × 2–4 cm, with a prominent distal face, horns straight and 0.30–0.50 cm long, 1.70–1.90 cm between horns with a right angle between the horns. ***Seeds*** 1.3–2.3 cm long, 1.2–1.5 cm in diameter, ovate, sarcotesta whitish red when immature, light brown at maturity.

##### Distribution and habitat.

*Ceratozamiahildae* is endemic to Sierra Gorda in Mexico, particularly in San Luis Potosí and Querétaro (Fig. 17C), where it occurs in the evergreen tropical forests on karstic rocks at 300–1,200 m.

##### Etymology.

The epithet is in honor of Hilda Guerra Walker, daughter of the original collector (Luciano E. Guerra, plant collector from Mission Texas) (Landry and Wilson 1979).

##### Common names.

Mexico. Querétaro: Chamalillo, pata de gallo (J. Rees 312).

##### Uses.

None recorded.

##### Conservation status.

(IUCN 2021). *Ceratozamiahildae* is listed as “Endangered” under criteria A2abcd; B1ab(ii,iii,iv).

##### Discussion.

**Figure 18.**
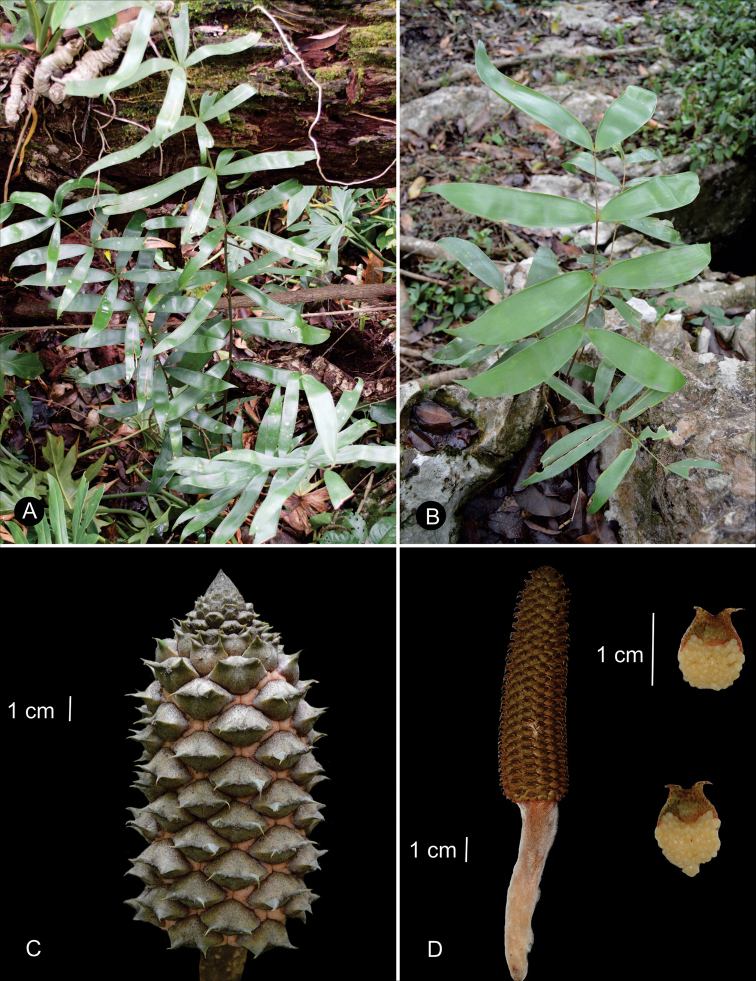
*Ceratozamiahildae***A** leaves of an adult plant **B** leaves of a juvenile plant **C** mature ovulate strobilus **D** mature pollen strobilus and microsporophylls.

*Ceratozamiahildae* is easily distinguished from other members of the group by its clustered membranous oblong leaflets (Fig. 18A).

##### Specimens examined.

Mexico. **Querétaro: Mun. Arroyo Seco**, 1,181 m, 9 Nov 2003, *J.A. Pérez de la Rosa & G. Vargas A. 1908* (IBUG). **Mun. Jalpan de Serra**, 850 m, 29 Dec 1977, *A.P. Vovides 337* (XAL); 29 Dec 1977, *A.P. Vovides s/n* (IEB); 1,200 m, 8 Mar1991, *B. Servin 870* (MEXU); 1,200 m, 20 Mar 1991, *B. Servin 986* (MEXU); 8 Mar 1991, *B. Servin 6328* (IEB); 20 Apr 1991, *B. Servin 6330* (IEB); Sep 1977, *J. Rees s/n* (IEB); *312* (XAL). **San Luis Potosí: Mun. Aquismón**, 300 m, 22 Sep 1977, *A.P. Vovides & J. Rees 312* (MEXU, XAL); 357 m, 20 Mar 2016, *F. Nicolalde-Morejón et al. 2391*–*2406* (CIB); 357 m, 20 Mar 2016, *L. Martínez-Domínguez et al. 910–921, 923, 940* (CIB), *922* (CIB, MEXU); 26 Nov 1970, *S. Longoria s/n* (MEXU; US); 617 m, 15 Jan 2001, *T.W. Walters TW-2001-14-A* (MEXU, XAL), *TW-2001-14-B* (XAL), *TW-2001-14-C* (MEXU).

#### ﻿12. Ceratozamia hondurensis

Taxon classificationPlantaeCycadalesZamiaceae

J.L.Haynes, Whitelock, Schutzman & R.S.Adams, The Cycad Newsletter 31 (2/3): 16. 2008

5541FFD4-1AE6-5C36-A5BC-8A46D45871C4

Fig. 15K

##### Type.

HONDURAS. Atlántida: Mun. Esparta, 0.5 km SE of Jilamo Nuevo along the Río Jilamito, 13 Apr 1994, *A.E. Brand & R. Zúniga 2830*, (lectotype, designated here: MO! [acc. # 5943287–5943289]; isolectotype: MEXU! [MEXU01347996–MEXU01347999]).

##### Description.

***Stems*** 20–50 cm long, 20 cm in diameter, semi-hypogeous, erect and decumbent. ***Cataphylls*** 4.5–7 × 4.5–7.5 cm wide at the base, persistent, triangular, reddish brown, densely brown tomentose at emergence, glabrous at maturity, apex acuminate. ***Leaves*** 3–25, 120–322 cm long, ascending, light green at emergence with whitish gray trichomes, glabrous at maturity. ***Petiole*** 56–150 cm long, terete, linear, green in mature leaves; with 8–34 thin prickles, 0.26–0.50 cm long. ***Rachis*** 80–200 cm long, terete, linear, with prickles, green in mature leaves. ***Leaflets*** 10–18 pairs, opposite to subopposite, insertion in one plane, obovate to broadly oblanceolate, in general longitudinally planar, generally not basally falcate, membranaceous, flat, green with adaxial side glabrous and abaxial side glaucous, distal end with entire margins, acuminate and symmetrical at the apex, attenuate at base, with prominent and light-green veins; median leaflets 23–34.5 × 8–12.8 cm, 8.5–16 cm between leaflets; articulations 0.5–1.6 cm wide, green and yellow. ***Pollen strobili*** 30–35 cm long, 4–5 cm in diameter, solitary, cylindrical, erect, yellowish green at emergence, yellowish cream with blackish trichomes at maturity; peduncle 2–4 cm long, 2–3 cm in diameter, tomentose, brown; microsporophylls 0.9–1.2 × 0.7–1.2 cm, discoid with a non-recurved distal face and lobate fertile portion, infertile portion 0.52–0.60 cm long, and orbicular and rounded with straight horns 0.18–0.23 cm long, 0.50–0.62 cm and an acute angle between the horns. ***Ovulate strobili*** 18–22 cm long, 8–10 cm in diameter, solitary, cylindrical, erect, yellowish green to green with abundant, deep red trichomes at emergence, greenish brown with abundant, blackish trichomes at maturity, mucronate apex; peduncle 5–15 cm long, 1.0–3.0 cm in diameter, erect, tomentose, brown; megasporophylls 72–120, 9–11 orthostichies with 8–11 sporophylls per orthostichy, 1.5–2.5 × 4–5.5 cm, with a prominent distal face, horns curved and 0.60–0.80 cm long, 0.99–1.40 cm between the horns with an acute angle between horns. ***Seeds*** 2.1–2.6 cm long, 1.46–1.89 cm in diameter, ovate, sarcotesta whitish yellow to yellow when immature, light brown at maturity.

##### Distribution and habitat.

*Ceratozamiahondurensis* is endemic to Honduras in the Atlántida department (Fig. 17D), where it grows in evergreen tropical forest between 20–600 m elevation.

##### Etymology.

The specific epithet alludes to the endemism of the species in Honduras.

##### Common names.

Honduras. Atlántida: Camotillo (Haynes et al. 2008).

##### Uses.

The stems are used to elaborate an infusion for poison animals or people. (L. Martínez-Domínguez et al. 2241). Also, the stems are processed for medicinal purposes (Bonta et al. 2019).

##### Preliminary conservation status.

*Ceratozamiahondurensis* is not listed in The IUCN Red List of Threatened Species. There is insufficient data on the population’s status of this species, however, it is known that populations have suffered illegal wildlife trade as shown by seizures made at Customs of Honduras.

##### Discussion.

*Ceratozamiahondurensis* is similar in leaf morphology to *C.euryphyllidia*. It differs in its leaflets with a symmetric apex and an entire margin at distal end as compared to the asymmetric apex and sinuate margin at distal end of the leaflets in *C.euryphyllidia*. Additionally, *C.hondurensis* differs in reproductive structures; the ovulate strobilus has mucronate apex, whereas in *C.euryphyllidia* the ovulate strobilus has long (up to 5 cm) acuminate apex.

In protologue, two specimens from “*A.E. Brand & R. Zúniga 2830*” were cited as types: holotype in EAP and isotype in MO. The type specimen was not deposited in EAP (the herbarium has no record of this material nor was it found in unprocessed material). In addition, we consulted TEFH, the other herbarium in Honduras, with the same result. However, we found a duplicate specimen in MEXU. Thus, we are here designating the specimen in MO cited as isotype as the lectotype and the specimen at MEXU as the isolectotype.

##### Specimens examined.

Honduras. **Atlántida**: **Mun. La Ceiba**, 225 m, 14 Apr 1996, *D.R. Hodel & Schleder 1485* (MO); 217 m, 29 Jul 2003, *J. Haynes et al. 47* (TEFH); 151 m, 19 May 2022, *L. Martínez-Domínguez et al. 2440, 2443, 2444, 2446–2448* (TEFH). **Mun. Tela**, 0–500 m, 10 Feb 1994, *C. Nelson et al. 17586* (TEFH); 200–500 m, 9 Apr 1994, *D.L. Hazlett et al. 8036* (MO); 615 m, 13 Jul 2008, *G. Sandoval et al. 1312* (TEFH); 615 m, 31 Jul 2003, *J. Haynes et al. 40* (TEFH); Cultivated, 615 m, 16 Mar 2017, *L. Ferrufino et al. 777* (TEFH); 163 m, 17 May 2022, *L. Martínez-Domínguez et al. 2421, 2423, 2425, 2428–2430* (TEFH).

#### ﻿13. Ceratozamia huastecorum

Taxon classificationPlantaeCycadalesZamiaceae

Avendaño, Vovides & Cast.-Campos, Bot. J. Linn. Soc. 141(3): 395. 2003

9FBAF21B-3BD9-5E2C-88E4-645B177BC4CB

Fig. 15L

##### Type.

Mexico. Veracruz: Mun. Tepetzintla, Sierra de San Juan Otontepec, 1,300 m, 16 Dec 1981, *G. Castillo-Campos et al. 2567* ♀ (holotype: XAL! [XAL0016937]).

##### Description.

***Stem*** 20–50 cm long, 14–20 cm in diameter, semi-hypogeous, erect and decumbent. ***Cataphylls*** 3.0–4.0 × 2.5–3.0 cm wide at the base, persistent, triangular, reddish brown, densely brown tomentose at emergence, glabrous at maturity, apex acuminate. ***Leaves*** 4–7, 50–80 cm long, ascending, light green at emergence with whitish gray trichomes, glabrous at maturity. ***Petiole*** 20–40 cm long, terete, linear, green in mature leaves; with 10–30 thin prickles 0.21–0.35 cm long. ***Rachis*** 40–70 cm long, terete, linear, green in mature leaves, with prickles. ***Leaflets*** 8–18 pairs, opposite to subopposite, insertion in one plane, oblong, in general longitudinally planar, not basally falcate, coriaceous, flat, green with adaxial side glabrous and abaxial side glaucous, distal end with entire margins, acuminate and asymmetrical at the apex, attenuate at base, with prominent and light-green veins; median leaflets 12–22 × 2.5–6 cm, 1.6–5 cm between leaflets; articulations 0.4–1.1 cm wide, green. ***Pollen strobili*** 15–18 cm long, 2.5–3.2 cm in diameter, solitary, cylindrical, erect, yellowish green at emergence, yellowish cream with blackish trichomes at maturity; peduncle 6–23 cm long, 1.6–2.2 cm in diameter, tomentose, brown; microsporophylls 0.8–1.2 × 0.72–1.0 cm, discoid with a non-recurved distal face and a lobate fertile portion, infertile portion 0.28–0.35 cm long and orbicular with straight horns 0.11–0.18 cm long, 0.50–0.68 cm and an acute angle between the horns. ***Ovulate strobili*** 13.5–18 cm long, 6–10 cm in diameter, solitary, cylindrical, erect, green at emergence, dark green with abundant blackish trichomes at maturity, acuminate apex; peduncle 3–8 cm long, 1.0–2.2 cm in diameter, erect, tomentose, brown; megasporophylls 54–99, 9–10 orthostichies with 6–9 sporophylls per orthostichy, 1.4–1.8 × 1.8–2.0 cm, with a truncate distal face, horns straight and 0.40–0.56 cm long, 1.0–1.60 cm between the horns with an acute angle between the horns. ***Seeds*** 1.1–1.5 cm long, 1.0–1.2 cm in diameter, ovate, sarcotesta light brown at maturity.

##### Distribution and habitat.

**Figure 19.**
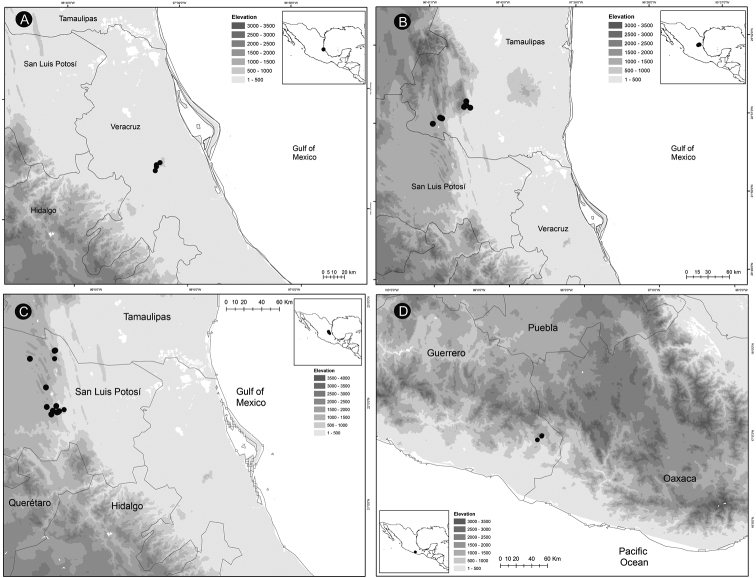
Distribution of *Ceratozamia* species **A***C.huastecorum***B***C.kuesteriana***C***C.latifolia***D***C.leptoceras*.

*Ceratozamiahuastecorum* is endemic to the Sierra de Otontepec in the northwest of Veracruz State, Mexico (Fig. 19A) where it occurs in cloud forest on clay soils with rocky outcrops at 800 to 1,300 m.

##### Etymology.

The specific epithet commemorates the Huasteca region, where this species is found; this region has cultural importance in Veracruz, Mexico.

##### Common names.

Mexico. Veracruz: Palmilla (Bonta et al. 2019).

##### Uses.

None recorded.

##### Conservation status.

(IUCN 2021). *Ceratozamiahuastecorum* is currently listed as “Critically Endangered” under criteria B2ab(iii,v).

##### Discussion.

*Ceratozamiahuastecorum* has a close morphological similarity to *C.latifolia*. However, *C.huastecorum* has light-green emergent leaves and coriaceous leaflets, whereas in *C.latifolia* the leaflets are reddish brown at emergence and papyraceous. In addition, the ovulate strobili are larger than *C.latifolia*, with 9 to 10 orthostichies as compared to *C.latifolia* with 4–7 orthostichies.

##### Specimens examined.

Mexico. **Veracruz: Mun. Tepetzintla**, 1,250 m, 26 Aug 1981, *G. Castillo-Campos & A. Benavides M. 2215* (XAL); 1,200 m, 27 Aug 1981, *G. Castillo-Campos & A. Benavides M. 2232, 2244* (XAL); 820 m, 14 Dec 1981, *G. Castillo-Campos 2480, 2481, 2482* (XAL); 850 m, 10 Jan 1986, *G. Castillo-Campos 4472* (XAL); 20 Sep 1989, *P. Zamora C. et al. 1197* (CH, MEXU, XAL); 21 Sep 1989, *P. Zamora et al. 1249* (MEXU, XAL).

#### ﻿14. Ceratozamia kuesteriana

Taxon classificationPlantaeCycadalesZamiaceae

Regel, Bull. Soc. Imp. Naturalistes Moscou 30: 187. t. 3. 1857

AD3592D8-91D2-5D3C-B414-A71FEFB90639

Figs 3D , 7A , 9B

##### Type.

Cultivated in St. Petersburg, Russia “Ex Horto Petropolitano”, 1856, *E. Regel s.n.* (holotype: LE! [LE00009046]; isotype: U! [U0007272]).

##### Description.

***Stem*** 10–30 cm long, 10–25 cm in diameter, semi-hypogeous, erect. ***Cataphylls*** 1.5–4 × 2–3.5 cm wide at the base, persistent, triangular, reddish brown, densely brown tomentose at emergence, partially tomentose at maturity, apex acuminate. ***Leaves*** 1–11, 80–133 cm long, ascending, reddish brown at emergence, with whitish gray trichomes, glabrous at maturity. ***Petiole*** 30–72 cm long, terete, linear, greenish brown in mature leaves; with 1–10 thin prickles, 0.01–0.15 cm long. ***Rachis*** 40–72 cm long, terete, linear, greenish brown in mature leaves, with prickles and rarely unarmed. ***Leaflets*** 22–50 pairs, opposite to subopposite, insertion in one plane, linear, adaxially curved, basally falcate, papyraceous, caniculate, green, adaxial and abaxial sides glabrous, distal end with entire margins, acuminate and symmetrical at the apex, attenuate at base, with conspicuous and light veins; median leaflets 17–32 × 0.6–1 cm, 0.3–2.5 cm between leaflets; articulations 0.2–0.8 cm wide, brown. ***Pollen strobili*** 11–15 cm long, 1.6–2.5 cm in diameter, solitary, cylindrical, erect, greenish yellow with brown trichomes at emergence, yellowish brown with reddish brown trichomes at maturity; peduncle 8–14 cm long, 0.7–1 cm in diameter, reddish brown to brown pubescent; microsporophylls 0.6–1.1 × 0.5–1 cm, discoid with a non-recurved distal face and a lobate fertile portion, infertile portion 0.28–0.32 cm long and linear with straight horns 0.05–0.25 cm long, 0.45–0.53 cm and an acute angle between the horns. ***Ovulate strobili*** 13–21 cm long, 7–9 cm in diameter, solitary, cylindrical, erect, green at emergence with brown trichomes, grayish light green with black trichomes at maturity, acuminate apex; peduncle 9–16 cm long, 1–2 cm in diameter, erect, tomentose, brown; megasporophylls 30–72, 6–8 orthostichies with 5–9 sporophylls per orthostichy, 2.5–4.0 × 2.3–3.5 cm, with a truncate distal face, horns straight and 0.30–0.52 cm long, 0.60–0.94 cm between horns with an obtuse angle between the horns. ***Seeds*** 1.5–2.3 cm long, 1.2–1.8 cm in diameter, ovate, sarcotesta whitish red when immature, light brown at maturity.

##### Distribution and habitat.

*Ceratozamiakuesteriana* is endemic to Tamaulipas State, Mexico (Fig. 19B), where it occurs on karstic rocks in pine-oak and cloud forests at 1,100–1,500 m.

##### Etymology.

The specific epithet is in honor of Baron K. von Kuester.

##### Common names.

None recorded.

##### Uses.

None recorded.

##### Conservation status.

(IUCN 2021). The status for *Ceratozamiakuesteriana* “Critically Endangered” under criteria A2cd; B2ab(ii,iii,iv).

##### Discussion.

*Ceratozamiakuesteriana* is similar to *C.sabatoi*, but it is easily distinguished by its canaliculate leaflets and ascending leaves.

##### Specimens examined:

Mexico. **Tamaulipas: Mun. Gómez Farías**, 1,260 m, 1 May 1967, *A. Gómez-Pompa 2029* (MEXU); 1,150 m, 12 Aug 1983, *A.P. Vovides 791, 800, 2056* (XAL); 13 Aug 1983, *A.P. Vovides & G. Pattison 771, 772, 801* (XAL); 27 Jun 2017, *C.I. Carvajal-Hernández et al. 1355* (CIB); 1,200–1400 m, 20 Nov 1970, *F. González-Medrano & E. Martínez 3288* (MEXU); 1,100 m, 21 Jan 1970, *F. González-Medrano et al. 3362* (MEXU, MO); 13 Aug 1985, *L. Trejo s/n* (UAT); 1,050 m, 30 Mar 1961, *P.S. Martin & C. Saravia 1170* (ENCB). **Mun. Ocampo**, 1,255 m, 14 Jan 2001, *T.W. Walters TW-201-11-A* (XAL), *TW-201-11-B* (MEXU). **Mun. Tula**, 1,450 m, 21 Jul 1983, *D.W. Stevenson 569H, 569G, 569K* (MEXU); 1,296 m, 19 Mar 2016, *F. Nicolalde-Morejón et al. 2357*–*2365* (CIB); 1,296 m, 19 Mar 2016, *L. Martínez-Domínguez et al. 854*–*867, 869* (CIB), *868* (CIB, MEXU); 1,140 m, 19 Mar 2016, *L. Martínez-Domínguez et al. 870*–*881* (CIB); 24 Apr 2001, *S. Avendaño 5328* (MEXU).

#### ﻿15. Ceratozamia latifolia

Taxon classificationPlantaeCycadalesZamiaceae

Miq., Tijdschr. Wis-Natuurk. Wetensch. Eerste Kl. Kon. Ned. Inst. Wetensch. 1(4): 206. 1848

94684D81-DE30-568F-8178-19575172F967

Figs 7B , 10A

Ceratozamia mexicana Brongn. var. latifolia (Miq.) J.Schust., Pflanzenr. 99: 131. 1932. Type: Based on Ceratozamialatifolia Miq.
Ceratozamia microstrobila Vovides & Rees, Madroño 30: 39. 1983. Type: Mexico. San Luis Potosí: Mun. Ciudad del Maíz, Ejido Las Abritas, km 47 Ciudad Mante-Ciudad del Maíz, 850 m, 7 Nov 1974, *J. Rees 1613* (holotype: XAL! [XAL0099666]; isotypes: FCME! [acc. # 132849], IBUG! [acc. # 155413], MO! [acc. # 5715707], XAL [n.v.]).

##### Type.

Mexico. San Luis Potosí: Route 70, 46 km West of Ciudad Valles, 650 m, 20 Jul 1983, *D.E. Stevenson 565E* (neotype, designated by Stevenson and Sabato 1986, pg. 579: NY! [00001117]; isoneotypes: MEXU! [MEXU00469173, MEXU00469148]).

##### Description.

***Stem*** 5–15 cm long, 10–25 cm in diameter, semi-hypogeous, erect. ***Cataphylls*** 1.5–3 × 2–4 cm wide at the base, persistent, triangular, reddish brown, densely brown tomentose at emergence, partially tomentose at maturity, apex acuminate. ***Leaves*** 1–8, 53–163.5 cm long, descending, reddish brown at emergence, with whitish gray trichomes, glabrous at maturity. ***Petiole*** 25–80 cm long, terete, linear, greenish brown in mature leaves, generally unarmed, rarely up to 3 prickles, 0.08–0.10 cm long. ***Rachis*** 25–110 cm long, terete, linear, greenish brown in mature leaves, unarmed. ***Leaflets*** 7–22 pairs, opposite to subopposite, insertion in one plane, oblong, in general longitudinally planar, basally falcate, papyraceous, flat, green with adaxial and abaxial sides glabrous, distal end with entire margins, acuminate and asymmetrical at the apex, attenuate at base, with conspicuous and indistinct veins; median leaflets 12–28 × 2.3–5.1 cm, 1.7–12.5 cm between leaflets; articulations 0.4–1.1 cm wide, brown. ***Pollen strobili*** 10–20 cm long, 2.1–2.5 cm in diameter, solitary, cylindrical, erect, greenish yellow with reddish brown trichomes at emergence, reddish brown at maturity; peduncle 5–15 cm long, 0.79–1.5 cm in diameter, reddish brown to brown pubescent; microsporophylls 0.5–1.3 × 0.5–1.1 cm, discoid with a non-recurved distal face and a lobate fertile portion, infertile portion 0.20–0.30 cm long and rounded with straight horns 0.1–0.20 cm long, 0.40–0.55 cm and a right angle between the horns. ***Ovulate strobili*** 6.5–16 cm long, 5.5–8.0 cm in diameter, solitary, globose, erect, light green at emergence with brown trichomes, greyish light brown with brown trichomes at maturity, apiculate apex; peduncle 4–13.5 cm long, 1.3–1.8 cm in diameter, erect, brown pubescent; megasporophylls 24–63, 5–7 orthostichies with 4–9 sporophylls per orthostichy, 1.0–2.7 × 1.6–2.9 cm, with a prominent distal face, horns straight and 0.36–0.76 cm long, 0.54–0.63 cm between horns with an obtuse angle between the horns. ***Seeds*** 1.5–2.2 cm long, 1.2–1.6 cm in diameter, ovate, sarcotesta whitish red when immature, light brown at maturity.

##### Distribution and habitat.

*Ceratozamialatifolia* is distributed widely in the mountainous region of San Luis Potosí State, Mexico (Fig. 19C), where it occurs in pine-oak, oak and cloud forests between 600–1,100 m.

##### Etymology.

The specific epithet is derived from the Latin word “latus” (wide) and “folium” (leaves).

##### Common names.

Mexico. San Luis Potosí: Chamalillo, corosillo (S. Sabato et al. 2340); Konlif in the teenek/huasteco language (Bonta et al. 2019).

##### Uses.

The seeds are used to obtain meal for tortillas (Bonta et al. 2019).

##### Conservation status.

(IUCN 2021). The status listed for *Ceratozamialatifolia* is “Endangered” under criteria A2cd+4cd. However, the current circumscription and distribution data indicate that status should be changed to “Vulnerable (V)”.

##### Discussion.

*Ceratozamialatifolia* differs from *C.chamberlainii* by its unarmed petiole or if armed with no more than 3 prickles, its papyraceous leaflets, and discoid microsporophylls with a rounded distal face and horns up to 0.20 cm long. Additionally, this species is characterized by reddish brown leaves at emergence and ovulate strobilus is greyish light brown with brown trichomes at maturity and an apiculate apex.

##### Specimens examined.

Mexico. **San Luis Potosí**: Cultivated, 24 Jan 1994, *F. García S. s/n* (SLPM); Dec 1977, *N.F. McCarten 2552* (ENCB). **Mun. Alaquines**, 1,428 m, 21 Dec 2008, *J. Fortanelli M. & H.A. Castillo 141* (SLPM). **Mun. Cárdenas**, 950 m, 12 to 15 Sep 1967, *J. Rzedowski 24746* (ENCB). **Mun. Ciudad del Maíz**, 750 m, 13 Oct 1968, *H. Puig 3420* (ENCB, P); 25 Apr 2001, *S. Avendaño 5320* (MEXU); 900–1,300 m, 5 Feb 1984, *S. Sabato et al. 2340* (ENCB, MEXU, MO). **Mun. El Naranjo**, 7 Nov 1951, *Clint 144* (US); 20 Jul 1997, *D.W. Stevenson 567* (XAL); 900 m, 19 Mar 2016, *F. Nicolalde-Morejón et al. 2375*–*2389* (CIB); 900 m, 19 Mar 2016, *L. Martínez-Domínguez et al. 894*–*909* (CIB); 895 m, 13 Jan 2001, *T.W. Walters TW-2001-08*; 895 M, 14 Jan 2001, *TW-2001-10A,B* (MEXU, XAL). **Mun. Rayón**, 812 m, 5 Aug 2003, *A.P. Vovides et al. 1466* (XAL); 20 Jul 1983, *D.W. Stevenson 1117* (NY); 650 m, 20 Jul 1983, *D.W. Stevenson 565A* (MEXU), *565B,C* (MEXU, XAL); 993 m, 18. Mar 2016, *F. Nicolalde-Morejón et al. 2320*–*2334* (CIB); 1,100 m, 30 Jun 1962, *F. Medellin L. 1330* (ENCB, MEXU, SLPM); 1,221 m, 6 Apr 2013, *H.A. Castillo-Gómez et al. 558* (SLPM); 600 m, 17 Jul 1988, *J.A. Pérez de la Rosa & L.M. González-Villarreal 1564* (IBUG); 993 m, 18 Mar 2016, *L. Martínez-Domínguez et al. 811*–*827* (CIB); 24 Apr 2001, *S. Avendaño 5282* (MEXU). **Mun. Tamasopo**, 700 m, 15 Aug 2003, *A.P. Vovides et al. 1465* (MEXU, XAL); 2 Jun 1968, *F. Medellin L. 27241* (ENCB); 2 Jun 1968, *F. Medellin L. s/n* (ENCB, IBUG, MEXU, SLPM); 716 m, 18. Mar 2016, *F. Nicolalde-Morejón et al. 2335*–*2348* (CIB); 716 m, 18 Mar 2016, *L. Martínez-Domínguez et al. 828*–*844* (CIB); 750 m, 11 Sep 1967, *J. Rzedowski 24571* (ENCB); 900 m, 24 May 1981, *P.A. Fryxell & W.R. Anderson 3586* (NY; US); 856 m, 19 Jan 2013, *U. Pineda M. 70* (SLPM).

#### ﻿16. Ceratozamia leptoceras

Taxon classificationPlantaeCycadalesZamiaceae

Mart.-Domínguez, Nic.-Mor., D.W.Stev. & Lorea-Hern., PhytoKeys 156: 13. 2020

20036E47-99B6-5E3B-A2EF-245D63B9AD7C

Fig. 7C

##### Type.

Mexico. Guerrero: Mun. Tlacoachistlahuaca, 3 km NW de San Pedro Cuitlapan, 1,400 m, 26 Jun 2019, *L. Martínez-Domínguez & F. Nicolalde-Morejón 1867* ♀ (holotype: CIB! [acc. # 22405UV]; isotypes: MEXU!, NY!).

##### Description.

***Stem*** 30–150 cm long, 11–35 cm in diameter, epigeous, erect to decumbent. ***Cataphylls*** 9–11 × 2.5–3 cm wide at the base, persistent, triangular, reddish brown, densely brown tomentose abaxially at emergence, pubescent at maturity, apex acuminate. ***Leaves*** 7–37 (55), 93.5–281 cm long, descending, green at emergence with sparse reddish brown pubescent, glabrous at maturity. ***Petiole*** 45–85 cm long, terete, linear, copperish green in mature leaves; with 50–75 thin prickles, 0.48–0.68 cm long. ***Rachis*** 75–196 cm long, terete, linear, green in mature leaves, with prickles. ***Leaflets*** 22–61 pairs, opposite to subopposite, insertion in one plane, linear, abaxially curved, not basally falcate, membranaceous, flat, green with adaxial and abaxial sides glabrous, distal end with entire margins, acuminate and symmetrical at the apex, attenuate at base, with conspicuous and greenish veins; median leaflets 28–43.5 × 1.9–2.8 cm, 1.8–2.8 cm between leaflets; articulations 0.70–1.15 cm, generally copperish green. ***Pollen strobili*** 40–45 cm long 6.0–7.8 cm in diameter, generally solitary (rarely 2), cylindrical, erect, brownish yellow at emergence, yellowish green with brownish trichomes at maturity; peduncle 13–19 cm long, 1.5–2.0 cm in diameter tomentose, reddish brown to brown; microsporophylls 2.1–2.45 × 1.09–1.30 cm, obconic with a non-recurved distal face and a deeply lobate fertile portion, infertile portion 0.83–0.96 cm long and linear with straight and thin horns and 0.1–0.23 cm long, 0.44–0.56 cm and an acute angle between the horns. ***Ovulate strobili*** 23.5–28 cm long, 9.5–11 cm in diameter, solitary, cylindrical, erect, brownish green with greyish black trichomes at emergence, copperish green with greyish black pubescent at maturity with an acute apex; peduncle 11–16 cm long, 1.5–2.0 cm in diameter, erect, tomentose, brown; megasporophylls 56–81, 8–9 orthostichies with 7–9 sporophylls per orthostichy, 4.9–5.6 × 2.2–2.6 cm, with a prominent distal face, horns straight and robust and 0.63–0.81 cm long, 0.95–1.35 cm between horns and angle straight between the horns. ***Seeds*** 2.43–2.71 cm in long, 1.4–1.8 cm in diameter, ovoid, sarcotesta whitish pink when immature, light brown at maturity.

##### Distribution and habitat.

*Ceratozamialeptoceras* is endemic to the Sierra Madre del Sur in Guerrero State, Mexico (Fig. 19D), where it occurs on karstic rocks in cloud forest at 1,170–1,400 m.

##### Etymology.

The specific epithet is derived from the Greek words that describe the shape of the horns on the sporophylls: “lepto” for thin or fine and “ceras” in reference to the horns.

##### Common names.

Mexico. Guerrero: Shalukaá is the name used by the “Mixteco” ethnic group (Martínez-Domínguez et al. 2020).

##### Uses.

None recorded.

##### Preliminary conservation status.

Only three populations of *Ceratozamialeptoceras* are known. In particular, one of these populations has few adult plants (approximately 30 individuals). The cloud forests in this area are less affected by anthropogenic pressures; however, this vegetation type is one of the most threatened in Mexico (Williams-Linera 2002). Based upon this information, *C.leptoceras* should be considered “Endangered (EN)” in accordance with IUCN criteria.

##### Discussion.

*Ceratozamialeptoceras* is distinguished from *C.oliversacksii* by its linear and membranaceous leaflets. The main differences are in reproductive structures, *C.leptoceras* has and linear infertile portion of microsporophylls and ovulate strobilus with abundant pubescence at the base of the megasporophylls.

##### Specimens examined.

Mexico. **Guerrero: Mun. Cochoapa El Grande**, 1,170 m, 4 Feb 1984, *F. Lorea-Hernández 2928* (FCME). **Mun. Tlacoachistlahuaca**, 1,200 m, 29 May 219, *F. Nicolalde-Morejón et al. 3173* (XAL), *3174* (FCME), *3175* (CIB); 1,200 m, 29 May 2019, *L. Martínez-Domínguez et al. 1756* (CIB), *1757* (CIB, MEXU), *1758* (XAL), *1759* (CIB, MEXU); 1,400 m, 26 Jun 2019, *F. Nicolalde-Morejón & L. Martínez-Domínguez 3255–3261* (CIB); 1,400 m, 26 Jun 2019, *L. Martínez-Domínguez & F. Nicolalde-Morejón 1860, 1861* (MEXU), *1862–1866* (CIB).

#### ﻿17. Ceratozamia matudae

Taxon classificationPlantaeCycadalesZamiaceae

Lundell, Lloydia 2: 75. 1939

5A1A34A4-0B3C-555F-9948-FF8DBB39964F

Figs 7D , 8D–F , 10D

##### Type.

Mexico. Chiapas: northern slope of Mt. Ovando, 1,000 m, Feb 1939, *E. Matuda 2645* ♂ (holotype: MICH! [1002583]; isotypes: CAS! [0001920], MEXU! [acc. # 86830], US! [00620111]).

##### Description.

***Stem*** 30–50 cm long, 20–30 cm in diameter, epigeous, erect and decumbent. ***Cataphylls*** 4–6.0 × 2.5–4.5 cm wide at the base, persistent, triangular, reddish brown, densely brown tomentose at emergence, partially tomentose at maturity, apex acuminate. ***Leaves*** 3–14, 80–155 cm long, descending, green at emergence with brown trichomes, glabrous at maturity. ***Petiole*** 30–79 cm long, terete, linear, green in adult leaves; unarmed to armed with 18–36 thin prickles, 0.1–0.28 cm long. ***Rachis*** 45–76 cm long, terete, linear, green with abundant brown trichomes in young leaves, yellow to yellowish green in mature leaves, unarmed to armed with prickles. ***Leaflets*** 23–53 pairs, opposite to subopposite, insertion in one plane, lanceolate, longitudinally curved abaxially to planar, not basally falcate, papyraceous, flat, green with base yellow and adaxial and abaxial sides glabrous, distal end with entire margins, acuminate and symmetrical at the apex, attenuate at base, with conspicuous and light-green veins; median leaflets 23–40 × 0.6–1.3 cm, 0.6–1.7 cm between leaflets; articulations 0.3–0.8 cm wide, yellow. ***Pollen strobili*** 9.5–16 cm long, 3.5–5 cm in diameter, solitary, cylindrical, erect, yellowish green with reddish brown trichomes at emergence, yellowish cream with reddish brown trichomes at maturity; peduncle 8–14 cm long, 1.8–2.2 cm in diameter, pubescent, reddish brown to brown; microsporophylls 0.78–1.4 × 0.77–1.1 cm, discoid with a non-recurved distal face and deeply lobate fertile portion, infertile portion 0.37–0.56 cm long and orbicular with straight horns 0.16–0.35 cm long, 0.50–0.81 cm and an acute angle between the horns. ***Ovulate strobili*** 8–15 cm long, 5–8 cm in diameter, solitary, globose, erect, yellowish green with abundant blackish trichomes at emergence, dark green with blackish trichomes at maturity, aristate apex; peduncle 11–17 cm long, 1–2 cm in diameter, pendulous, with trichomes scarce, blackish, pendulous and erect; megasporophylls 16–25, 4–5 orthostichies with 4–6 sporophylls per orthostichy, 2.5–3.4 × 4.5–5.3 cm, with a prominent distal face, horns recurved, straight and thin and 0.59–1.50 cm long, 0.92–1.56 cm between horns with an obtuse angle between the horns. ***Seeds*** 2.9–4.0 cm long, 1.9–4.0 cm in diameter, globose, sarcotesta whitish yellow to yellow when immature, light brown at maturity.

##### Distribution and habitat.

**Figure 20.**
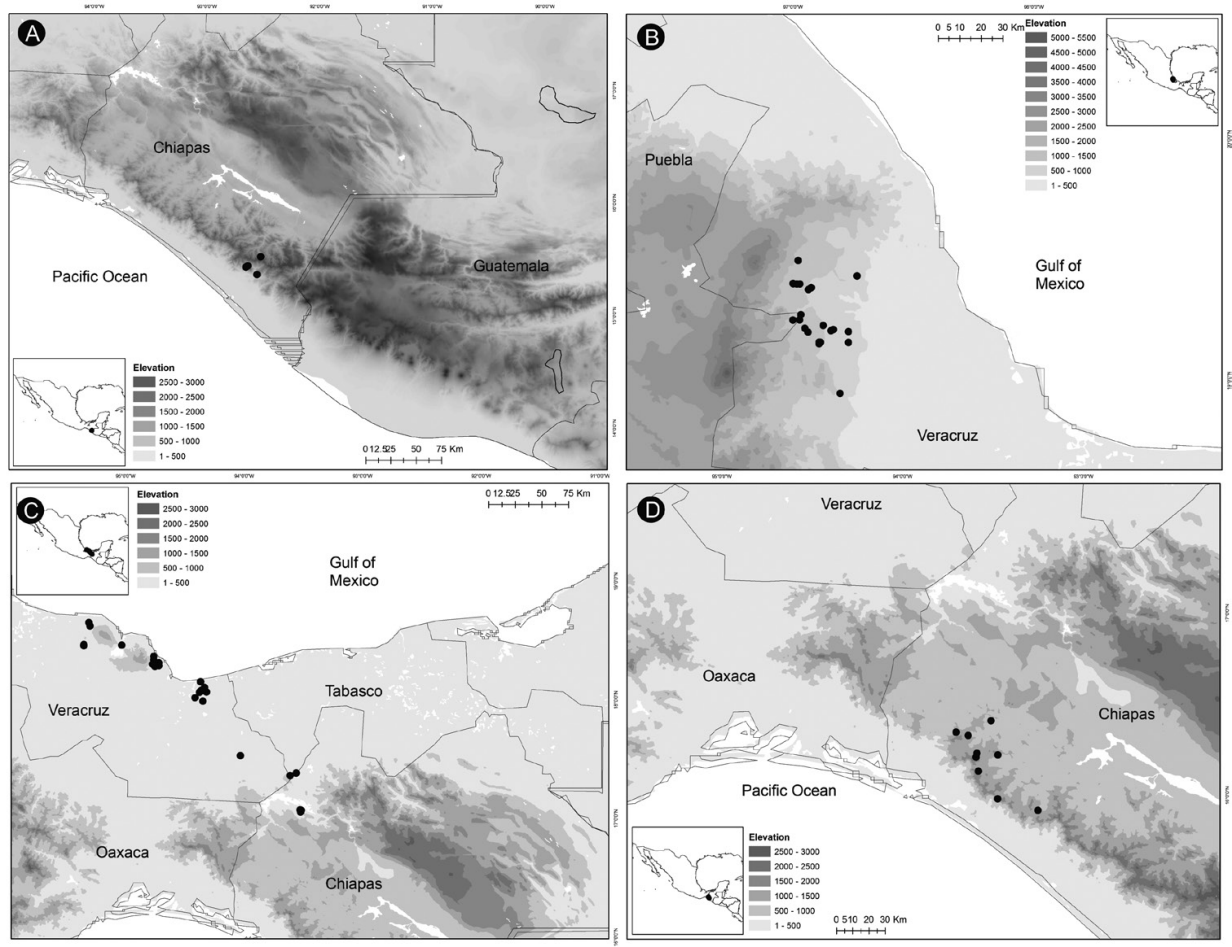
Distribution of *Ceratozamia* species **A***C.matudae***B***C.mexicana***C***C.miqueliana***D***C.mirandae*.

*Ceratozamiamatudae* is endemic to Chiapas in Mexico (Fig. 20A), where it occurs on karstic rocks in evergreen tropical forest at 1,000–1,500 m.

##### Etymology.

The specific epithet is in honor of professor Eizi Matuda, for his contributions to botany in Mexico.

##### Common names.

None recorded.

##### Uses.

None recorded.

##### Conservation status.

(IUCN 2021). Currently, *Ceratozamiamatudae* is listed as “Endangered” under criteria B1ab(ii,iii,v); C1.

##### Discussion.

*Ceratozamiamatudae* differs from other *Ceratozamia* species by its yellow rachis and petiole and leaflets with yellow base and articulations. In addition, the length of the peduncle is the same or longer than the fertile portion of ovulate strobili. The ovulate strobili have an aristate apex, and the megasporophylls have long recurved horns.

##### Specimens examined.

Mexico. **Chiapas: Mun. Acacoyagua**, 1,400 m, *A.P. Vovides 1069* (XAL); 1,300 m, 9 Jan 1987, *D.W. Stevenson et al. 681* (FTG; MO; NY; US); 1,000 m, Feb 1939, *E. Matuda 2646* (MEXU); 1,420 m, 24 Jun 2018, *F. Nicolalde-Morejón et al. 2832–2840* (CIB); 1,420 m, 24 Jun 2018, *L. Martínez-Domínguez et al. 1404*–*1409, 1412, 1415, 1416, 1418, 1419* (CIB), *1405, 1407* (CIB, MEXU); 1,480 m, 29 Dec 1993, *M.A. Pérez-Farrera 27* (CIB, CH, CHIP, MEXU, USCG); 22 Feb 1995, *M.A. Pérez-Farrera 142* (CHIP, FTG, MEXU). **Mun. Escuintla**, 8 Feb 2000, *O. Farrera S. 1875* (CHIP). **Mun. Siltepec**, 1,500 m, 6 Nov 1945, *E.H. Xolocotzi & A.J. Sharp 367* (MEXU); 1,500 m, Oct-Nov 1940, *E. Matuda 4032* (MEXU; NY).

#### ﻿18. Ceratozamia mexicana

Taxon classificationPlantaeCycadalesZamiaceae

Brongn., Ann. Sci. Nat., Bot. sér. 3, 5: 8, t. 1. 1846.

83C42D63-182C-5A58-A086-8E76413D78AB

Figs 7E , 11B

Ceratozamia intermedia Miq., Tijdschr. Wis-Natuurk. Wetensch. Eerste Kl. Kon. Ned. Inst. Wetensch. 1(4): 40. 1848. Type. Mexico. Veracruz: Mun. Puente Nacional, 1 km S of Palmillas, 600 m, 13 Mar 1985, *G. Castillo-Campo & Medina 4275* (neotype, designated by Martínez-Domínguez et al. 2018a, pg. 111: XAL! [acc. # 16924]).
Ceratozamia longifolia Miq., Tijdschr. Wis-Natuurk. Wetensch. Eerste Kl. Kon. Ned. Inst. Wetensch. 1(4): 40. 1848. Type. Mexico. Veracruz: Mun. Zacuapam, Apr 1913, *C. Purpus s.n.* [6362]! (neotype, designated by Martínez-Domínguez et al. 2018a, pg. 111: MO! [acc. # 741393]).
Ceratozamia longifolia Miq. var. minor Miq., Tijdschr. Wis-Natuurk. Wetensch. Eerste Kl. Kon. Ned. Inst. Wetensch. 2(4): 290. 1849. Type. Mexico. Veracruz: Mun. Teocelo, Barranca of Teocelo, 1,020 m, 23 Dec 1975, *M.G. Zola 146* (neotype, designated by Martínez-Domínguez et al. 2018a, pg. 111: XAL! [acc. # 16957]).
Ceratozamia mexicana Brongn. var. longifolia (Miq.) Dyer, Biol. Cent.-Amer., Bot. 3: 193. 1884. Type: Based on Ceratozamialongifolia Miq.

##### Type.

Cultivated in Paris “Hort. Bot. Parisiensis”, original material sent by A. Ghiesbrecht from Mexico, 1845, *Anon. s.n.* (holotype: P! [P02441737, P01637464–P01637466]). Mexico. Veracruz: Mun. Totutla, Barranca El Coyotito, 1 km road from El Mirador to Conejos, 900 m, 8 Sep 1982, *J. Rees & A.P. Vovides 1688* (epitype, designated by Vovides et al. 2016, pg. 427: XAL! [acc. # 17004]).

##### Description.

***Stem*** 20–80 cm long, 20–45 cm in diameter, epigeous, erect and decumbent. ***Cataphylls*** 2–6 × 2.5–6 cm wide at the base, persistent, triangular, reddish brown, densely brown tomentose at emergence, partially tomentose at maturity, apex acuminate. ***Leaves*** 5–55, 100–270 cm long, descending, green at emergence, with brown trichomes, glabrous at maturity. ***Petiole*** 30–93 cm long, terete, linear, dark green in mature leaves; with 8–30 thin prickles, 0.06–0.20 cm long. ***Rachis*** 56–154 cm long, terete, linear, green in mature leaves, with prickles and unarmed. ***Leaflets*** 25–42 pairs, opposite to subopposite, insertion in one plane, lanceolate, in general longitudinally planar, basally falcate, coriaceous, flat, green with adaxial and abaxial sides glabrous, distal end with entire margins, acuminate and symmetrical at the apex, attenuate at base, with conspicuous and green-light veins; median leaflets 29–51 × 2.3–3.7 cm, 1.8–4 cm between leaflets; articulations 0.6–1.5 cm wide, green. ***Pollen strobili*** 20–40 cm long, 4.5–7.6 cm in diameter, generally solitary (1–2), cylindrical, erect, greenish yellow at emergence, greenish yellow with blackish trichomes at maturity; peduncle 3.5–9 cm long, 1.5–2.3 cm in diameter, tomentose, reddish brown to light-brown; microsporophylls 1.5–2.7 × 0.9–2.1 cm, obconic with a non-recurved distal face and a lobate fertile portion, infertile portion 0.47–0.68 cm long and orbicular with straight horns 0.29–0.40 cm long, 0.51–1.05 cm and an acute angle between the horns. ***Ovulate strobili*** 23.5–40 cm long, 8–15 cm in diameter, generally solitary (1–2), cylindrical, erect, dark green with blackish trichomes at emergence, dark green with black and gray trichomes at maturity, acuminate apex; peduncle 7.6–11.5 cm long, 2.5–3 cm in diameter, tomentose, brown to reddish brown, erect or pendulous; megasporophylls 64–240, 8–16 orthostichies with 8–15 sporophylls per orthostichy, 2.0–2.7 × 3.2–5.3 cm, with a prominent distal face, horns straight and 0.53–1.2 cm long, 1–2.1 cm between horns and a right angle between the horns. ***Seeds*** 2.0–3.3 cm long, 1.4–2.5 cm in diameter, ovate, sarcotesta whitish yellow to yellow when immature, light brown at maturity.

##### Distribution and habitat.

*Ceratozamiamexicana* is endemic to Veracruz State, Mexico from the River La Antigua drainage system to the southern end of the Sierra Madre Oriental (Fig. 20B). It occurs in cloud forest on karstic rocks at 500–1,100 m.

##### Etymology.

The specific epithet is derived from the country of origin of the material used for the description of this species. *Ceratozamiamexicana* was the first species described in the genus.

##### Common names.

None recorded.

##### Uses.

None recorded.

##### Conservation status.

(IUCN 2021). *Ceratozamiamexicana* is listed as “Vulnerable” under criteria A2acd+4cd. According to the current circumscription, this species only occurs in a narrow area, which suffered dramatic decline in forest (Martínez-Domínguez et al. 2021). Besides, the populations visited by us have only between 30 and 50 adult plants. This data suggest *C.mexicana* should be listed as “Critically Endangered (CR)” under criteria B1ab(iiii, iv)

##### Discussion.

*Ceratozamiamexicana* is similar to *C.tenuis*, *C.morettii* and *C.brevifrons*, but it can be easily distinguished from vegetative and reproductive chracters. This species has lanceolate leaflets, whereas *C.tenuis* and *C.morettii* have linear and oblong leaflets, respectively. In addition, *C.mexicana* has insertion in one plane for leaflets and thin prickles on petiole, whereas *C.brevifrons* has keeled leaflets and petioles armed with robust prickles.

##### Specimens examined.

Mexico. **Veracruz: Mun. Coatepec**, 1,600 m, 26 Jun 1990, *P. Zamora C. 2450* (MEXU, XAL). **Mun. Comapa**, 1,003 m, 18 Feb 2013, *F. Nicolalde-Morejón et al. 1732*–*1734* (CIB); 1,003 m, 28 Sep 2014, *F. Nicolalde-Morejón et al. 2145*–*2156* (CIB); 970 m, 13 Nov 2015, *F. Nicolalde-Morejón et al. 2269*–*2272* (CIB); 1,003 m, 28 Sep 2014, *L. Martínez-Domínguez et al. 164* (CIB); 970 m, 13 Nov 2015, *L. Martínez-Domínguez & F. Nicolalde-Morejón 716*–*723, 725*–*730* (CIB), *724* (CIB, MEXU). **Mun. Puente Nacional**, 600 m, 13 Mar 1985, *G. Castillo-Campos & M. E. Medina 4299* (XAL). **Mun. Sochiapa**, 1,058 m, 9 Jul 2008, *M. Vázquez-Torres 8589* (CIB). **Mun. Teocelo**, 900 m, 16 Feb 1997, *L.H. Bojórquez-Galván et al. 531* (CIB); 1,070 m, 20 Nov 2015, *F. Nicolalde-Morejón et al. 2273*–*2278* (CIB); 1,065 m, 20 Nov 2015, *L. Martínez-Domínguez et al. 731* (CIB); 1,070 m, 20 Nov 2015, *L. Martínez-Domínguez et al. 732–733, 735*–*750* (CIB), *734* (CIB, MEXU); 1,070 m, 11 Mar 2016, *L. Martínez-Domínguez et al. 764*, 765, 767–*770* (CIB), *766* (CIB, MEXU); 740–800 m, 20 Jul 1995, *M. Vazquez-Torres* 4865 (CIB). **Mun. Tlaltetela**, 1,196 m, 5 Jun 2015, *F. Nicolalde-Morejón & L. Martínez-Domínguez 2242* (CIB); 1,084 m, 5 Jun 2015, *F. Nicolalde-Morejón & L. Martínez-Domínguez 2243* (CIB); 1,196 m, 10 Jun 2015, *F. Nicolalde-Morejón & L. Martínez-Domínguez 2246* (CIB); 1,130 m, 21 Jun 2015, *F. Nicolalde-Morejón & L. Martínez-Domínguez 2253*–*2257* (CIB); 1,084 m, 5 Jun 2015, *L. Martínez-Domínguez & F. Nicolalde-Morejón 584*–*586* (CIB); 1,196 m, 10 Jun 2015, *L. Martínez-Domínguez & F. Nicolalde-Morejón 620* (CIB); 1,130 m, 21 Jun 2015, *L. Martínez-Domínguez & F. Nicolalde-Morejón 628*–*635* (CIB). **Mun. Totutla**, 900 m, 23 Feb 1982, *A.P. Vovides 730*–*733* (XAL); 900 m, 8 Sep 1982, *A.P. Vovides 748* (XAL); 900 m, 9 Oct 1993, *Brigada T. Walters s/n* (XAL); 875 m, 8 Aug 2015, *F. Nicolalde-Morejón et al. 2262* (CIB); 864 m, 27 Jan 2016, *F. Nicolalde-Morejón et al. 2279*–*2281* (CIB); *F. Vázquez B. 730* (XAL); 1,094 m, 21 Jun 2016, *J.M. Ramírez-Amezcua & A. Paizanni Guillén 715* (MEXU); 8 Sep 1982, *J. Rees 6344* (IEB); 800 m, 30 Dec 1975, *J. Rees & A.P. Vovides 1660, 1672* (XAL); 900 m, 8 Sep 1982, *J. Rees & A.P. Vovides 1689* (XAL); 875 m, 8 Aug 2015, *L. Martínez-Domínguez et al. 704*–*707* (CIB); 864 m, 27 Jan 2016, *L. Martínez-Domínguez et al. 752*, *754–757* (CIB, MEXU), *758* (CIB). **Mun. Xico**, 1,195 m, 10 Jul 2015, *L. Martínez-Domínguez & F. Nicolalde-Morejón 640*–*646* (CIB); 1,159 m, 10 Jul 2015, *L. Martínez-Domínguez & F. Nicolalde-Morejón 647*, *648* (CIB); 900 m, 13 Nov 1981, *M. Nee 23035* (NY).

#### ﻿19. Ceratozamia miqueliana

Taxon classificationPlantaeCycadalesZamiaceae

H.Wendl., Index Palm. 68. 1854

19E22097-7356-5A98-A0C6-3E75C328118A

Figs 7F , 8A–C , 9D , 11A

Ceratozamia mexicana Brongn. var. miqueliana (H.Wendl.) J. Schust., Pflanzenr. (Engler) Heft 99, 4 fam 1: 131. 1932. Type: Based on Ceratozamiamiqueliana H. Wendl.

##### Type.

Mexico. Veracruz: West of Santiago Tuxtla, Cerro el Vigía, 5 Jul 1983, *D.W. Stevenson 542 F* (neotype, designated by Stevenson and Sabato 1986, pg. 580: NY! [00001118]).

##### Description.

***Stem*** 45–75 cm long, 16–18 cm in diameter, epigeous, erect and decumbent. ***Cataphylls*** 3–7 × 1.5–4 cm wide at the base, persistent, triangular, reddish brown, densely brown tomentose at emergence, glabrous at maturity, apex acuminate. ***Leaves*** 12–37, 35–261 cm long, ascending, light green and glaucous at emergence with brown trichomes, glabrous at maturity. ***Petiole*** 31–97 cm long, terete, linear, pink at emergence, green in mature leaves; with 8–60 robust prickles, 0.22–0.59 cm long. ***Rachis*** 41–164 cm long, terete, linear, pink at emergence, yellowish green in mature leaves, with prickles. ***Leaflets*** 12–23 pairs, opposite to subopposite, insertion in one plane, oblong, in general longitudinally planar, not basally falcate, papyraceous, flat, green with abaxial and adaxial sides glabrous, acuminate and asymmetrical at the apex, distal end with entire margins, attenuate at base, with conspicuous and green-light veins; median leaflets 16–36 × 4.4–8.7 cm, 2.9–8.5 cm between leaflets; articulations 0.3–1.8 cm wide, greenish. ***Pollen strobili*** 15–30 cm long, 3.1–4.2 cm in diameter, usually solitary (1–2), cylindrical, erect, greenish yellow with red trichomes at emergence, greenish yellow to cream at maturity; peduncle 3.5–5.0 cm long, 1.9–2.2 cm in diameter, tomentose, light brown; microsporophylls 1–2 × 0.7–1.5 cm, obconical with a non-recurved distal face and deeply lobate fertile portion, infertile portion 0.35–0.50 cm long and rounded with straight horns 0.30–0.42 cm long, 0.52–0.70 cm and an acute angle between the horns. ***Ovulate strobili*** 22–30 cm long, 8.9–12 cm in diameter, solitary, cylindrical, erect, green with blackish trichomes, yellowish green with blackish trichomes at maturity, acuminate apex; peduncle 7–12 cm long, 2.7–3.3 cm in diameter, erect and pendulous, tomentose, light brown; megasporophylls 64–110, 8–11 orthostichies with 8–10 sporophylls per orthostichy, 2.0–3.0 × 3.5–4.2 cm, with a prominent distal face, horns straight and 0.35–0.43 cm long, 1.06–1.12 cm between horns with a right angle between the horns. ***Seeds*** 2.4–3 cm long, 1.3–1.8 cm in diameter, ovate, sarcotesta whitish yellow to yellow when immature, light brown at maturity.

##### Distribution and habitat.

*Ceratozamiamiqueliana* is endemic to southwest Mexico in Chiapas, Tabasco and Veracruz States (Fig. 20C), where it occurs on shallow soils in evergreen tropical rain forest at 19–1,000 m.

##### Etymology.

The specific epithet was assigned in honor of Friedrich Anton Wilhelm Miquel.

##### Common names.

Mexico. Veracruz: Palmita (J. Rees 1657).

##### Uses.

The leaves are used for decorations during festivities in Santiago Tuxtla (Veracruz).

##### Conservation status.

(IUCN 2021). *Ceratozamiamiqueliana* is listed as “Critically Endangered” under criteria A2acd. This species has a wide distribution; however, the evergreen tropical rain forest in Veracruz has declined dramatically in recent years (Guevara et al. 2004). In addition, most of the populations occur in areas of interest for the oil industry.

##### Discussion.

*Ceratozamiamiqueliana* differs from its congeners by its robust and long prickles, and papyraceous, oblong and glabrous (lustrous) leaflets. In addition, ovulate strobili are yellowish yellowish green with blackish trichomes and have an acuminate apex at maturity. This species is closely related to *C.zoquorum*, however, that species has descending leaves with coriaceous leaflets.

##### Specimens examined.

Mexico. **Chiapas: Mun. Ocozocoautla de Espinosa**, 421 m, 23 Oct 1997, *R. Juárez-Galdamez 4* (CHIP, MO, XAL); 800 m, 21 Jan 2001, *S. Avendaño 5223* (MEXU); 765 m, 21 Jan 2001, *T.W. Walters 2001–2036-A* (MEXU); *2001–2036-C* (XAL). **Tabasco: Mun. Huimanguillo**, 405 m, 10 Aug 2013, *F. Nicolalde-Morejón et al. 1826–1846* (CIB); 23 Jun 1998, *G. Ortíz G. 5291* (MEXU). **Veracruz: Mun. Ángel R. Cabada**, 231 m, 29 Oct 2013, *F. Nicolalde-Morejón et al. 1868–1887* (CIB); 30 Oct 2013, *F. Nicolalde-Morejón et al. 1888–1909* (CIB). **Mun. Catemaco**, 31 Oct 2013, *F. Nicolalde-Morejón et al. 1910–1929* (CIB). **Mun. Coatzacoalcos**, 22 m, 2 Apr 2003, *C.H. Ramos 2266* (MEXU); Jun 1960, *E. Hernández X. et al. 162* (CHAPA); 26 Jun 1997, *G. Castillo C. & F. Morocini 16267* (XAL); 30 m, 7 Sep 2005, 30 m, *L.H. Bojórquez-Galván 1601* (CIB); 18 Jan 2001, *S. Avendaño R. 5214* (MEXU); 31 m, 18 Jan 2001, *T.W. Walters 2001–21-A, B* (XAL); *T.W. Walters 2001-21-D* (MEXU). **Mun. Ixhuatlán del Sureste**, 45 m, 13 Sep 2011, *J. Calónico-Soto & B. Gómez C. 27780* (MEXU). **Mun. Las Choapas**, 115 m, 26 Nov 2011, *A. Rincón G. 2894* (XAL); 115 m, 30 Jun 2003, *E. López P. 634* (XAL); 100 m, 25 Nov 2004, *F. Nicolalde-Morejón et al. 1434* (XAL). **Mun. Mecayapan**, 13 Jul 1994, *G. Castillo-Campos et al. 12,565* (ENCB, XAL); 9 Mar 1995, *G. Castillo-Campos et al. 13470, 13489* (XAL); 5 Aug 1985, *J.I. Calzada 11207* (CH, MEXU); 300 m, 16 Jul 1982, *M. Nee et al. 25066* (XAL), *25118* (MO, NY, XAL); 950 m, 26 Jan 1992, *M. Vázquez- Torres 4123* (CIB); 5 May 1995, *M. Vázquez- Torres 5017* (CIB). **Mun. Moloacán**, 60 m, 19 Dec 1974, *J. Rees 1657* (MEXU, XAL), *1658* (XAL), *6347, 6348* (IEB). **Mun. Nanchital**, 34 m, 12 Aug 2011, *J. Rivera H. et al. 4530* (XAL). **Mun. Santiago Tuxtla**, 900–1,000 m, 5 Jul 1983, *D.W. Stevenson et al. 541 A–C– F, I, K* (NY), *G, H, J, N* (MEXU, NY); 5 Jul 1983, *D.W. Stevenson et al. 542 A–E, H* (NY); 650 m, 28 Mar 2014, *F. Nicolalde-Morejón & L. Martínez-Domínguez 1988–1998* (CIB); 950 m, 24 Jan 1972, *J.H. Beaman 5507* (XAL); 650 m, 28 Mar 2014, *L. Martínez-Domínguez & F. Nicolalde-Morejón 88–106* (CIB), *107* (CIB, MEXU); 800 m, 28 May 1967, *L. Scheinvar 673* (MEXU); 29 Aug 1967, *M. Sousa 3201* (MEXU); 845 m, 17 Jan 2001, *S. Avendaño R. 5207* (MEXU); 845 m, 17 Jan 2001, *T.W. Walters et al. TW 2001–2019* (MEXU, XAL). **Mun. Pajapan**, 830–980 m, 15 Jul 1982, *M. Nee et al. 25066* (XAL). **Mun. Soteapan**, 250 m, 13 May 1986, *M. Vázquez-Torres 3360* (CIB, XAL). **Mun. Tatahuicapan de Juárez**, 849 m, *C.I. Carvajal & M.J. Fragoso 797* (CIB); 15 Mar 2008, *L H. Bojórquez-Galván et al. 1939, 1946* (CIB).

#### ﻿20. Ceratozamia mirandae

Taxon classificationPlantaeCycadalesZamiaceae

Vovides, Pérez-Farr. & Iglesias, Bot. J. Linn. Soc. 137: 81. 2001

E90B352E-7ECE-57BA-841E-097AA650C2F5

Fig. 7G

##### Type.

Mexico. Chiapas: Mun. Villa Flores, Ejido La Sombra de la Selva, 880 m, 20 Sep 1997, *J. De La Cruz Rodríguez 66* (lectotype, designated here: MEXU! [MEXU00934924, MEXU00934907, MEXU00934905]).

##### Description.

***Stem*** 20–60 cm long, 20–30 cm in diameter, epigeous, erect and decumbent. ***Cataphylls*** 3–8.0 × 1.5–4.0 cm wide at the base, persistent, triangular, reddish brown, densely brown tomentose at emergence, partially tomentose at maturity, apex acuminate. ***Leaves*** 2–50 (150), 46–242.5 cm long, ascending, brown at emergence with whitish gray trichomes, glabrous at maturity. ***Petiole*** 16–98 cm long, terete, linear, brown in mature leaves; with 11–46 thin prickles, 0.30–0.40 cm long. ***Rachis*** 26–144.5 cm long, terete, linear, brown to greenish brown in mature leaves, lower 2/3rds with prickles. ***Leaflets*** 28–94 pairs, opposite to subopposite, insertion in one plane, linear to lanceolate, in general longitudinally planar, not basally falcate, papyraceous, lightly involute, green with adaxial and abaxial sides glabrous, distal end with entire margins, acuminate and symmetrical at the apex, attenuate at base, with conspicuous and light-green veins; median leaflets 10–40 × 0.4–0.9 cm, 0.2–1.6 cm between leaflets; articulations 0.12–1.20 cm wide, brown. ***Pollen strobili*** 16–55 cm long, 4.0–7.7 cm in diameter, solitary, cylindrical, erect, yellowish green with brown trichomes at emergence, yellowish cream with blackish trichomes at maturity; peduncle 5–11 cm long, 0.8–1.4 cm in diameter, pubescent, reddish brown to brown; microsporophylls 1.23–1.80 × 0.62–0.86 cm, elliptic with a non-recurved distal face and a lobate fertile portion, infertile portion 0.62–0.81 cm long and linear with straight horns 0.17–0.39 cm long, 0.56–0.95 cm and an obtuse to acute angle between the horns. ***Ovulate strobili*** 13.5–35 cm long, 7–12.6 cm in diameter, solitary, cylindrical, erect, yellowish green with abundant blackish trichomes at emergence, glaucous green with reddish brown to blackish trichomes at maturity, acuminate apex; peduncle with trichomes scarce, brownish, 4.5–12 cm long, 1.0–2.2 cm in diameter, erect; megasporophylls 42–72, 6–8 orthostichies with 7–10 sporophylls per orthostichy, 2.0–3.0 × 2.8–6.1 cm, with a prominent distal face, horns straight and 0.29–1.47 cm long, 1.55–2.09 cm between horns with an obtuse angle between the horns. ***Seeds*** 1.9–2.9 cm long, 1.2–2.5 cm in diameter, spherical, sarcotesta whitish pink when immature, light brown at maturity.

##### Distribution and habitat.

*Ceratozamiamirandae* is widely distributed in Chiapas State, Mexico from “Tres Picos” hill and Sierra Morena to a mountain range in Mun. Jiquipilas (Fig. 20D); it occurs on karstic rocks with abundant organic material in oak forest and transitional oak forest between pine oak forest and cloud forest between 850 and 1,500 m.

##### Etymology.

This species was named in honor of Dr. Faustino Miranda for his untiring contributions to the flora of Chiapas (Vovides et al. 2001).

##### Common names.

Mexico. Chiapas: Amenduai (L. Martínez-Domínguez et al. 1428; M.A. Pérez-Farrera 1261A), Espadaña de cochi (M.A. Pérez-Farrera 26A), Peinetilla (M.A. Pérez-Farrera 37).

##### Uses.

The mature seeds are used as food (U. Bachem C. & R. Rojas 579).

##### Conservation status.

(IUCN 2021). *Ceratozamiamirandae* is listed as “Endangered” under criteria C1.

##### Discussion.

*Ceratozamiamirandae* is similar to *C.alvarezii*. However, in *C.mirandae*, the leaves and ovulate strobilus are larger at the population level than in *C.alvarezii*, which is a more diminutive species overall.

In the original publication of *C.mirandae*, there were no isotypes cited, only the holotype in CHIP. Because no holotype of *C.mirandae* has been found in CHIP, despite intensive searches, we designate the uncited isotype found in MEXU as the lectotype.

##### Specimens examined.

Mexico. **Chiapas: Mun. Jiquipilas**, 1,170 m, 19 Jun 2018, *F. Nicolalde-Morejón et al. 2749–2759* (CIB); 1,015 m, 8 Mar 1995, *J.J. Castillo-Hernández 548* (CHIP); 1,170 m, 19 Jun 2018, *L. Martínez-Domínguez et al. 1312–1316, 1318, 1320, 1321* (CIB), *1317* (CIB, MEXU), *1319* (CIB, MEXU); 1,270 m, 25 May 1995, *M.A. Pérez-Farrera 465* (CHIP). **Mun. Villa Corzo**, 1,320 m, 12 Jul 2004, *A. Reyes-García et al. 7134* (MEXU); 1,500 m, 9 Feb 1972, *D.E. Breedlove 23999* (MEXU, MO); 1,170 m, 16 Mar 1989, *U. Bachem & R. Rojas C. 579* (CHIP, MEXU, SLPM). **Mun. Villaflores**, 960 m, 6 Apr 1995, *A.P. Vovides & M.A. Pérez-Farrera 1261* (XAL); 1,157 m, 7 Jul 2004, *D. Álvarez 9809* (MEXU); 1,277 m, 25 Jun 2018, *F. Nicolalde-Morejón et al. 2854–2863* (CIB); 1,195 m, 20 Jun 2019, *F. Nicolalde-Morejón & L. Martínez-Domínguez 3208–3213* (CIB); 1,350 m, 21 Jun 2019, *F. Nicolalde-Morejón & L. Martínez-Domínguez 3215–3224* (CIB); 1,015 m, 5 Aug 1994, *J.J. Castillo-Hernández 230* (CIB, MEXU, USCG); 1,250 m, 5 Apr 1995, *J.J. Castillo-Hernández* 595 (CHIP); 1,140 m, 6 Aug 2002, *L. Alvarado C. et al. 368* (MEXU); 1,195 m, 20 Jun 2019, *L. Martínez-Domínguez & F. Nicolalde-Morejón 1808–1813* (CIB); 1,350 m, 21 Jun 2019, *L. Martínez-Domínguez & F. Nicolalde-Morejón 1814–1825* (CIB); 1,277 m, 25 Jun 2018, *L. Martínez-Domínguez et al. 1428, 1429, 1431–1438* (CIB), *1430* (CIB, MEXU); 940 m, 22 Dec 1993, *M.A. Pérez-Farrera s/n* (XAL), *26* (CHIP, XAL); 910 m, 28 Apr 1994, *M.A. Pérez-Farrera 37* (CHIP, XAL); 1,460 m, 16 Sep 1994, *M.A. Pérez-Farrera 126* (CHIP); 950 m, 12 Apr 1996, *M.A. Pérez-Farrera 1480* (CHIP).

#### ﻿21. Ceratozamia mixeorum

Taxon classificationPlantaeCycadalesZamiaceae

Chemnick, T.J.Greg. & Salas-Mor., Phytologia 83(1): 47 1998 (“1997”)

AF657135-F53A-5B7B-BCCD-310DF063E586

Fig. 7H

##### Type.

Mexico. Oaxaca: Cercanía a Juquila Mixe, 1,737 m, 21 May 1997. *J. Chemnick & T. Gregory 49* (lectotype, designated by Nicolalde-Morejón and Avendaño 2011, pg. 1033: XAL! [XAL0065870]).

##### Description.

***Stem*** 30–100 cm long, 18–25 cm in diameter, epigeous, decumbent. ***Cataphylls*** 1.8–4.6 × 1.9–4.3 cm wide at the base, persistent, triangular, reddish brown, densely brown tomentose at emergence, partially tomentose at maturity, apex acuminate. ***Leaves*** 3–17 (30), 85–246 cm long, descending, light green and glaucous at emergence with light brown trichomes, glabrous at maturity. ***Petiole*** 46–132 cm long, terete, linear, green in mature leaves; with 30–50 thin prickles, 0.20–0.38 cm long. ***Rachis*** 58–118 cm long, terete, linear, green to greenish in mature leaves, with scarce prickles. ***Leaflets*** 19–35 pairs, opposite to subopposite insertion in one plane, lanceolate, abaxially curved, rarely planar, not basally falcate, papyraceous, flat, green with adaxial and abaxial sides glabrous, distal end with entire margins, acuminate and symmetrical at the apex, attenuate at base, with conspicuous and green-light veins; median leaflets 24–39 × 1.9–2.9 cm, 1.7–3.0 cm between leaflets; articulations 0.6–1.0 cm wide, green. ***Pollen strobili*** 22–30 cm long, 4.5–7 cm in diameter, generally solitary (1–2), cylindrical, erect, greenish at emergence with reddish trichomes, greenish yellow with reddish brown trichomes at maturity; peduncle 10–15 cm long, 1.2–2.0 cm in diameter, glabrous or with trichomes scarce reddish brown to brown; microsporophylls 1.4–2.4 × 0.7–1.3 cm, obconic with a non-recurved distal face and lobate fertile portion, infertile portion 0.39–0.48 cm long and linear with straight horns 0.30–0.50 cm long, 0.50–0.93 cm and a right angle between the horns. ***Ovulate strobili*** 23–32 cm long, 12–16 cm in diameter, solitary, cylindrical, erect, yellowish green with abundant blackish trichomes at emergence, green with brown to blackish trichomes at maturity, apiculate apex; peduncle 12–23.5 cm long, 1.3–2.1 cm in diameter, pendulous, glabrous or with scarce reddish brown trichomes; megasporophylls 60–112, 6–9 orthostichies with 10–14 sporophylls per orthostichy, 2.4–2.8 × 4.0–5.1 cm, with a truncate distal face, horns straight and 0.90–1.1 cm long, 0.92–1.2 cm between horns and a right angle between the horns. ***Seeds*** 2.2–3.0 cm long, 1.6–2.0 cm in diameter, ovate, sarcotesta whitish yellow to yellow when immature, light brown at maturity.

##### Distribution and habitat.

**Figure 21.**
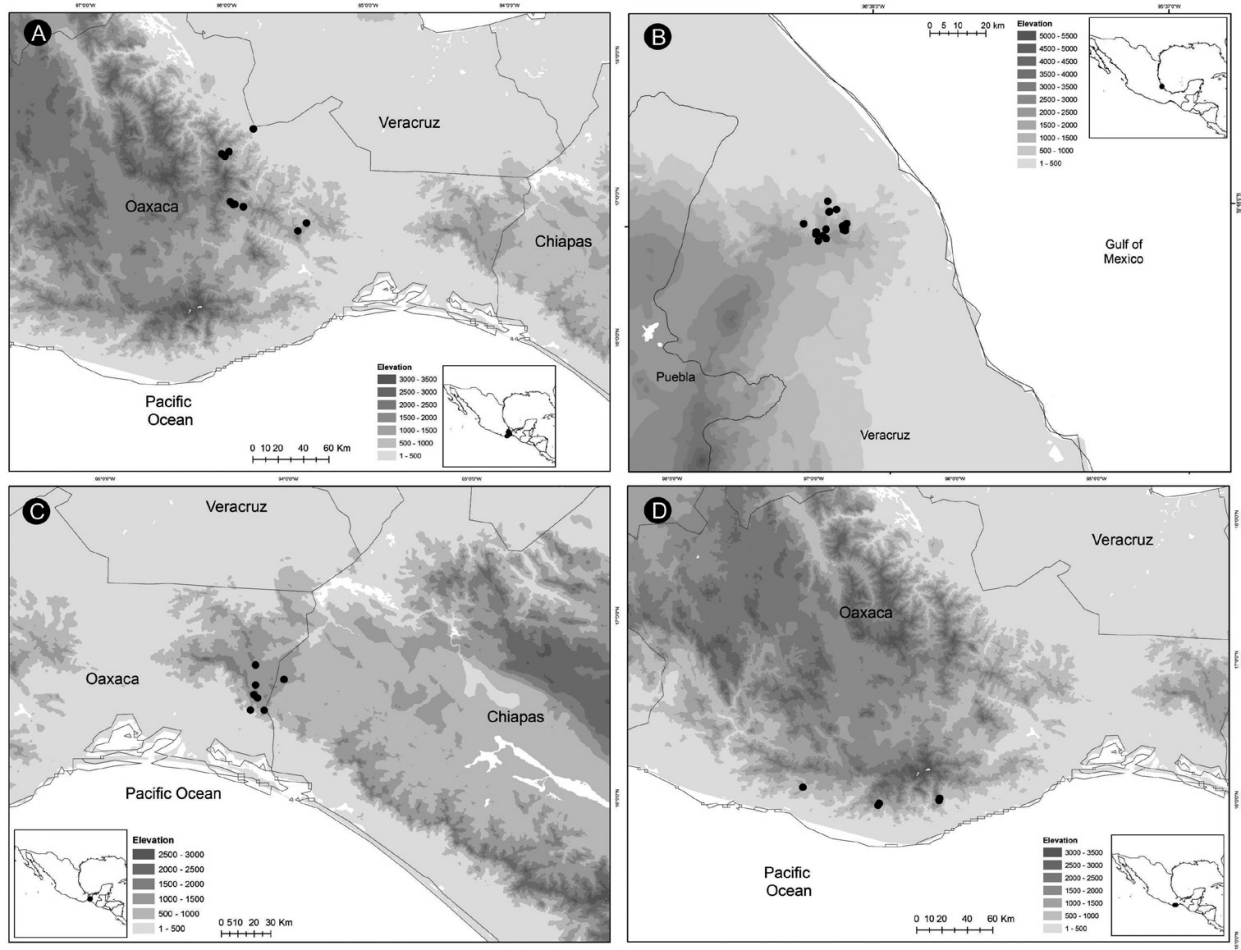
Distribution of *Ceratozamia* species **A***C.mixeorum*. **B***C.morettii*. **C***C.norstogii***D***C.oliversacksii*.

*Ceratozamiamixeorum* is endemic to the southern portion of Sierra Norte of Oaxaca State (Mexico), along the highlands in the Mixe area (Fig. 21A), where it occurs in karstic rocks of cloud and oak forests between 1,170 to 2,150 m.

##### Etymology.

The specific epithet was named in honor of the Mixe people.

##### Common names.

Mexico. Oaxaca: Carrete (Chemnick et al. 1998).

##### Uses.

The pollen strobilus is used as a toy (Chemnick et al. 1998).

##### Conservation status.

(IUCN 2021). *Ceratozamiamixeorum* is listed under criteria A2cd+4cd; B1ab(ii,iii,v) as “Endangered”.

##### Discussion.

*Ceratozamiamixeorum* is similar to *C.whitelockiana*, but has prickles on the rachis of the leaf and a densely prickly petiole (up to 50 prickles), ovulate strobili with long and pendulous pedunclse up to 23.5 cm long. The fertile portion of ovulate strobilus has 6–9 orthostichies and 10–14 sporophylls per orthostichy; in contrast to 4–5 orthostichies and 5–8 sporophylls per orthostichy in *C.whitelockiana*.

##### Specimens examined.

Mexico. **Oaxaca: Mun. Guevea de Humboldt**, 1,300 m, 30 Mar 1991, *A. Campos 3614* (MEXU). **Mun. San Juan Juquila Mixes**, 1,605 m, 7 Apr 1995, *E. Torres B. 563* (SERO, XAL); 1,595 m, 25 Sep 2020, *F. Nicolalde-Morejón et al. 3368* (CIB); 1,681 m, 25 Sep 2020, *F. Nicolalde-Morejón et al. 3369–3371* (CIB); 1,595 m, 25 Sep 2020, *L. Martínez-Domínguez et al. 2001, 2002* (CIB); 1,681 m, 25 Sep 2020, *L. Martínez-Domínguez et al. 2003* (CIB, MEXU), *2004–2006* (CIB). **Mun. San Pedro Ocotepec**, 8 Apr 1995, 1,600 m, *E. Torres B. et al. 607* (XAL). **Mun. Santa María Guienagati**, 1,200 m, 25 Jan 1996, *M. Cerón C. et al. 249* (MEXU, SERO, XAL). **Mun. Santa María Tepantlali**, 1,728 m, 25 Sep 2020, *F. Nicolalde-Morejón et al.* 3362–3367 (CIB); 2,147 m, 15 May 2011, *G. Juárez-García 3871* (MEXU); 1,728 m, 25 Sep 2020, *L. Martínez-Domínguez et al. 1994–1995* (CIB, MEXU), *1996–2000* (CIB). **Mun. Totontepec Villa de Morelos**, 1,300 m, 7 Dec 1989, *E. Vargas-Ruíz 476* (MEXU); 1,900 m, 21 Apr 1986, *J. Rivera-Reyes & Gary J. Martin 246* (MEXU); 1,700 m, 15 Jul 1987, *E. Velasco-López & G.J. Martin 60* (MEXU); 1,900 m, 6 Jan 1988, *J. Rivera-Reyes & G.J. Martin 920* (MEXU); 2,000 m, 16 Sep 1987, *W. Ruíz S. 46* (MEXU).

#### ﻿22. Ceratozamia morettii

Taxon classificationPlantaeCycadalesZamiaceae

Vázq.Torres & Vovides, Novon 8 (1): 87. 1998

122290CE-5CE3-5A3D-B71C-802B069282A4

Figs 7I–J , 10A, C

##### Type.

Mexico. Veracruz: Mun. Landero y Coss, El Raicero, 4 km NE from Landero y Coss, 1,500 m, 7 Jan 1992, *M. Vázquez-Torres & H. Barney 4097* (holotype: CIB! [acc. # 22297UV]; isotypes: CIB! [acc. # 24578UV], MEXU [n.v.], XAL! [XAL0001061, XAL0001064])

##### Description.

***Stem*** 20–23 (50) cm long, 20–35 cm in diameter, epigeous, erect and decumbent. ***Cataphylls*** 4–6.5 × 3–3.5 cm wide at the base, persistent, triangular, reddish brown, densely brown tomentose at emergence, partially tomentose at maturity, apex acuminate. ***Leaves*** 3–30, 82–200 cm long, descending, light green at emergence, with brown trichomes, glabrous at maturity. ***Petiole*** 30–90 cm long, terete, linear, green in mature leaves; with 8–60 thin prickles, 0.01–0.20 cm long. ***Rachis*** 50–116 cm long, terete, linear, green in mature leaves, with prickles. ***Leaflets*** 10–23 pairs, opposite to subopposite, oblong, longitudinally curved abaxially to planar, not basally falcate, coriaceous, green with adaxial side glabrous and abaxial side glaucous, distal end with entire margins, acuminate and asymmetrical at the apex, attenuate at base, and with conspicuous and green-light veins; median leaflets 17.5–41 × 2.6–4.3 cm wide, 2.1–6 cm between leaflets; articulations 0.5–1.6 cm wide, green and yellow. ***Pollen strobili*** 12–22 (35.5) cm long, 3.8–5.2 cm in diameter, solitary, cylindrical, erect, brownish yellow at emergence, greenish yellow with blackish pubescent at maturity; peduncle 3–7 (12.5) cm long, 1.1–1.6 cm in diameter, tomentose, reddish brown to brown; microsporophylls 1.0–2.0 × 0.9–1.15 cm, obconic with a non-recurved distal face and a lobate fertile portion, infertile portion 0.37–0.75 cm long and orbicular with straight horns 0.15–0.53 cm long, 0.47–0.92 cm and an acute angle between the horns. ***Ovulate strobili*** 14–20 cm long, 8–9.9 cm in diameter, solitary, globose, erect, yellowish green with brown trichomes at emergence, green with blackish trichomes at maturity and with apiculate apex; peduncle 4–7 cm long, 1.2–1.8 cm in diameter, erect, tomentose, brown; megasporophylls 35–81 (108), 7–10 orthostichies with 5–9 sporophylls per orthostichy, 1.7–2.6 × 3.0–4.5 cm, with a truncate distal face, horns straight and 0.32–0.69 cm long, 1.05–1.6 cm between horns and a right angle between the horns. ***Seeds*** 1.5–2.3 cm, 1–1.6 cm in diameter, ovate and globose, sarcotesta whitish yellow to yellow when immature, light brown at maturity.

##### Distribution and habitat.

*Ceratozamiamorettii* is endemic to the Sierra de Chiconquiaco in Veracruz State, Mexico (Fig. 21B), here it occurs in cloud forest on karstic rocks and cliffs between 1,150–1,850 m.

##### Etymology.

The specific epithet honors the Italian botantist Aldo Moretti from the Orto Botanico and Istituto di Biologia Vegetale at the University of Naples Federico II, in recognition of his scientific contributions to cycad biology (Vázquez-Torres et al. 1998).

##### Common names.

Mexico. Veracruz: Palmita (J. Rees & A.P. Vovides 1663), palmilla (J. Rees & A.P. Vovides 1676).

##### Uses.

Leaves are used in flower arrangements to decorate the homes during religious rites (Fig. 28A).

##### Conservation status.

(IUCN 2021). *Ceratozamiamorettii* has been listed as “Endangered” under criteria B1ab(i,iv,v)+2ab(i,iv,v).

##### Discussion.

In leaf morphology, *Ceratozamiamorettii* is morphologically similar to *C.delucana*; however, there are differences in the total size of the plant and in its reproductive structures. The microsporophyll horns of *C.morettii* form an acute angle, whereas those of *C.delucana* form a right angle. The ovulate strobilus in *C.morettii* is green with blackish trichomes at maturity and an apiculate apex. In contrast, in *C.delucana*, the ovulate strobilus is green and glabrous at maturity with an acute apex.

##### Specimens examined.

Mexico. **Veracruz: Mun. Chiconquiaco**, 1,700 m, 5 Sep 1981, *A.P. Vovides 687* (XAL); 1,850 m, 18 Dec 1981, *A.P. Vovides 704* (XAL); Sep 1829, *C.J.W. Schiede s/n* (XAL); 1,598 m, 17 Sep 2020, *F. Nicolalde-Morejón et al. 3318*–*3322* (CIB); 26 Nov 1974, *J. Rees 6336* (IEB); 1,700 m, 5 Jun 2018, *L. Lagunes G. 83, 84, 85, 86* (CIB); 1,600 m, 26 Apr 2018, *L. Martínez-Domínguez et al. 1280*–*1290* (CIB); 1,700 m, 3 Dec 2018, *L. Martínez-Domínguez et al. 1567, 1568* (CIB); 1,500 m, 15 Mar 2019, *L. Martínez-Domínguez et al. 1660, 1661* (CIB); 1,550 m, 26 Mar 2019, *L. Martínez-Domínguez et al. 1680, 1681* (CIB); 1,598 m, 17 Sep 2020, *L. Martínez-Domínguez et al. 1931–1934, 1936* (CIB), *1935* (CIB, MEXU). **Mun. Landero y Coss**, 1,750 m, 1 Jan 1976, *J. Rees & A.P. Vovides 1662*–*1664* (XAL); 1,750 m, 23 Sep 1976, *J. Rees & A.P. Vovides 1676* (XAL); 1,830 m, 3 Mar 2011, *L.H. Bojórquez G. et al. 23101–2316* (CIB); 1,472 m, 29 Jan 2015, *L. Martínez-Domínguez et al. 185*–*214* (CIB); 1,765 M, 22 Apr 2008, *M. Vázquez-Torres et al. 8349* (CIB); 3 Mar 2001, *S. Avendaño 5378* (MEXU); 1,520 m, 10 Jan 2001, *T.W. Walters 2001-01-E* (XAL). **Mun. Tenochtitlán**, 1,500 m, 10 Apr 2002, *A. Rincón G. et al. 2996, 2997* (XAL); 1,500 m, 12 Apr 2002, *A. Rincón G. et al. 2998* (XAL). **Mun. Yecuatla**, 1,400 m, 26 May 1981, *C. Gutiérrez B. & A. Montoya L. 134* (MO, XAL); 1,211 m, 28 Sep 2014, *F. Nicolalde-Morejón & L. Martínez-Domínguez 2087*–*2106* (CIB); 1, 200 m, 26 Sep 1976, *J. Rees 1677* (XAL); 1,211 m, 9 Aug 2014, *L. Martínez-Domínguez et al. 161* (CIB).

#### ﻿23. Ceratozamia norstogii

Taxon classificationPlantaeCycadalesZamiaceae

D.W.Stev., Brittonia 34: 181. 1982

9CBD0D5C-5572-52D3-953F-80AA0BABEA34

Fig. 27A

##### Type.

Mexico. Chiapas: Rancho Fenia, Mar-Apr 1925, *C. Purpus 6* ♀ (holotype: NY! [00001116]; isotypes: F! [acc. # 1530231], MO!, US! [00620294]).

##### Description.

***Stem*** 30–60 cm long, 20–40 cm in diameter, epigeous, erect and decumbent. ***Cataphylls*** 4.5–6 × 2.5–6.4 cm wide at the base, persistent, long triangular, reddish brown, densely brown tomentose at emergence, tomentose at maturity, apex acuminate. ***Leaves*** 5–48, 50–126 cm long, ascending, copperish brown at emergence with whitish gray trichomes, glabrous at maturity. ***Petiole*** 10–49 cm long, terete, twisted, copperish with abundant greyish trichomes in young leaves, copperish and glabrous in mature leaves; with 30–60 robust prickles, 0.11–0.31 cm long. ***Rachis*** 40–88 cm long, terete, twisted, copperish with abundant greyish trichomes in young leaves, copperish and glabrous in mature leaves, with prickles. ***Leaflets*** 33–91 pairs, opposite to subopposite, insertion irregular to in one plane, linear, in general longitudinally planar, not basally falcate to basally falcate, coriaceous, strongly involute to caniculate, green with yellowish green base and with adaxial side glabrous and abaxial side glaucous, distal end with entire margins, acute and symmetric at the apex, attenuate at base, with conspicuous and light-green veins; median leaflets 14–48 × 0.21–0.46 cm, 0.2–1.3 cm between leaflets; articulations 0.08–0.66 cm wide, yellowish. ***Pollen strobili*** 13.2–25 cm long, 3.1–8 cm in diameter, solitary, cylindrical, erect, brown with reddish brown trichomes at emergence, yellowish green to cream at maturity; peduncle 2.0–5.5 cm long, 1.3–2.3 cm in diameter, tomentose, brown; microsporophylls 0.92–1.16 × 0.86–0.1.1 cm, discoid with a non-recurved distal face and a lobate fertile portion, infertile portion 0.36–0.45 cm long and orbicular with straight horns 0.07–0.13 cm long, 0.35–0.56 cm and an acute angle between the horns. ***Ovulate strobili*** 21–35 cm long, 8.5–10.5 cm in diameter, solitary, cylindrical, erect, green with abundant blackish trichomes at emergence, grayish green with abundant blackish trichomes at maturity, acuminate apex; peduncle 4.8–9 cm long, 1.8–2.2 cm in diameter, erect, tomentose, brown; megasporophylls 42–63, 6–7 orthostichies with 7–9 sporophylls per orthostichy, 2.6–3.0 × 3.6–4.0 cm, with a truncated distal face, horns straight and 0.37–0.58 cm long, 0.37–0.42 cm between horns with a right angle between the horns. ***Seeds*** 2.0–3.5 cm long, 1.1–1.9 cm in diameter ovate, sarcotesta whitish pink when immature, light brown at maturity.

##### Distribution and habitat.

*Ceratozamianorstogii* is distributed in mountainous regions in Chiapas and Oaxaca States, Mexico (Fig. 21C), where it occurs on karstic rocks in pine-oak forest and the transition between pine forest and oak forest between 800–1,650 m.

##### Etymology.

This species was named in honor of Knut Norstog, for his extensive and significant contributions to cycad biology (Stevenson 1982).

##### Common names.

None recorded.

##### Uses.

None recorded.

##### Conservation status.

(IUCN 2021). The currect category of threat to *Ceratozamianorstogii* is “Endangered” under criteria A2abd; B1ab(iii,iv,v); C1.

##### Discussion.

*Ceratozamianorstogii* is easily diagnosable by its petiole and raquis twisted. The linear leaflets are coriaceous, strongly involute and appear caniculate and have an acute apex.

##### Specimens examined.

Mexico. **Chiapas: Mun. Cintalapa**, 1,100 m, 19 Mar 1993, *A.P. Vovides et al. 1230, 1231, 1233, 1235* (XAL); 1,600 m, 3 Nov 1971, *D.E. Breedlove 21813* (MEXU, MO); 1,600 m, 21 Apr 1972, *D.E. Breedlove 24709* (MO); 1,600 m, 12 Oct 1979, *D.E. Breedlove 44431* (MEXU); 800 m, 4 May 1988, *E. Palacios E. 375* (CHIP, IBUG); 1,240 m, 6 Jun 1993, *E. Palacios E. 2155* (CHIP); 1,280 m, 22 May 2001, *J.M. Lázaro-Zermeño 251* (CHIP); 1,038 m, 20 Jun 2018, *F. Nicolalde-Morejón et al. 2762–2770* (CIB); 1,325 m, 20 Jun 2018, *F. Nicolalde-Morejón et al. 2771–2780* (CIB); 1,038 m, 20 Jun 2018, *L. Martínez-Domínguez et al. 1326*–*1334* (CIB), *1335* (CIB, MEXU); 1,325 m, 20 Jun 2018, *L. Martínez-Domínguez et al. 1337*–*1349* (CIB); 1,100 m, 5 Oct 1995, *M.A. Pérez-Farrera 775* (CH, CIB, XAL); *5 Dec 1996, M.A. Pérez-Farrera 1483* (HEM). **Oaxaca: Mun. San Miguel Chimalapa**, 1,120 m, 1 Apr 1996, *S. Salas-Morales et al. 1173* (SERO, XAL). **Mun. Santo Domingo Zanatepec**, 800 m, 22 Jun 2018, *F. Nicolalde-Morejón et al. 2819–2828* (CIB); 800 m, 22 Jun 2018, *L. Martínez-Domínguez et al. 1380, 1381, 1383, 1386, 1389–1391, 1394, 1396, 1399* (CIB).

#### ﻿24. Ceratozamia oliversacksii

Taxon classificationPlantaeCycadalesZamiaceae

D.W.Stev., Mart.-Domínguez & Nic.-Mor., Kew Bull. 77: 212. 2022

2644CF65-A3C5-560F-907C-3AA4FF9B52EF

Fig. 27B

##### Type.

Mexico. Oaxaca: Mun. Candelaria Loxicha, Cerro Perico, 1,616 m, 10 Jun 2021, *L. Martínez-Domínguez et al. 2261* ♀ (holotype: CIB! [acc. # 23411UV]; isotypes: K!, MEXU!, NY!).

##### Description.

***Stem*** 30–80 cm long, 10–40 cm in diameter, epigeous, decumbent. ***Cataphylls*** 6.5–7.5 × 1.6–2.0 cm wide at the base, persistent, triangular, reddish brown, brown tomentose at emergence, glabrous at maturity, apex acuminate. ***Leaves*** 7–36 (50), 124–258 cm long, descending, green at emergence with brown trichomes, glabrous at maturity. ***Petiole*** 45–110 cm long, terete, linear, green at emergence, dark green in mature leaves; with 40–65 thin prickles, 0.50–0.70 cm long. ***Rachis*** 85–182 cm long, terete, linear, dark green in mature leaves with prickles. ***Leaflets*** 24–47 pairs, opposite to subopposite, insertion in one plane, linear, longitudinally curved abaxially to planar, not basally falcate, papyraceous, flat, green with adaxial and abaxial sides glabrous, distal end with entire margins, acuminate and symmetrical (rarely asymmetrical) at the apex, attenuate at base, with conspicuous and light-green veins; median leaflets 25–40 × 2.3–3.6 cm, 2.0–3.3 cm between leaflets; articulations 0.5–1.1 cm wide, green. ***Pollen strobili*** 20–35 cm long, 3.0–5.7 cm in diameter, solitary, cylindrical, erect, greenish with reddish trichomes at emergence, greenish yellow with reddish brown trichomes at maturity; peduncle 10–15 cm long, 1.2–1.8 cm in diameter, pubescent, reddish brown; microsporophylls 2.5–2.75 × 0.99–1.28 cm, obconic with a non-recurved distal face and a lobate fertile portion, infertile portion 0.60–0.80 cm long and a rounded with straight horns 0.08–0.18 cm long, 0.50–0.70 cm and an acute angle between the horns. ***Ovulate strobili*** 33–36 cm long, 11.5–14 cm in diameter, solitary, cylindrical, erect, greenish yellow with few reddish trichomes at emergence, green with brown to reddish brown trichomes or glabrous at maturity, acute apex; peduncle 8.0–10.0 cm long, 1.4–1.6 cm in diameter, erect, pubescent, light brown; megasporophylls 80–210, 9–10 orthostichies with 11–13 sporophylls per orthostichy, 2.3–2.9 × 3.9–5.0 cm, with a truncate distal face, horns straight and 0.31–0.53 cm long, 1.33–1.40 cm between horns with a right angle between the horns. ***Seeds*** 2.45–2.65 cm long, 1.4–1.6 cm in diameter, ovate, sarcotesta whitish yellow to yellow when immature, light brown at maturity.

##### Distribution and habitat.

*Ceratozamiaoliversacksii* occurs along the Eastern Sierra Madre del Sur in the Oaxacan Highlands, Mexico (Fig. 21D), it is found on karstic rocks with abundant organic matter in cloud forest and the transition zone between it and pine forest between 1,040 to 1,850 m.

##### Etymology.

The specific epithet honors Oliver Sacks, who loved cycads and was a distinguished American neurologist and historian of science. Sacks published Island of the Color Blind and Cycad Island (Sacks 1997) and Oaxaca Journal (Sacks 2002) (Martínez-Domínguez et al. 2022b).

##### Common names.

None recorded.

##### Uses.

None recorded.

##### Preliminary conservation status.

*Ceratozamiaoliversacksii* should be listed as “Vulnerable (V)”. We visited four populations in Oaxaca in which each population size was between 50 to 300 adult plants. We observed different age classes from seedling to reproductive individuals.

##### Discussion.

*Ceratozamiaoliversacksii* is similar to *C.robusta* and *C.leptoceras* but is characterized by green leaves at emergence and the combination of linear and papyraceous leaflets. The ovulate strobili have 11 to 13 sporophylls per orthostichies and an acute apex, acute horns of megasporophylls up to 0.53 cm long, and microsporophylls with a rounded distal face and straight horns.

##### Specimens examined.

Mexico. **Oaxaca: Mun. Candelaria Loxicha**, 1,380 m, 3 May 2005, *A. Luna-José & B. Rendón-Aguilar 1472, 1473* (XAL); 1,630 m, 23 Jun 2019, *F. Nicolalde-Morejón & L. Martínez-Domínguez 3231–3243* (CIB); 1,630 m, 23 Jun 2019, *L. Martínez-Domínguez & F. Nicolalde-Morejón 1832–1839* (CIB), *1840* (CIB, MEXU); 1,418 m, 20 April 2021, *L. Martínez-Domínguez et al. 2160, 2161* (CIB); 1,616 m, 10 Jun 2021, *M. Rios-Méndez et al. 105* (CIB). **Mun. San Agustín Loxicha**, 1,760 m, 12 Oct 2003, *A. Luna-José & B. Rendón-Aguilar* 5*18* (XAL); 1,400 m, 23 Jun 2019, *F. Nicolalde-Morejón & L. Martínez-Domínguez 3229, 3230* (CIB); 1,400 m, 23 Jun 2019, *L. Martínez-Domínguez & F. Nicolalde-Morejón 1829–1831* (CIB). **Mun. Santa Catarina Juquila**, 1,850 m, 13 Apr 1965, *J. Rzedowski* 1*9557* (ENCB); 1,450–1,700 m, 11 Feb 1965, *R. McVaugh 22346* (ENCB; MICH). **Mun. San Miguel del Puerto**, 1,060 m, 25 Jun 2019, *F. Nicolalde-Morejón & L. Martínez-Domínguez 3244–3249* (CIB);1,430 m, 17 Apr 2000, *J. Rivera H. et al. 2378* (FCME, SERO); 1,060 m, 25 Jun 2019, *L. Martínez-Domínguez & F. Nicolalde-Morejón 1847–1849, 1851, 1852* (CIB), *1850* (CIB, MEXU); 1,040 m, 1 Apr 2003, *L. Schibli et al. 152* (SERO).

#### ﻿25. Ceratozamia osbornei

Taxon classificationPlantaeCycadalesZamiaceae

D.W.Stev., Mart.-Domínguez & Nic.-Mor. sp. nov.

FEC37F52-3E7B-59B2-818B-8BA674999F43

urn:lsid:ipni.org:names:77305494-1

Figs 5 , 6

##### Diagnosis.

Similar to *Ceratozamiarobusta* and *C.subroseophylla*, but distinguished by characters of reproductive structures. It has megasporophylls with abundant purple to wine-colored trichomes, ovulate strobili with an acuminate apex and up to 12 orthostichies with 31 sporophylls per orthostichy and pollen strobili with a long infertile portion from 0.65 to 0.80 cm long.

##### Type.

BELIZE. Toledo: Southwestern Maya Mountains, Columbia River Forest Reserve, Union Camp. 6 Apr 1992, *B.K. Holst 4105* (holotype: NY! [01340569]; isotype: MO! [acc. # 04661737, 04661738]).

##### Description.

***Stem*** 30–200 cm long, 20–40 cm in diameter, epigeous, generally decumbent. ***Cataphylls*** 5.5–8 × 2–3 cm wide at the base, persistent, triangular, reddish brown, brown tomentose at emergence, glabrous at maturity, apex acuminate. ***Leaves*** 12–50, 90–300 cm long, ascending, brown at emergence, with whitish gray trichomes, glabrous at maturity. ***Petiole*** 75–100 cm long, terete, linear, greenish brown or dark brown at emergence, green in mature leaves; with 20–40 robust prickles, 0.20–0.45 cm long. ***Rachis*** 90–200 cm long, terete, linear, greenish brown or dark brown at emergence, green in mature leaves, with prickles. ***Leaflets*** 26–51 pairs, opposite to subopposite, insertion in one plane, lanceolate, longitudinally curved abaxially to planar, not basally falcate, papyraceous, flat, dark green, adaxial side glabrous, abaxial side glabrous, distal end with entire margins, acuminate and symmetric at the apex, attenuate at base, with conspicuous and light-green veins; median leaflets 30–45 × 2.5–4.0 cm, 1.7–5 cm between leaflets; articulations 0.9–1.6 cm wide, brown in young leaves and green in mature leaves. ***Pollen strobili*** 60–80 cm long, 7–9 cm in diameter, solitary, cylindrical, erect, green with wine trichomes at emergence, greenish yellow at maturity with wine to purple trichomes; peduncle 7–11 cm long, 2.5–3.5 cm in diameter, tomentose, brown; microsporophylls 2.0–3.3 × 1.1–1.85 cm, obconic with a non-recurved distal face and deeply lobate fertile portion, infertile portion 0.65–0.80 cm long and rounded with straight horns 0.20–0.29 cm long, 0.55–0.75 cm and a right angle between the horns. ***Ovulate strobili*** 30–50 cm long, 9–14 cm in diameter, solitary, cylindrical, erect, green with abundant wine to dark purple trichomes at emergence, green with abundant dark purple trichomes at maturity, acuminate apex; peduncle 12–20 cm long, 2.5–4.5 cm in diameter, erect, tomentose, reddish brown; megasporophylls 108–280, 9–12 orthostichies with 12–31 sporophylls per orthostichy, 1.8–2.3 × 2.3–4.1 cm, with a prominent distal face, horns straight and 0.36–0.50 cm long, 0.90–1.35 cm between horns with a right angle between the horns. ***Seeds*** 3.0–4.5 cm long, 2.0–3.0 cm in diameter, ovate, sarcotesta whitish pink when immature, light brown at maturity.

##### Distribution and habitat.

**Figure 22.**
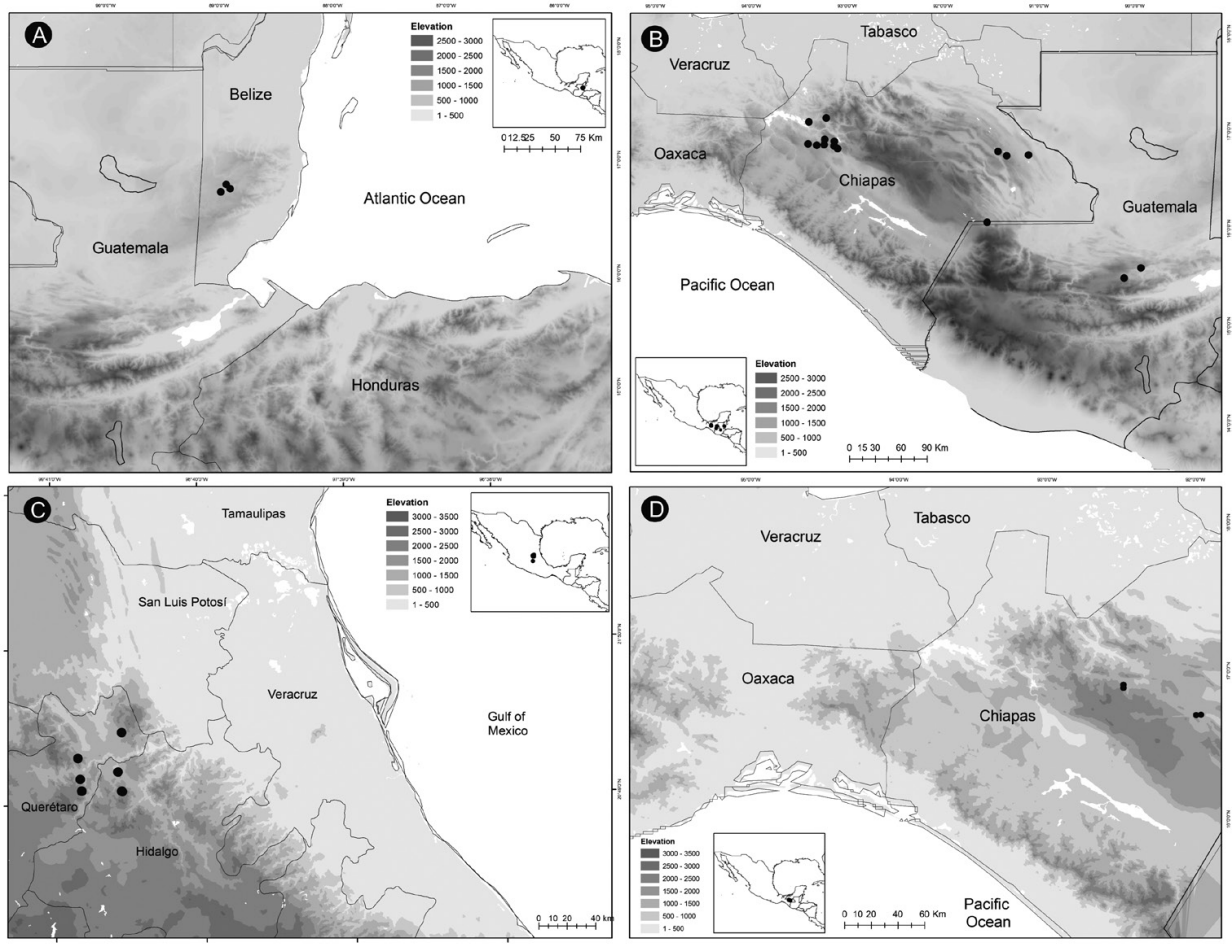
Distribution of *Ceratozamia* species. **A***C.osbornei***B***C.robusta***C***C.sabatoi***D***C.sancheziae*.

*Ceratozamiaosbornei* is endemic to Belize (Fig. 22A), where it occurs in evergreen tropical forest on karstic rocks with abundant organic matter between 200 and 750 m elevation.

##### Etymology.

The specific epithet honors Roy Osborne in recognition of his remarkable contributions to the knowledge of the cycad diversity in the world. In particular, he has actively participated in the project “The World List of Cycads” since its inception (https://www.cycadlist.org).

##### Common names.

None recorded.

##### Uses.

None recorded.

##### Conservation status.

There is insufficient data for evaluation, but it is known that some populations have more than 100 plants.

##### Paratypes.

BELIZE. **Cayo**: 19 Aug 2008, *M. Calonje et al*. *BZ08-042* (FTG); 20 Aug 2008, *M. Calonje et al*. *BZ08-056* (FTG); 25 Feb 1992, *C. Hubbuch et al.* (FTG); 6 Apr 1971, *S. Kiem s.n.* [027932] (FTG); Spring, 1973, *S. Kiem s.n.* [027929] (FTG); 550 m, 4 Aug 1980, *Sutton et al. 15* (MEXU, MO); 455 m, 12 Dec 1996, *T. Hawkins 1186* (NY, MO). **Toledo**: 750 m, 15 May 1979, *C. Whitefoord 1764* (BM); 800–1,200 m, 23–27 Apr 1976, *G.R. Proctor 35985* (BM, MO).

#### ﻿26. Ceratozamia robusta

Taxon classificationPlantaeCycadalesZamiaceae

Miq., Tijdschr. Wis-Natuurk. Wetensch. Eerste Kl. Kon. Ned. Inst. Wetensch. 1: 42. 1847

41F0AD37-EA8F-5A45-B427-0E4722B73AC4

Fig. 27C

Ceratozamia mexicana Brongn. var. robusta (Miq.) Dyer, Biol. Cent.-Amer., Bot. 3: 193. 1884. Type. Based on: Ceratozamiarobusta Miq.

##### Type.

Mexico. Chiapas: NW of Tuxtla Gutiérrez on road through San Fernando 23.5 km from route 190, 1,000 m, 9 Jul 1983, *D.W. Stevenson 549A* (neotype, designated by Stevenson and Sabato 1986, pg. 581: NY! [00001119]).

##### Description.

***Stem*** 30–100 cm long, 20–40 cm in diameter, epigeous, erect and decumbent. ***Cataphylls*** 5.0–9.0 × 3.5–7.5 cm wide at the base, persistent, long triangular, reddish brown, densely brown tomentose at emergence, partially tomentose at maturity, apex acuminate. ***Leaves*** 10–50, 138–266 cm long, ascending, dark brown at emergence, green and glabrous at maturity. ***Petiole*** 56–85 cm long, terete, linear, brown in young leaves, green in mature leaves; with 27–55 robust prickles, 0.48–0.70 cm long. ***Rachis*** 60–190 cm long, terete, linear, brown in young leaves, green in mature leaves, with prickles. ***Leaflets*** 20–45 pairs, opposite to subopposite, insertion in one plane, lanceolate, in general longitudinally planar, not basally falcate, papyraceous, flat, green with adaxial side glabrous and abaxial side glaucous, distal end with entire margins, acuminate and asymmetrical at the apex, attenuate at base, with conspicuous and light-green veins; median leaflets 28–42 × 2.5–4.0 cm, 3.0–4.5 cm between leaflets; articulations 0.2–0.8 cm wide, green. ***Pollen strobili*** 50–70 cm long, 7.0–8.5 cm in diameter, solitary, cylindrical, erect, light green to yellowish green with blackish trichomes at emergence, yellowish cream with reddish brown trichomes at maturity; peduncle 10–15 cm long, 2.5–3.2 cm in diameter, tomentose, reddish brown to brown; microsporophylls 2.33–3.0 × 1.14–1.90 cm, obconic with a non-recurved distal face and deeply lobate fertile portion, infertile portion 0.45–0.65 cm long and rounded with curved horns 0.26–0.40 cm long, 0.55–0.75 cm and a right angle between the horns. ***Ovulate strobili*** 26–40 cm long, 11–14.5 cm in diameter, solitary, cylindrical, erect, dark green at emergence, dark green with scarce blackish trichomes at maturity, acuminate apex; peduncle 5.0–11.0 cm long, 2.5–4.0 cm in diameter, erect, tomentose, reddish brown; megasporophylls 153–280, 8–14 orthostichies with 17–20 sporophylls per orthostichy, 1.7–2.5 × 2.3–3.5 cm, with a prominent distal face, horns straight and 0.38–0.50 cm long, 1.35–1.60 cm between horns with a right angle between the horns. ***Seeds*** 2.5–3.5 cm long, 1.9–2.5 cm in diameter, ovate, sarcotesta whitish yellow to yellow when immature, light brown at maturity.

##### Distribution and habitat.

*Ceratozamiarobusta* is widely distributed in Chiapas State, Mexico and northern Guatemala (Fig. 22B). It occurs in evergreen tropical forest between 400 to 1,300 m on karstic rocks with humus soil.

##### Etymology.

The epithet is derived from the robust appearance of the species.

##### Common names.

Mexico. Chiapas: Amendu (M.A. Pérez-Farrera 30), piña del tlacoache (A.P. Vovides & J.I. Calzada 481).

##### Uses.

The seeds are used as food, and the leaves to decorate altars.

##### Conservation status.

(IUCN 2021). *Ceratozamiarobusta* is listed as “Endangered” under criteria A2acd. This species has a wide distribution, but some populations previously considered to be part of this entity have been assigned to described species durint the last ten years. Therefore, a reassessment of its conservation status is required.

##### Discussion.

*Ceratozamiarobusta* is similar to *C.subroseophylla* and distinguished by its dark green ovulate strobilus with sparse blackish trichomes on megasporophylls at maturity, prominent distal face, straight horns with a right angle between them, differentiating it easily from *C.subroseophylla*. The pollen strobilus of *C.robusta* is the largest in the genus (up to 70 cm long).

##### Specimens examined.

Guatemala. **Alta Verapaz**: 200 m, 13 Jul 2002, *S. Hernández 757* (MO); 1,800-2,000 m, 27 Feb 1939, *C.L. Wilson 262* (F); 300-500 m, 17 Apr 1942, *J.A. Steyermark 45734* (US); 879 m, 15 Mar 2008, *M. Véliz et al. 19998* (BIGU). **Huehuetenango**: 2,000 m, 10 Sep 1942, *J.A. Steyermark* 52046 (NY, US); 1,150 m, 26 Jul 1943, *J.A. Steyermark 49506* (F; MO; NY); 400 m, 27 Jul 1942, *J.A. Steyermark 49682* (NY); 900-1,300 m, 3 Sep 1942, *J.A. Steyermark* 51818 (F; NY; US); 900-1,000 m, 6 Mar 2009, *M.J.M. Christenhusz et al. 5600* (MO); 1,161 m, 21 Dec 2010, *L. Vélasquez et al. 1566* (BIGU). Mexico**. Chiapas: Mun. Berriozábal**, 1,129 m, 6 Mar 2014, *F. Nicolalde-Morejón et al. 1970–1975* (CIB); 1,129 m, 6 Mar 2014, *L. Martínez-Domínguez et al. 41–47, 49, 50* (CIB), *48* (CIB, MEXU). **Mun. Copainalá**, 1,100 m, *M. Gutiérrez & T. Acero 240* (CHIP). **Mun. Ocosingo**, 900 m, 16 Mar 1981, *A.P. Vovides & J.I. Calzada 481* (XAL); 14 Apr 1967, *D.E. Breedlove 15687* (ENCB); 700 m, 18 Apr 1985, *E. Martínez S. 12067* (MEXU); 860 m, 24 Feb 1984, *J. García F. 720* (CH, CIB, XAL). **Mun. Ocozocoautla de Espinosa**, 818 m, 11 Nov 1997, *R. Álvarez G. 6* (CHIP). **Mun. Osumacinta**, 692, 18 Jul 2008, *R. Gallegos Ramos 211* (CHIP). **Mun. San Fernando**, 680 m, 29 Apr 1995, *A.P. Vovides & M.A. Pérez-Farrera 1266ª* (XAL); 22 Sep 1993, *Brigada T. Walters s/n* (XAL); 9 Jul 1983, *D.W. Stevenson et al. 549 B*–*F* (NY); 1,000 m, 9 Jul 1983, *D.W. Stevenson et al. 550 A–C* (NY), 24 Apr 1999, *M.A. Isidro V. 388* (CHIP); 29 Apr 1995, *M.A. Pérez-Farrera 293* (MEXU); 12 Jun 2009, *N. Martínez-Meléndez 2692* (MEXU); 1,200 m, 5 Dec 2000, *R.A. Palestina & I. Acosta 2707* (XAL). **Mun. Tuxtla Gutiérrez**, 1,235 m, 19 Mar 1994, *M.A. Pérez-Farrera 30* (CH, MEXU, XAL); 1,200 m, 1 Nov 1995, *M.A. Pérez-Farrera 820* (CHIP).

#### ﻿27. Ceratozamia sabatoi

Taxon classificationPlantaeCycadalesZamiaceae

Vovides, Vázq.Torres, Schutzman & Iglesias, Novon 3 (4): 502. 1993

9879DB96-4EA4-5F99-BF71-71F4AF270807

Figs 1A , 27D

##### Type.

Mexico. Querétaro: Mun. San Joaquín, La Mojonera, 2 km on road San Joaquín-El Aguacate, 1,850 m, 15 Apr 1991, *A.P. Vovides & P. Fawcett 1205* ♀ (holotype: XAL! [XAL0005310]).

##### Description.

***Stem*** 8–30 cm long, 20–35 cm in diameter, epigeous, erect and decumbent. ***Cataphylls*** 3–4.5 × 2–3.5 cm wide at the base, persistent, triangular, reddish brown, densely brown tomentose at emergence, partially tomentose at maturity, apex acuminate. ***Leaves*** 3–40, 60–129 cm long, descending, dark brown at emergence with whitish gray trichomes, glabrous at maturity. ***Petiole*** 20–60 cm long, terete, linear, greenish brown in mature leaves; with 5–40 thin prickles, 0.02–0.19 cm long. ***Rachis*** 40–92 cm long, terete, linear, brown in mature leaves, with prickles. ***Leaflets*** 26–54 pairs, opposite to subopposite, insertion in one plane, linear, longitudinally curved abaxially to planar, basally falcate, papyraceous, flat, green with adaxial and abaxial sides glabrous, distal end with entire margins, acuminate and symmetrical at the apex, attenuate at base, with conspicuous and green-light veins; median leaflets 13–32 × 0.6–1.5 cm, 0.5–1.5 cm between leaflets; articulations 0.3–0.7 cm wide, brown. ***Pollen strobili*** 11–18 cm long, 3.5–4.8 cm in diameter, solitary, cylindrical, erect, greenish yellow at emergence, greenish yellow with blackish trichomes at maturity; peduncle 7–13 cm long, 1.1–1.9 cm in diameter, tomentose, reddish brown to brown; microsporophylls 1.0–1.9 × 0.85–1.90 cm, discoid with a recurved downward distal face and lobate fertile portion, infertile portion 0.33–0.43 cm long and rounded with straight horns 0.06–0.20 cm long, 0.30–0.71 cm and a right angle between the horns. ***Ovulate strobili*** 14–19.5 cm long, 5.5–8.5 cm in diameter, solitary, cylindrical, erect, yellowish green with brown trichomes at emergence, blue green with blackish trichomes at maturity, apiculate apex; peduncle 3.5–7 cm long, 1.2–2.5 cm in diameter, erect, tomentose, brown; megasporophylls 72–110, 8–10 orthostichies with 9–11 sporophylls per orthostichy, 3.5–5 × 3.8–4.5 cm, with a truncate distal face, horns straight and 0.28–0.40 cm long, 1.2–1.6 cm between horns with a right angle between the horns. ***Seeds*** 1.2–2.0 cm long, 1.2–1.5 cm in diameter, ovate, sarcotesta whitish red when immature, light brown at maturity.

##### Distribution and habitat.

*Ceratozamiasabatoi* is endemic to the Sierra Gorda mountain range in Mexico, particularly in Querétaro and Hidalgo States (Fig. 22C), where it occurs in the understory herbaceous layer of the transition zone between oak forest and cloud forest at 1,600–1,900 m.

##### Etymology.

The specific epithet honors Sergio Sabato, a distinguished professor at the University of Naples Federico II for his outstanding contributions to knowledge of the biology of Zamiaceae (Vovides et al. 1993).

##### Common names.

None recorded.

##### Uses.

None recorded.

##### Conservation status.

(IUCN 2021). *Ceratozamiasabatoi* is listed as “Endangered” under criteria A2c; B1ab(i,ii,iv)+2ab(i,ii,iv); C1.

##### Discussion.

*Ceratozamiasabatoi* differs from all *Ceratozamia* species by its microsporophylls with a recurved downward distal face. In addition, this species differs from *C.kuesteriana* by its flat leaflets and descending leaves.

##### Specimens examined.

Mexico. **Hidalgo: Mun. Jacala de Ledezma**, 1,725 m, 16 Aug 2007, *A. Frias-Castro et al. 578* (IBUG); 1,500 m, 29 Oct 1946, *H.E. Moore, Jr. 1788* (CHAPA). **Mun. Zimapán**, 2,000 m, 2 Jun 1989, *M. Vázquez-Torres & J.P. Sclavo 4035* (CIB); 1,235 m, 22 May 2003, *R. Contreras-Medina 55, 56* (XAL); 1,900 m, 13 Sep 1981, *R. Fernandez-Nava 6561* (MEXU, MO, XAL). **Querétaro**: **Mun. Cadereyta de Montes**, 1,850 m, 15 Apr 1991, *A.P. Vovides & K. Norstog 1193* (XAL); *A.P. Vovides et al. 1196*–*1199* (XAL), *1203* (MEXU, XAL); 1,850 m, 15 Apr 1991,); *A.P. Vovides 1201, 1205* (XAL); 1,924 m, 28 Mar 2015, *F. Nicolalde-Morejón et al. 2169, 2170* (CIB); 1,924 m, 28 Mar 2015, *L. Martínez-Domínguez et al. 313*–*343* (CIB); 29 Mar 1995, *R. Fernández-Nava s/n* (MEXU); 7 Dec 1990, *R. Zirahuén-Ortega V. 328* (MEXU). **Mun. Landa de Matamoros**, 1,439 m, 12 Jan 2001, *T.W. Walters 2001-05-A, B* (XAL). **Mun. Pinal de Amoles**, 1,760 m, 17 Sep 2001, *E. Carranza G. & I. Silva 6254* (MO); 1,678 m, 29 Mar 2015, *F. Nicolalde-Morejón et al. 2171, 2172* (CIB); 1,678 m, 29 Mar 2015, *L. Martínez-Domínguez et al. 344*–*372* (CIB); 1,700 m, 11 Dec 1988, *Rzedowski s/n* (XAL); 1,650 m, 4 Apr 1987, *R. Fernández N. 3819* (ENCB).

#### ﻿28. Ceratozamia sancheziae

Taxon classificationPlantaeCycadalesZamiaceae

Pérez-Farr., Gut.Ortega & Vovides, Phytotaxa 500 (3): 209. 2021

D6B2659E-4136-5AE9-845F-17DCED907646

**Figure 23.**
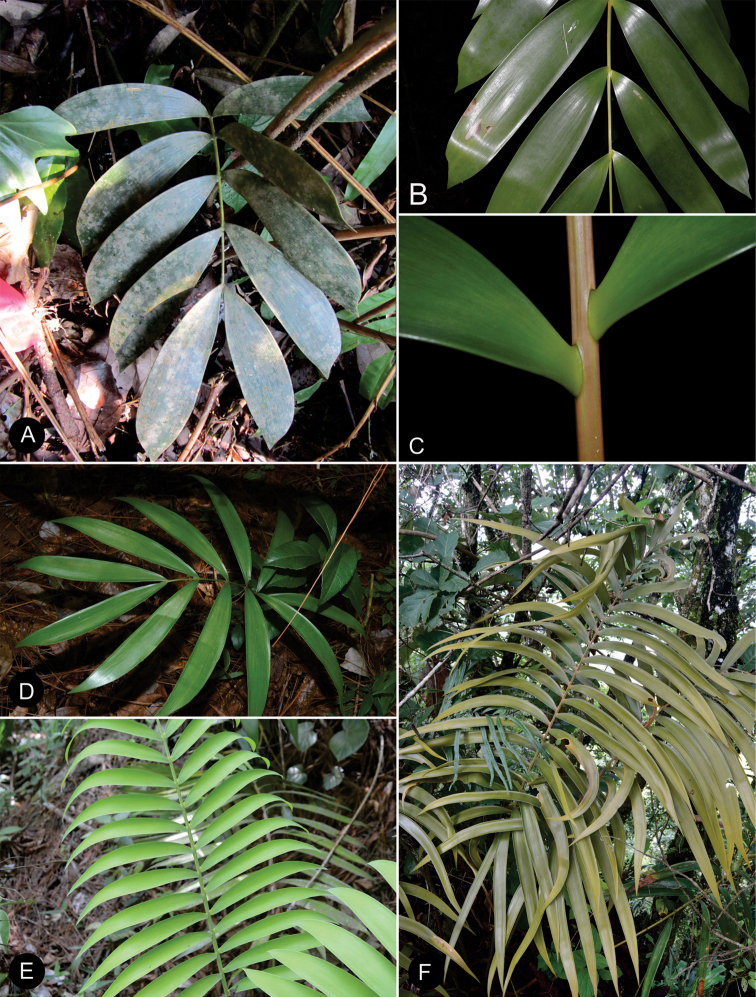
Vegetative comparison between *Ceratozamiazoquorum* (**A–C**) and *C.sancheziae* (**D–F**). **A** juvenile plant **B** mature leaf **C** detail of leaflets and rachis **D** juvenile plant **E, F** leaves at emergence of adult plants.

Figs 9A , 12A , 23 D–F

##### Type.

Mexico. Chiapas: Mun. Tenejapa, 1,500 m, 16 May 2017, *M.A. Pérez Farrera 3558* ♀ (holotype: HEM [n.v.]; isotypes: MEXU [n.v.], XAL [n.v.]).

##### Description.

***Stem*** 8–30 cm long, 10–20 cm in diameter, semi-hypogeous, erect. ***Cataphylls*** 2.5–5.8 × 1.3–5 cm wide at the base, persistent, triangular, reddish brown, densely brown tomentose at emergence, glabrous at maturity, apex acuminate. ***Leaves*** 4–12 (20), 100–232.5 cm long, descending, green or brown at emergence with whitish gray trichomes, glabrous at maturity. ***Petiole*** 40–134.5 cm long, terete, linear, greenish brown or green in mature leaves; unarmed or with prickles 2–28 thin, 0.05–0.34 cm long. ***Rachis*** 55–120 cm long, terete, linear, brown or green in mature leaves, unarmed or with scarce prickles. ***Leaflets*** 20–35 pairs, opposite to subopposite, insertion in one plane, linear, in general longitudinally planar, not basally falcate (sometimes basally falcate), papyraceous, flat, green with adaxial side glabrous and abaxial side glaucous, distal end with entire margins, acuminate and symmetrical at the apex, attenuate at base, with conspicuous and green-light veins; median leaflets 22–36 × 1.7–3.0 cm, 1.4–3.5 cm between leaflets; articulations 0.55–1.1 cm wide, green. ***Pollen strobili*** 15–20 cm long, 1.8–3.2 cm in diameter, solitary, cylindrical, erect, light green at emergence, green with blackish trichomes at maturity; peduncle 6–10 cm long, 0.7–1.5 cm in diameter, tomentose, reddish brown to brown; microsporophylls 0.88–1.2 × 0.65–0.80 cm, discoid with a non-recurved distal face and a lobate fertile portion, infertile portion 0.32–0.40 cm long and rounded with straight horns 0.10–0.20 cm long, 0.63–0.70 cm and an acute angle between the horns. ***Ovulate strobili*** 12–25 cm long, 7.0–8.5 cm in diameter, solitary, cylindrical, erect, yellowish green at emergence, green with scarce blackish trichomes at maturity, acuminate apex; peduncle 5.0–11 cm long, 0.9–1.1 cm in diameter, erect, tomentose, brown; megasporophylls 35–100, 7–10 orthostichies with 5–10 sporophylls per orthostichy, 0.9–1.2 × 3.5–4 cm, with a prominent distal face, horns curved to straight and 0.50–0.73 cm long, 1.2–1.4 cm between horns with a right angle between the horns. ***Seeds*** 1.9–2.3 cm long, 1.4–1.5 cm in diameter, globose, sarcotesta whitish yellow when immature, light brown at maturity.

##### Distribution and habitat.

*Ceratozamiasancheziae* is endemic to Chiapas State, Mexico (Fig. 22D), where it occurs in oak forest and pine-oak forest between 1,000–1,536 m.

##### Etymology.

This species was named in honor of María Ydelia Sánchez-Tinoco, for contributions to our knowledge of the anatomy of Mexican cycad seeds (Gutiérrez-Ortega et al. 2021).

##### Common names.

None recorded.

##### Uses.

The community in Tenejapa use the leaves of this species for ornaments in traditional community festivities.

##### Preliminary conservation status.

*Ceratozamiasancheziae* could be included as “Endangered” (EN) in the IUCN Red List of Threatened Species based on the number of populations, which have between 70 to 250 adult plants.

##### Discussion.

*Ceratozamiasancheziae* is geographically close to *C.robusta*, but differs from it by its linear leaflets, leaves at emergence that are light green or reddish brown with a glaucous appearance, and ovulate strobili with curved to straight horns up to 0.73 cm long.

Here, we have recircumscribed and clarified the taxonomic identity of *Ceratozamiasancheziae*. In the description of this species, the authors mentioned Petalcingo as a municipality, but this is a locality that corresponds to Tila municipality in Chiapas (c.f. Gutiérrez-Ortega et al. 2021). The distribution range for this species was considered to extend from Tenajapa municipality in Chiapas to the mountain area close to the border with the State of Tabasco (Gutiérrez-Ortega et al. 2021). However, we found specimens from Yajalón of Chiapas (*M.A. Pérez Farrera 1635* from XAL) that do not correspond to the species description (Suppl. material 2). We carried out fieldwork and collected botanical material at the population level in the surroundings of Yajalón, Tila, Altamirano and Tenejapa. Based on the revision of herbarium specimens and the botanical material collected by us, we recircumscribed the populations for *Ceratozamia* in this region into two species: *C.sancheziae* and *C.zoquorum*. One diagnostic character for *C.sancheziae* is oblanceolate leaflets when juvenile, but we found in the field juveniles with linear leaflets only (Gutiérrez-Ortega et al. 2021: 2009; Fig. 23D). Furthermore, we did not find populations near Yajalón or Tila that correspond to *C.sancheziae*. The paratypes cited from Yajalón (*Méndez Ton 5498*, *5722* both MEXU) have some vegetative characters that do not correspond with the species description; however, these could be part of the overall species variation of *C.sancheziae*. Considering that sympatry is possible in this genus and that there is broad variation in *C.zoquorum*, research at population level with reproductive structures and molecular variation could clarify what species is represented by these plants collected near Yajalón. In addition, we have extended the range for *C.sancheziae* to the south of Chiapas (i.e., populations from Altamirano municipality).

##### Specimens examined.

Mexico. **Chiapas: Mun. Altamirano**, 1,215 m, 16 Jul 2021, *F. Nicolalde-Morejón et al. 3691–3697* (CIB); 1,215 m, 16 Jul 2021, *L. Martínez-Domínguez 2318–2325* (CIB); 1,210 m, 28 Nov 1996, *M.A. Pérez-Farrera 1468* (CHIP); 1,200 m, 18 May 1993, *M. González-Espinosa et al.*, *1973* (CH). **Mun. Tenejapa**, 1,000 m, 5 Jun 1972, *D.E. Breedlove 25506* (MEXU); 1,536 m, 15 Jul 2021, *F. Nicolalde-Morejón et al. 3683–3690* (CIB); 1,536 m, 15 Jul 2021, *L. Martínez-Domínguez et al. 2310–2317* (CIB). **Mun. Yajalón**, 1,434 m, 26 Aug 2015, *F. Hernández-Najarro 4382* (CHIP).

#### ﻿29. Ceratozamia santillanii

Taxon classificationPlantaeCycadalesZamiaceae

Pérez-Farr. & Vovides, Syst. Biodivers. 7 (4): 435. 2009

40FB50ED-8EC0-5A09-B4FC-8A8E6D0AC781

Figs 1B , 27E

##### Type.

Mexico. Chiapas: Mun. Berriozábal, road Berriozábal-El Cairo, 15 Oct 2004, *M.A. Pérez Farrera 3030* ♀ (holotype: HEM! [HEM020981]; isotypes: XAL! [XAL0005415], MEXU [n.v.]).

##### Description.

***Stem*** 10–50 cm long, 11–13 cm in diameter, semi-hypogeous, erect and decumbent. ***Cataphylls*** 2–4.5 × 1.7–4 cm wide at the base, persistent, triangular, reddish brown, densely brown tomentose at emergence, with apex partiality glabrous at maturity, apex acuminate. ***Leaves*** 2–3, 50.5–79.3 cm long, descending, light green and glaucous at emergence, with whitish gray trichomes, green and glabrous at maturity. ***Petiole*** 23.5–45 cm long, terete, linear, yellowish green green in mature leaves; with 4–15 thin prickles, 0.05–0.15 cm long or unarmed. ***Rachis*** 23–40 cm long, terete, linear, yellowish green green at emergence, yellowish green in mature leaves, unarmed to armed with prickles. ***Leaflets*** 6–12 pairs, opposite to subopposite, insertion in one plane, oblong, longitudinally curved abaxially to planar, not basally falcate to basally falcate, coriaceous, flat, green with adaxial and abaxial sides glaucous, distal end with entire margins, acuminate and asymmetrical at the apex, attenuate at base, with conspicuous and green-light veins; median leaflets 17.4–30.6 × 4.2–6.5 cm, 3.5–7.1cm between leaflets; articulations 0.7–1.1 cm wide, yellow. ***Pollen strobili*** 15–20 cm long, 1.5–3.0 cm in diameter, solitary, cylindrical, erect, green with blackish trichomes at emergence, yellow-cream with blackish at maturity; peduncle 2–4 cm long, 1.0–1.3 cm in diameter, scarce pubescent, reddish brown to brown; microsporophylls 1.2–2 × 0.5–1 cm, discoid with a non-recurved distal face and a lobate fertile portion, infertile portion 0.34–0.37 cm long and linear with straight horns 0.20–0.25 cm long, 0.50–0.65 cm and an acute angle between the horns. ***Ovulate strobili*** 12–20 cm long, 7–9 cm in diameter, solitary, cylindrical, erect, green with blackish trichomes at emergence, green with scarce blackish trichomes at maturity, acute apex; peduncle 2–3 cm long, 0.9–1.3 cm in diameter, erect, tomentose, light brown; megasporophylls 21–56, 7–8 orthostichies with 3–7 sporophylls per orthostichy, 1.3–2.2 × 1.5–2.2 cm, with a prominent distal face, horns straight and 0.60–0.80 cm long, 1.65–1.90 cm between horns with an acute angle between the horns. ***Seeds*** 2.2–2.4 cm long, 1.8–2.0 cm in diameter, ovate, sarcotesta whitish pink when immature, light brown at maturity.

##### Distribution and habitat.

**Figure 24.**
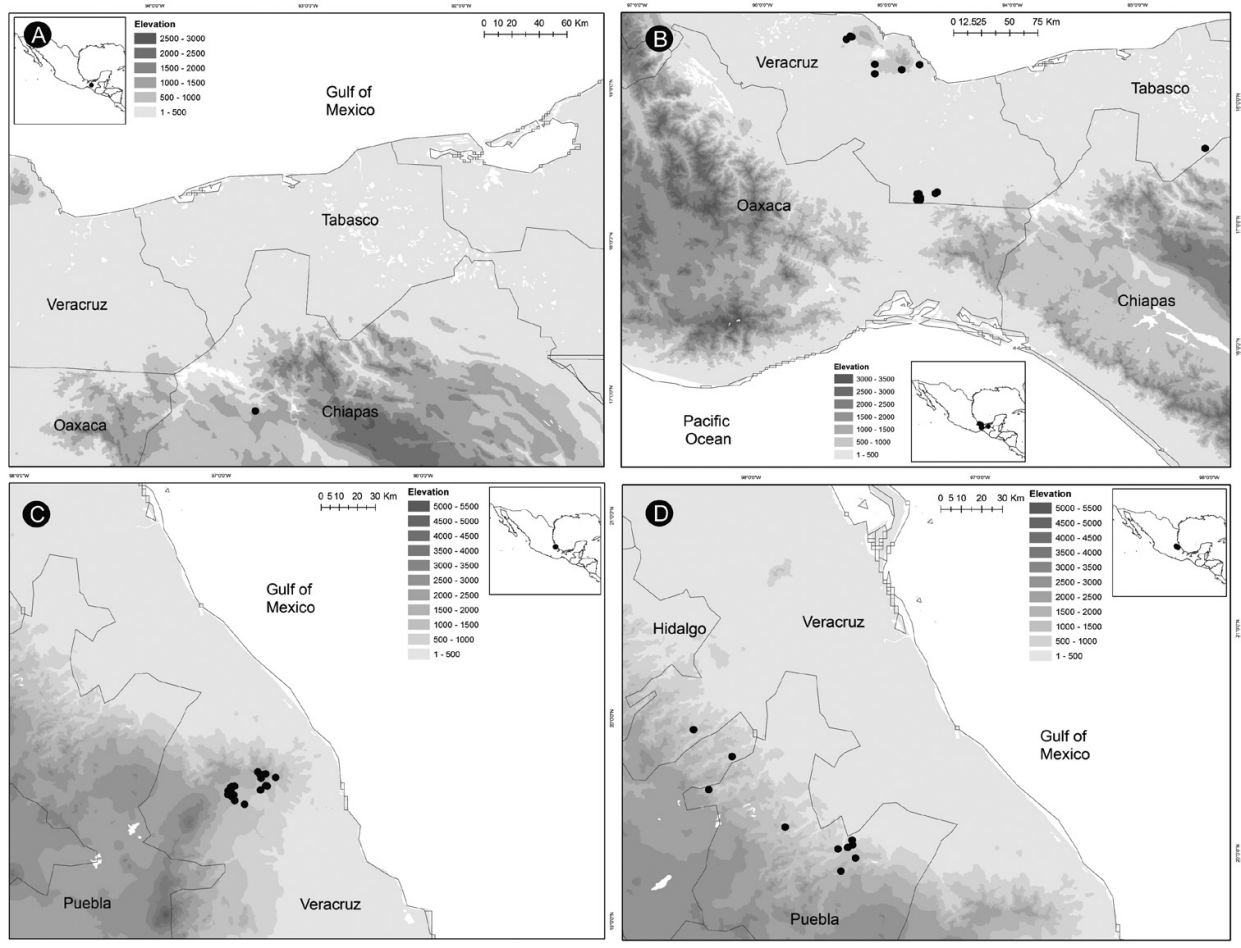
Distribution of *Ceratozamia* species. **A***C.santillanii***B***C.subroseophylla***C***C.tenuis***D***C.totonacorum*.

*Ceratozamiasantillanii* is endemic to the northern highlands of Chiapas State, Mexico and only known from the type locality in the municipality of Berriozábal (Fig. 24A), where it was collected in evergreen tropical rain forest on karstic rocks at 800–900 m.

##### Etymology.

The specific epithet was assigned in honor of Professor Trinidad Alemán Santillán in recognition of his academic accomplishments in the training of young biologists in botany and ecology (Pérez-Farrera et al. 2009).

##### Common names.

None recorded.

##### Uses.

None recorded.

##### Preliminary conservation status.

According to IUCN criteria *Ceratozamiasantillanii* should be listed as “Critically Endangered” (CR).

##### Discussion.

*Ceratozamiasantillanii* belongs to a cryptic taxonomic group with *C.zoquorum* and *C.becerrae*, which is characterized by oblong and coriaceous leaflets with an acuminate and asymmetric apex. *C.santillanii* is easily distinguished from the other taxa in this complex with internal transcribed spacer region of nuclear ribosomal (nrITS) (Martínez-Domínguez et al. 2017c), and peduncle of ovulate strobilus 3 cm long or shorter.

##### Specimens examined.

Mexico. **Chiapas**: Known only from the type locality. 15 Oct 2004, *M.A. Pérez-Farrera 2944* (HEM).

#### ﻿30. Ceratozamia subroseophylla

Taxon classificationPlantaeCycadalesZamiaceae

Mart.-Domínguez & Nic.-Mor., Phytotaxa 268(1): 35. 2016

C00E8546-A355-5D3C-A569-ECB311880B43

Figs 12D , 25 , 26

Ceratozamia dominguezii Pérez-Farr. & Gut.Ortega, Taxonomy 1: 353. 2021. Type: Mexico. Veracruz: Mun. Uxpanapa, 130 m, 29 May 2021, *M.A. Pérez-Farrera 4013* (holotype: HEM [n.v]; isotypes: MEXU [n.v], XAL [n.v]).

##### Type.

Mexico. Veracruz: Mun. Santiago Tuxtla, hill in front Sinapán, 425 m, 15 Jul 2014, *L. Martínez-Domínguez 158* ♀ (holotype: CIB! [acc. # 16893UV]; isotypes: MEXU! [MEXU01446538–MEXU01446542], NY!).

##### Description.

***Stem*** 30–250 (500) cm long, 18–45 cm in diameter, epigeous, erect and decumbent. ***Cataphylls*** 5–7 × 2–2.5 cm wide at the base, persistent, triangular, reddish brown, densely brown tomentose at emergence, glabrous at maturity, apex acuminate. ***Leaves*** 10–83, 72–370 cm long, ascending, yellowish brown at emergence, with whitish gray trichomes, glabrous at maturity. ***Petiole*** 50–150 cm long, terete, linear, greenish brown or dark brown at emergence, green in mature leaves; with 25–55 robust prickles, 0.40–0.85 cm long. ***Rachis*** 80–255 cm long, terete, linear, greenish brown or dark brown at emergence, green in mature leaves, with prickles. ***Leaflets*** 23–48 pairs, opposite to subopposite, insertion in one plane, lanceolate, abaxially curved longitudinally along distal half, not basally falcate, papyraceous, flat, dark green with adaxial side glabrous and abaxial side glaucous, acuminate and symmetrical at the apex, attenuate at base, with conspicuous and light-green veins; median leaflets 18–45 × 2.5–4 cm, 1.7–6.5 cm between leaflets; articulations 0.7–1.9 cm wide, brown in young leaves and green in mature leaves. ***Pollen strobili*** 15–30 cm long, 3.1–5.4 cm in diameter, solitary, cylindrical, erect, green with reddish trichomes at emergence, greenish yellow at maturity with dark brown trichomes; peduncle 9.5–19 cm long, 1.5–2.7 cm in diameter, tomentose, reddish brown; microsporophylls 1.47–2.8 × 1.01–1.4 cm, obconic with a non-recurved distal face and lobate fertile portion, infertile portion 0.49–0.65 cm long and rounded with straight horns 0.20–0.30 cm long, 0.48–0.53 cm and an acute angle between the horns. ***Ovulate strobili*** 15.5–40 cm long, 7–11.6 cm in diameter, solitary, cylindrical, erect, yellow with reddish to purple trichomes at emergence, green and megasporophylls with base pale pink and dark brown to reddish brown trichomes at maturity, mucronate apex; peduncle 9.8–17.5 cm long, 1.8–2.3 cm in diameter, erect, tomentose, light brown; megasporophylls 110–210, 9–12 orthostichies with 11–20 sporophylls orthostichy, 1.8–2.5 × 2.5–4.47 cm, with a prominent distal face, horns straight 0.34–1.0 cm long and 0.70–1.40 cm between horns with a right angle between the horns. ***Seeds*** 2.9–3.8 cm long, 0.80–1.75 cm in diameter ovate, sarcotesta whitish pink when immature, light brown at maturity.

##### Distribution and habitat.

*Ceratozamiasubroseophylla* occurs from southeastern Veracruz State to Tabasco State, Mexico including the montane zone of the region Santiago Tuxtla and Uxpanapa (Fig. 24B). It occurs on soils of volcanic origin and karstic rocks in evergreen tropical rain forest between 111 and 1,050 m.

##### Etymology.

The specific epithet efers to the rosaceous (i.e., pale pink) color at the base of megasporophylls.

##### Common names.

Mexico. Veracruz: Hymniom pekmuk (Popoluca ethnic group) (Leonti 542).

##### Uses.

The stem is boiled to make medicinal tea to treat kidney stones (Leonti 542).

##### Preliminary conservation status.

*Ceratozamiasubroseophylla* has several populations throughout its distribution range, but anthropogenic land-use changes affect the populations of Veracruz and Tabasco. According to the IUCN Red List criteria, we recommend “Endangered” (EN) for this species.

##### Discussion.

**Figure 25.**
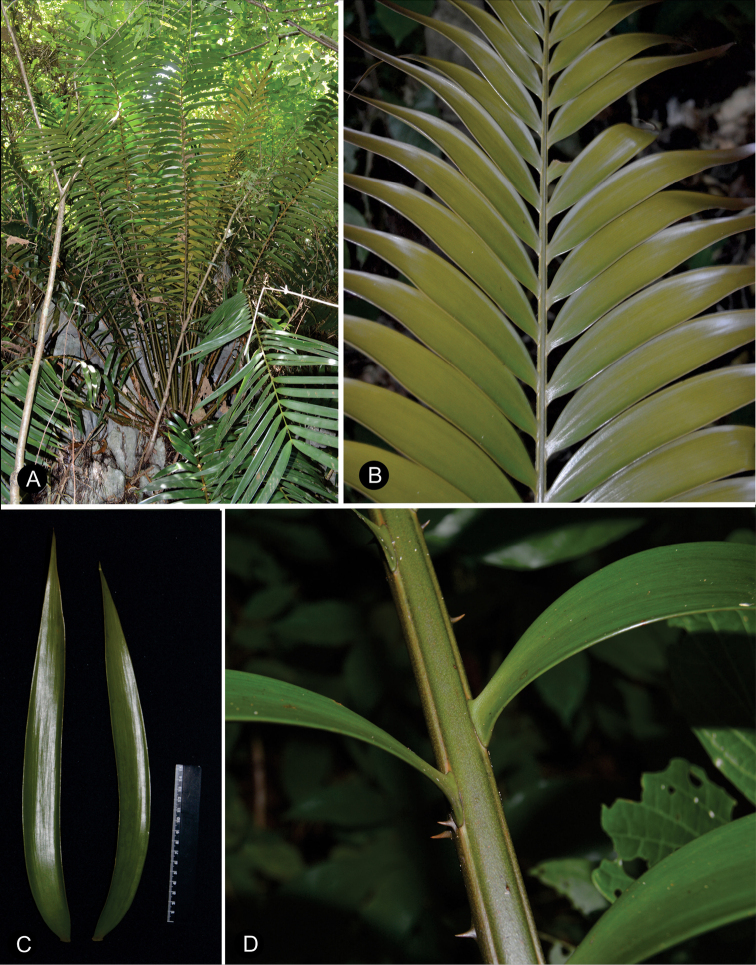
Vegetative characters of *Ceratozamiasubroseophylla***A** adult plant (from Uxpanapa, Veracruz) **B** young leaf (from Santiago Tuxtla, Veracruz) **C** new leaves of adult plant (from Santiago Tuxtla, Veracruz) **D** prickles on rachis (from Uxpanapa, Veracruz).

**Figure 26.**
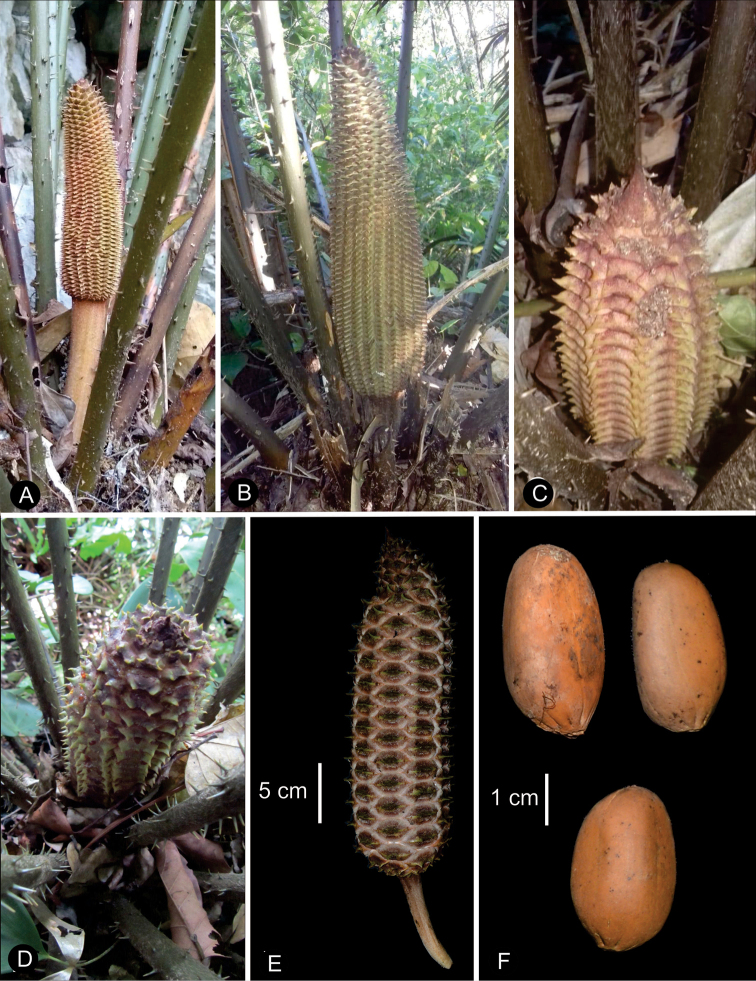
Reproductive characters of *Ceratozamiasubroseophylla***A** immature pollen strobilus (from Santiago Tuxtla, Veracruz) **B** pollen strobilus at maturity (from Santiago Tuxtla, Veracruz) **C** immature ovulate strobilus (from Uxpanapa, Veracruz) **D** immature ovulate strobilus (from Santiago Tuxtla, Veracruz) **E** ovulate strobilus at maturity (Uxpanapa, Veracruz) **F** seeds.

*Ceratozamiasubroseophylla* is distinguished from *C.leptoceras* and *C.oliversacksii* by its green-brownish petiole and rachis with abundant and robust prickles, and its lanceolate and papyraceous leaflets (Fig. 25). In addition, *C.subroseophylla* has affinity with *C.robusta*, but it is easily identified by the ovulate strobilus which has mucronate apex, rosaceous base of megasporophylls rosaceous with dark brown to reddish brown trichomes at maturity (Fig. 26).

Populations from Uxpanapa (Veracruz) were recently described as *Ceratozamiadominguezii*, however, these fall within the range of variation of *C.subroseophylla* as circumscribed here. Our circumscription is based on comparative morphology, both vegetative and reproductive structures, and phenology (Figs 25, 26).

##### Specimens examined.

Mexico. **Tabasco: Mun. Macuspana**, 185 m, 13 Jul 2021, *F. Nicolalde-Morejón et al. 3674–3680* (CIB); 185 m, 13 Jul 2021, *L. Martínez-Domínguez et al. 2295–2300* (CIB), 190 m, 13 Jul 2021, *L. Martínez-Domínguez et al. 2301* (CIB, MEXU). **Veracruz: Mun. Catemaco**, 1953, *H. Bravo 26* (MEXU); 18 May 1995, *M.A. García B. et al. 813* (XAL). **Mun. Hidalgotitlán**, 150 m, 16 Jan 1975, *Brigada Vázquez 1760* (MO, XAL); 140 m, 16 Dec 1974, *J. Rees 1655* (XAL); 16 Apr 1975, *M. Vázquez-Torres 1760* (MEXU). **Mun. Hueyapan de Ocampo**, 400 m, 14 May 2000, *Leonti 542* (MEXU). **Mun. Jesús Carranza**, 100 m, 19 Feb 2009, *D. Jimeno-Sevilla 1045* (MEXU, MO, XAL); 131 m, 27 Sep 2020, *L. Martínez-Domínguez et al. 2040* (CIB); *M. Vázquez-Torres 2430* (CHAPA, ENCB, MEXU, NY, SLPM, XAL). **Mun. Mecayapan**, 950 m, 26 Jan 1992, *M. Vázquez-Torres et al. 4122* (CIB). **Mun. Santiago Tuxtla**, 500 m, 5 Jul 1983, *D.W. Stevenson et al. 539 A–L* (NY), *540 F–H* (NY); 420 m, 28 Mar 2014, *F. Nicolalde-Morejón & L. Martínez-Domínguez 1985–1987* (CIB); 250–500 m, 30 Sep 1983, *J.F. Ortega O. et al. 358* (XAL); 420 m, 28 Mar 2014, *L. Martínez-Domínguez & F. Nicolalde-Morejón 78, 79* (NY), *80, 81* (XAL), *82–87* (CIB); 420 m, 7 Jun 2014, *L. Martínez-Domínguez & F. Nicolalde-Morejón 129* (CIB); 425 m, 15 Jul 2014, *L. Martínez-Domínguez 136–157, 159* (CIB); 500 m, 12 May 1965, *M. Sousa 2420* (MEXU); 23 Aug 1962, *R.F. Andrle 64* (US); 497 m, 17 Jan 2001, *T.W. Walters TW-2001-17* (MEXU, XAL). **Mun. Soteapan**, 21 Jun 1963, *G.N. Ross 57* (US); 1,050 m, 17 Mar 1968, *M. Sousa 3645* (MEXU); 500 m, 18 May 1986, *M. Vázquez-Torres et al. 3579* (CIB). **Mun. Tatahuicapan de Juárez**, 849 m, *C.I. Carvajal-Hernández & M. Juárez F. 796* (CIB). **Mun. Uxpanapa**, 131 m, 27 Sep 2020, *L. Martínez-Domínguez et al. 2040* (CIB, MEXU); 111 m, 27 Sep 2020, *L. Martínez-Domínguez et al. 2041* (CIB), *2042* (CIB, MEXU), *2043–2048* (CIB); 111 m, 11 Jun 2021, *L. Martínez-Domínguez et al. 2273* (CIB).

#### ﻿31. Ceratozamia tenuis

Taxon classificationPlantaeCycadalesZamiaceae

(Dyer) D.W.Stev. & Vovides, Bot. Sci. 94 (2): 425. 2016

C0EA4599-4A56-5E52-9903-1B2A4F57ABDF

Fig. 27F

Ceratozamia tenuis Type. Cultivated in England at the Royal Botanic Gardens Kew “Hort. Kew Palm House”, 1881, *Anon. s.n.* (lectotype, designated by Vovides et al. 2016, pg. 425: K! [K001092673, K001092674]). Mexico. Veracruz: Mun. Jilotepec, 19 Jan 1976, *A.P. Vovides 18* ♂ (epitype, designated by Martínez-Domínguez et al. 2018a, 117: XAL! [acc. # 16980]; isoepitype: NY!).
Ceratozamia mexicana Brongn. var. vulgaris J.Schust., Pflanzenr. (Engler) Heft 99, 4 fam 1: 131. 1932. Mexico. Veracruz: Mun. Xalapa, Chiltoyac, 1,270 m, 18 Oct 2016, *L. Martínez-Domínguez et al. 984* (neotype, designated by Martínez-Domínguez et al. 2018a, pg. 117: CIB! [acc. # 17988UV]).
Ceratozamia mexicana f. tenuis (Dyer) J.Schust., Pflanzenr. (Engler) Heft 99, 4 fam 1: 132. 1932, as “Ceratozamiamexicanavar.longifoliaf.tenuis”. Type: Based on CeratozamiamexicanaBrongn.var.tenuis Dyer

##### Type.

Based on CeratozamiamexicanaBrongn.var.tenuis Dyer, Biol. Cent.-Amer., Bot. 3: 193. 1884.

##### Description.

***Stem*** 20–100 cm long, 30–45 cm in diameter, epigeous, erect and decumbent. ***Cataphylls*** 2–6 × 2–5.5 cm wide at the base, persistent, triangular, reddish brown, densely brown tomentose at emergence, partially tomentose at maturity, apex acuminate. ***Leaves*** 6–56, 85–225 cm long, ascending, dark green at emergence with brown trichomes, glabrous at maturity. ***Petiole*** 30–93 cm long, terete, linear, green in mature leaves; with 10–55 thin prickles, 0.20–0.49 cm long. ***Rachis*** 56–154 cm long, terete, linear, green in mature leaves, with prickles. ***Leaflets*** 30–56 pairs, opposite to subopposite, insertion in one plane, linear, longitudinally curved abaxially to planar, basally falcate, papyraceous, involute, green with adaxial and abaxial sides glabrous, distal end with entire margins, acuminate and symmetrical at apex, attenuate at base, with conspicuous and light-green veins; median leaflets 23–50.5 × 1–2.1 cm, 0.3–2.5 cm between leaflets; articulations 0.4–1.4 cm wide, green. ***Pollen strobili*** 25–50 cm long, 4–8 cm in diameter, solitary, cylindrical, erect, greenish yellow at emergence, greenish yellow with blackish trichomes at maturity; peduncle 3.7–22 cm long, 1.2–2.5 cm in diameter, tomentose, reddish brown to light-brown; microsporophylls 1.7–2.7 × 1.2–1.9 cm, obconic with a non-recurved distal face and a lobate fertile portion, infertile portion 0.50–0.65 cm long and orbicular with recurved horns 0.25–0.46 cm long, 0.35–0.65 cm between the horns and a right angle between the horns. ***Ovulate strobili*** 22–35 cm long, (7.6) 10–14 cm in diameter, solitary, cylindrical, erect, dark green with blackish trichomes at emergence, dark green with blackish trichomes at maturity, acuminate apex; peduncle 8–23 cm long, 1.5–2.4 cm in diameter, erect or pendulous, tomentose, brown to reddish brown; megasporophylls (48) 80–196, 7–16 orthostichies with 6–14 sporophylls per orthostichy, 2.1–3.1 × 3.0–5.0 cm, with a prominent distal face, horns curved and 0.32–0.80 cm long, 0.80–1.60 cm between horns with a right angle between the horns. ***Seeds*** 2.5–3 cm long, 1.3–1.8 cm in diameter, ovate, sarcotesta whitish yellow to yellow when immature, light brown at maturity.

##### Distribution and habitat.

*Ceratozamiatenuis* is endemic to the central montane region in Veracruz State, Mexico (Fig. 24C), where it occurs in cloud forest at 1,200–1,920 m elevation on volcanic soils with basaltic rocks.

##### Etymology.

The specific epithet is derived from its thin leaflets.

##### Common names.

Mexico. Veracruz: Costilla de león (L. Martínez-Domínguez et al. 573); palma del monte (L. Martínez-Domínguez et al. 166).

##### Uses.

Ovulate strobili are used as an insecticide; these are cut in half and mixed with milk or sugar to kill flies (L. Martínez-Domínguez et al. 980).

##### Conservation status.

*Ceratozamiatenuis* has not been assessed for The IUCN Red List of Threatened Species. This species has populations with several adult plants (between 100 to 300); however, the total area of distribution is narrow and it is one of the areas that is highly affected by changes in land use in recent years. According to IUCN criteria this species should be considered as “Endangered” (EN) under A1acd; B1ab(iii).

**Figure 27.**
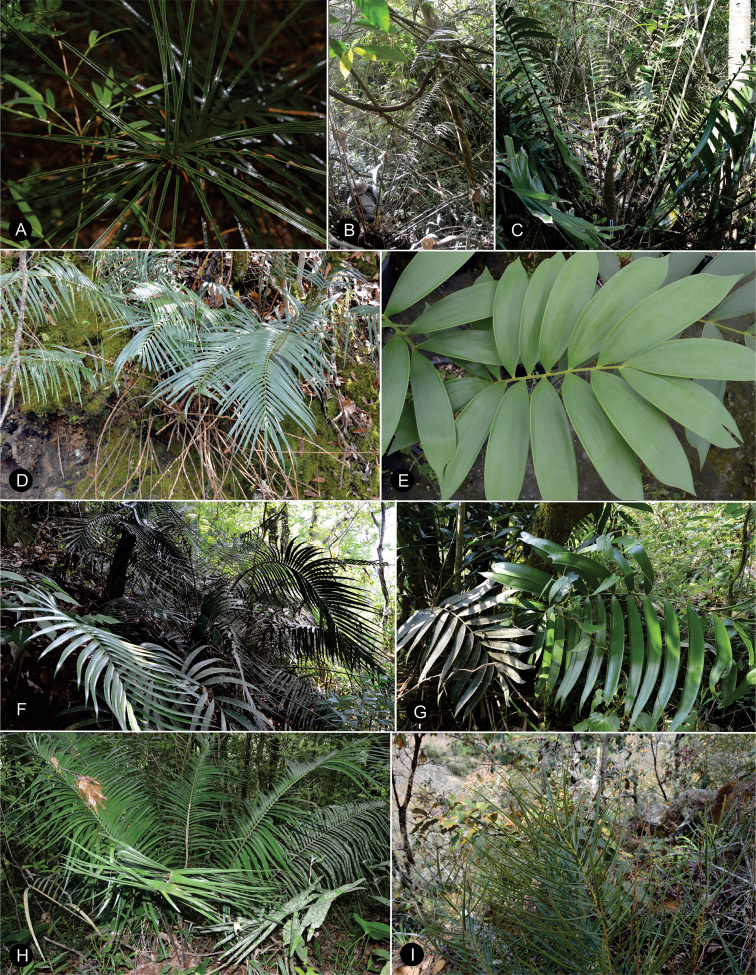
Plants of *Ceratozamia* in habitat. **A***C.norstogii***B***C.oliversacksii***C***C.robusta***D***C.sabatoi***E***C.santillanii***F***C.tenuis***G***C.totonacorum***H***C.vovidesii***I***C.zaragozae*.

##### Discussion.

*Ceratozamiatenuis* is characterized by a petiole with thin prickles, and linear leaflets that are papyraceous and involute with a symmetric apex. The ovulate strobilus is dark green with blackish trichomes at maturity, a prominent distal face, and a right angle between the horns.

##### Specimens examined.

Mexico. **Veracruz: Mun. Banderilla**, 1,450 m, 21 Apr 2017, *L. Martínez-Domínguez et al. 1000* (CIB). **Mun. Chiconquiaco**, 1,800 m, 26 Nov 1974, *D. Jimeno-Sevilla 754* (XAL); 1,916 m, 27 Sep 2016, *F. Nicolalde-Morejón et al. 2456*–*2464* (CIB); 1,800 m, 26 Nov 1974, *J. Rees 1625* (XAL), *1626* (MEXU, XAL); 1,916 m, 27 Sep 2016, *L. Martínez-Domínguez et al. 971, 973*–*981* (CIB), *972* (CIB, MEXU); 13 Apr 1967, *R. Fernandez-Nava 385A* (MEXU). **Mun. Coacoatzintla**, 8 Mar 1985, *F. Vazquez B*. *2275* (XAL); 1,400 m, 7 Jan 1977, *G. Castillo-Campos 118* (XAL); 1,550 m, 3 Jun 2005, *L.H. Bojórquez-Galván & A.M. Zapata-Aquino 1484, 1485* (CIB); 1,435 m, 9 Jan 2015, *L. Martínez-Domínguez et al. 165*–*184* (CIB); 1, 435 m, 18 Mar 2015, *L. Martínez-Domínguez et al. 273–282* (CIB); 1,540 m, 9 Feb 2016, *L. Martínez-Domínguez et al*. *759* (CIB). **Mun. Jilotepec**, 1,300 m, 29 Feb 1980, *A.P. Vovides 470* (XAL), *471* (IBUG, MEXU, XAL); 1,300 m, 14 Apr 1982, *735* (XAL); 1,385 m, 15 Nov 1978, *E. Estrada et al. 757* (MEXU); 1,316 m, 22 Aug 2014, *F. Nicolalde-Morejón* & *L. Martínez-Domínguez 2067*–*2086* (CIB); 1,350 m, 7 Dec 1970, *F. Ventura A. 2936* (ENCB); 1,300 m, 22 Jan 1971, *F. Ventura A. 3014* (ENCB); 1,300 m, 18 Nov 1974, *J. Rees 1620* (XAL); 1,363 m, 29 May 2015, *L. Martínez-Domínguez et al. 573*–*583* (CIB); 1,250 m, 23 Aug 1973, *M.G. Zola 657* (XAL), *667* (MEXU, XAL); 1,250 m, 23 Aug 1975, *R. Ortega J. 525* (XAL); Mar 2001, *S. Avendaño 5395* (MEXU). **Mun. Tepetlán**, 1,420 m, 12 Jan 2013, *F. Nicolalde-Morejón et al. 1691*–*1710* (CIB); 1,418 m, 21 Jun 2014, *F. Nicolalde-Morejón & L. Martínez-Domínguez 2001*–*2004* (CIB); 1,418 m, 22 Aug 2014, *F. Nicolalde-Morejón & L. Martínez-Domínguez 2047*–*2066* (CIB); 1,662 m, 10 Apr 2015, *F. Nicolalde-Morejón & L. Martínez-Domínguez 2217*–*2226* (CIB); 1,421 m, 26 Jul 2014, *L. Martínez-Domínguez et al. 160* (CIB); 22 Mar 2015, *L. Martínez-Domínguez & F. Nicolalde-Morejón 283*–*293* (CIB); 1,662 m, 10 Apr 2015, *L. Martínez-Domínguez & F. Nicolalde-Morejón 545–555* (CIB); 2 Jul 2010, *M. Vázquez-Torres et al. 9215* (CIB). **Mun. Tlacolulan**, 1,540 m, 16 Jun 2017, *F. Nicolalde-Morejón et al. 2516* (CIB); 1,540 m, 16 Jun 2017, *L. Martínez-Domínguez et al. 1041, 1042* (CIB). **Mun. Xalapa**, 1,270 m, 18 Oct 2016, *F. Nicolalde-Morejón et al. 2466–2468* (CIB); 1,270 m, 18 Oct 2016, *L. Martínez-Domínguez et al. 985, 987* (CIB), 986 (CIB, MEXU).

#### ﻿32. Ceratozamia totonacorum

Taxon classificationPlantaeCycadalesZamiaceae

Mart.-Domínguez & Nic.-Mor., Brittonia 69 (4): 518. 2017

7DCCF1E7-5A39-53AF-A5C2-A3ECF6D7FABA

Figs 27G , 28B

##### Type.

Mexico. Puebla: Mun. Jonotla, 600 m, 9 Jun 2015, *L. Martínez-Domínguez* & *F. Nicolalde-Morejón 618* ♀ (holotype: CIB! [acc. # 16735UV]).

##### Description.

***Stem*** 10–45 cm long, 10–25 cm in diameter, epigeous, erect and decumbent. ***Cataphylls*** 2–5 × 1.2–2.5 cm wide at the base, persistent, triangular, reddish brown, densely brown tomentose at emergence, glabrous at maturity, apex acuminate. ***Leaves*** 10–63, 100–265 cm long, descending, brown at emergence, with brown trichomes, glabrous at maturity. ***Petiole*** 30–80 cm long, terete, straight, green in mature leaves; with 10–40 thin prickles, 0.05–0.25 cm long. ***Rachis*** 85–185 cm long, terete, straight, green in adult leaves, with prickles. ***Leaflets*** 11–33 pairs, opposite to subopposite, insertion in one plane, oblong, longitudinally planar, not basally falcate to occasionally falcate, papyraceous, flat, green with adaxial side glabrous and abaxial side glaucous, distal end with entire margins, acuminate and symmetrical at the apex, attenuate at base, with conspicuous and light-green veins; median leaflets 17–40 × 2.7–4.2 cm, 2–5.6 cm between leaflets; articulations 0.5–1.3 cm wide, green. ***Pollen strobili*** 28–31 cm long, 5.0–6.0 cm in diameter, generally solitary (up to 2), cylindrical, erect, greenish yellow at emergence, yellow with brown trichomes at maturity; peduncle 9–12 cm long, 1.5–2 cm in diameter, tomentose, light brown; microsporophylls 1.5–2.7 × 0.9–2.0 cm, obconic with a non-recurved distal face and a lobate fertile portion, infertile portion 0.39–0.57 cm long and rounded with straight horns 0.19–0.25 cm long, 0.52–0.69 cm and a right angle between the horns. ***Ovulate strobili*** 20.5–28.7 cm long, 8.4–9.3 cm in diameter, solitary, cylindrical, erect, light green and glaucous, with orange to light brown trichomes at emergence, green with yellowish brown trichomes at maturity, acuminate apex; peduncle 10–11.2 cm long, 1.5–2.4 cm in diameter, erect or pendulous, tomentose, light brown; megasporophylls 64–120, 8–10 orthostichies with 8–13 sporophylls per orthostichy, 1.6–2.3 × 2.6–3.6cm, with a prominent distal face, horns straight and 0.55–0.80 cm long, 1.45–1.80 cm between horns with a right angle between the horns. ***Seeds*** 2.5–3.5 (4) cm long, 0.88–1.6 cm in diameter, ovate, sarcotesta whitish red when immature, cream to light brown at maturity.

##### Distribution and habitat.

*Ceratozamiatotonacorum* occurs in the Sierra Norte de Puebla and the mountain region in Hidalgo and Veracruz States, Mexico (Fig. 24D). It occurs in cloud forest and the transition zone between evergreen tropical forest and cloud forest on rocky outcrops in exposed walls up to 80 m tall at 600–1,800 m.

##### Etymology.

The specific epithet is in reference to the Totonaco ethnic group of Santiago Ecatlán in Sierra Norte of Puebla, Mexico.

##### Common names.

Mexico. Puebla: Kun (Totonaco ethnic group) (Martínez-Domínguez et al. 2017b).

##### Uses.

**Figure 28.**
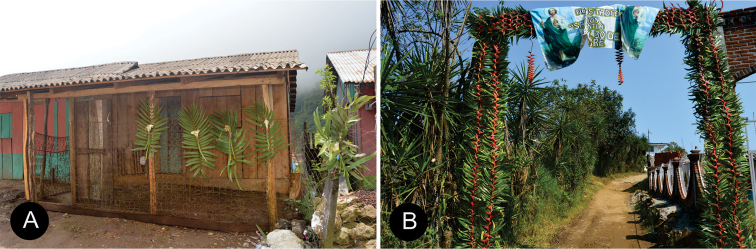
Leaves of *Ceratozamia* are used for religious ceremonies **A***C.morettii* used during local celebrations in La Estrella (Chiconquiaco municipality, Veracruz, Mexico) **B***C.totonacorum* used during “San Judas Tadeo” festivities in La Unión Atioyan (Nauzontla municipality, Puebla, Mexico).

In Sierra Norte of Puebla, the residents use the leaves of this species in local rituals to make “arcos” and altars (Fig. 28B) (Martínez-Domínguez et al. 2017b).

##### Preliminary conservation status.

Based on total populations and the potential distribution and reduction of vegetation in the area for *Ceratozamiatotonacorum*, we recommend that it should be listed as “Vulnerable” in The IUCN Red List of Threatened Species.

##### Discussion.

*Ceratozamiatotonacorum* is distinguished from *C.delucana* by its brown leaves at emergence, but this color disappears in the mature leaves which become green; besides, the leaves are descending. The ovulate strobilus is yellowish green with brown trichomes.

##### Specimens examined.

Mexico. **Hidalgo: Mun. Huehuetla**, 1,150 m, 2 Jun 1976, *A.P. Vovides 23* (XAL). **Puebla: Mun. Atlequizayan**, 1 Apr 2014, 867 m, *L. Caamaño Onofre & A.B. Cerón Carpio 4995* (XAL). **Mun. Cuetzalan del Progreso**, 1,470 m, 1 Nov 2017, *L. Martínez-Domínguez et al. 1180–1189* (CIB). **Mun. Jonotla**, 760 m, 13 Feb 2014, *F. Nicolalde-Morejón et al. 1948* (CIB); 600 m, 13 Feb 2014, *F. Nicolalde-Morejón et al. 1949–1955* (CIB), *1956* (MEXU), *1957* (NY), *1958*–*1965* (CIB); 1,006 m, 14 Feb 2014, *F. Nicolalde-Morejón et al. 1966, 1967* (CIB); 600 m, 9 Jun 2015, *L. Martínez-Domínguez & F. Nicolalde-Morejón 619* (CIB). **Mun. Pahuatlán**, 1,800 m, 12 Jan 1987, *G. Toriz et al. 226* (MEXU). **Mun. Tlapacoya**, 1,010 m, 14 Feb 1985, *E. Meza P. 14* (XAL). **Mun. Zacapoaxtla**, 1, 365 m, 30 May 2014, *L. Camaño-Onofre 5329* (XAL). **Veracruz: Mun. Tlachichilco**, 1,300 m, 12 Sep 2001, *A. Rincón G. et al. 2584* (XAL), *2585* (MEXU, XAL).

#### ﻿33. Ceratozamia vovidesii

Taxon classificationPlantaeCycadalesZamiaceae

Pérez-Farr. & Iglesias, Bot. J. Linn. Soc. 153: 394. 2007

AE47E893-8601-5B42-9736-BE27E41194B1

Fig. 27H

##### Type.

Mexico. Chiapas: Mun. La Concordia, Between Finca Santa Cruz and El Puente, Rancho Las Cabañas, 1,156 m, 19 Jul 2001, *M.A. Pérez-Farrera 2620a* ♀ (holotype: HEM!; isotypes: CHIP [n.v.], MEXU [n.v.], MO [n.v.], XAL [n.v.]).

##### Description.

***Stem*** 50–80 cm long, 20–30 cm in diameter, epigeous, erect and decumbent. ***Cataphylls*** 5–7.5 × 2.7–5 cm wide at the base, persistent, triangular, reddish brown, densely brown tomentose at emergence, partially tomentose at maturity, apex acuminate. ***Leaves*** 3–18, 97–238 cm long, ascending, reddish brown at emergence with whitish gray trichomes, glabrous at maturity. ***Petiole*** terete, linear, 40–164 cm long, green in adult leaves, with 15–55 thin prickles, 0.14–0.38 cm long. ***Rachis*** terete, linear, 54–153 cm long, green in mature leaves, with prickles. ***Leaflets*** 30–85, opposite to subopposite, insertion in one plane, lanceolate, mostly longitudinally planar, generally basally falcate, papyraceous, flat, green with adaxial side glabrous and abaxial side glaucous, distal end with entire margins, acuminate and symmetrical apex, attenuate at base, with conspicuous and light-green veins; median leaflets 20–45 × 0.7–1.4 cm, 0.3–2.4 cm between leaflets; articulations 0.2–0.8 cm wide, green. ***Pollen strobili*** 15–45 cm long, 3.5–5 cm in diameter, solitary, cylindrical, erect, yellowish green with reddish brown trichomes at emergence, yellowish cream with reddish brown trichomes at maturity; peduncle 6–9.5 cm long, 1.6–1.9 cm in diameter, tomentose, reddish brown to brown; microsporophylls 1.2–1.5 × 0.8–1.04 cm, obconic with a non-recurved distal face and lobate fertile portion, infertile portion 0.37–0.50 cm long and rounded with straight horns 0.30–0.42 cm long, 0.52–0.84 cm with a right angle between the horns. ***Ovulate strobili*** 26–40 cm long, 7.1–9.6 cm in diameter, solitary, cylindrical, erect, greyish green with abundant reddish brown trichomes at emergence, green with abundant blackish trichomes at maturity, acuminate apex; peduncle 7–15 cm long, 1.7–2.2 cm in diameter, erect or pendulous, pubescent, brown; megasporophylls 60–70, 6–8 orthostichies with 7–12 sporophylls per row, 3.8–4.5 × 4–5 cm, with a prominent distal face, horns straight and 0.60–0.80 cm long, 0.99–1.40 cm between horns with an obtuse angle between the horns. ***Seeds*** 2.2–2.7 cm long, 1.3–1.7 cm in diameter, ovate, sarcotesta whitish yellow to yellow when immature, light brown at maturity.

##### Distribution and habitat.

**Figure 29.**
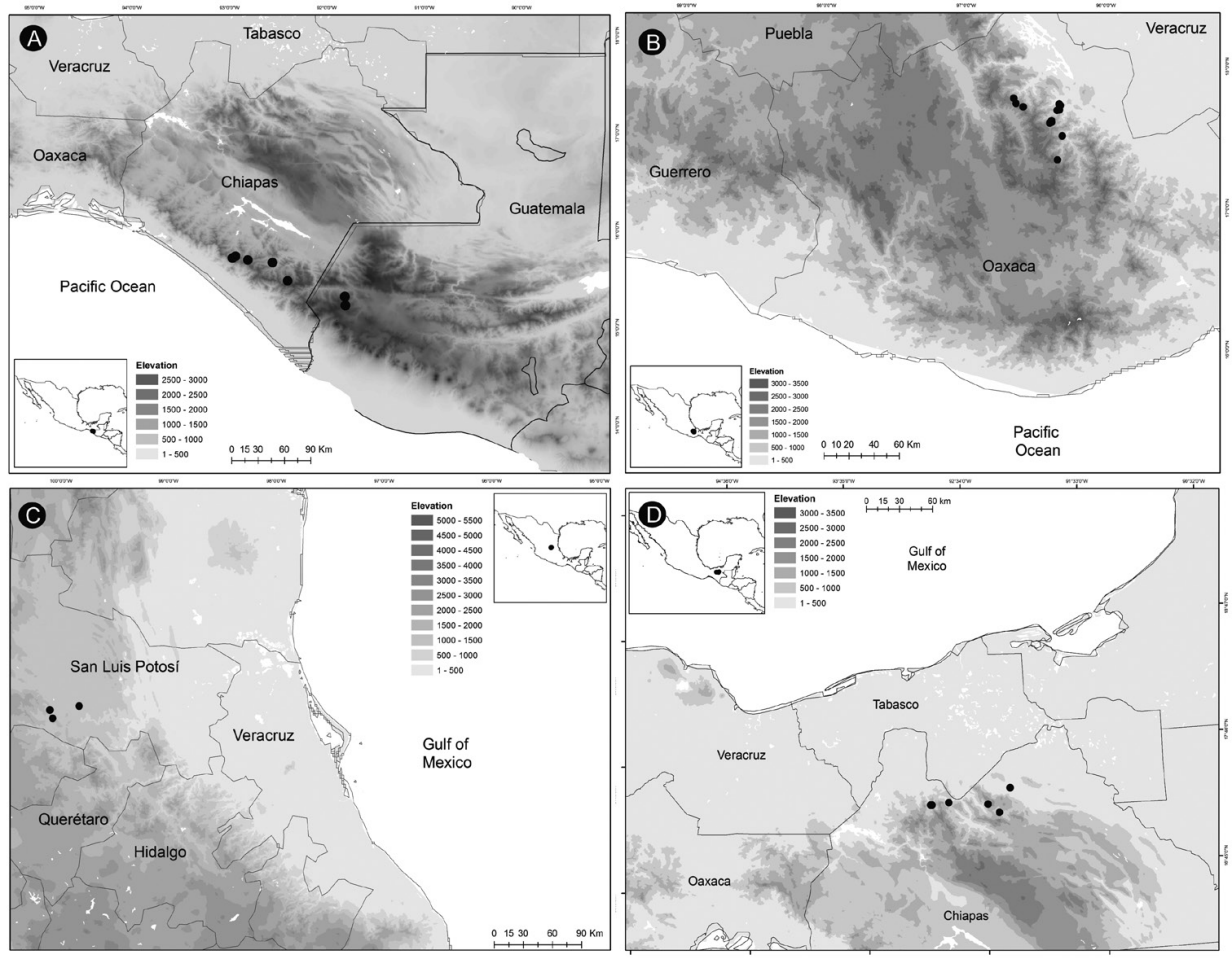
Distribution of *Ceratozamia* species **A***C.vovidesii***B***C.whitelockiana***C***C.zaragozae***D***C.zoquorum*.

*Ceratozamiavovidesii* is distributed along the Sierra Madre of Chiapas State in Mexico to Guatemala. It occurs on karstic rocks in cloud forest between 800 and 1,850 m elevation (Fig. 29A).

##### Etymology.

The specific epithet honors Andrew P. Vovides in recognition of his systematic and ecological studies and efforts in the conservation of Mexican cycads, including the creation of the Mexican National Cycad Collection at the Francisco Javier Clavijero Botanic Garden in Xalapa (Veracruz, Mexico) (Pérez-Farrera et al. 2007).

##### Common names.

None recorded.

##### Uses.

Decorative (M.A. Pérez-Farrera 2620a).

##### Conservation status.

(IUCN 2021). *Ceratozamiavovidesii* is listed as “Vulnerable” (V) under criteria D2. Here, we extended the distribution range for this species to Guatemala. Based on number of populations and data obtained during fieldwork we recommend not changing the current status.

##### Discussion.

*Ceratozamiavovidesii* shares several vegetative characteristics with *C.mirandae*. However, there are differences in their pollen strobili; *C.vovidesii* has obconic microsporophylls with rounded infertile portions, whereas those of *C.mirandae* are elliptic with a linear infertile portion.

There is some confusion concerning the holotype for *C.vovidesii*. In 2012 the gathering *Pérez-Farrera 2620^a^* also was used as the holotype in the protologue of *Zamiagrijalvensis* Perez-Farr., Vovides & Mart.-Camilo (Pérez-Farrera et al. 2012). The specimen, *Pérez-Farrera 2620^a^* is clearly a *Ceratozamia* and not a *Zamia* and corresponds to the protologue of *C.vovidesii*. Another specimen, *Pérez-Farrera 3026*, is labelled as the holotype for *Z.grijalvensis* and it clearly matches the protologue of that taxon. We are treating this miscitation of *Pérez-Farrera 2620^a^* in the protologue of *Z.grijalvensis* as a typographical error, which is now corrected.

##### Specimens examined.

Guatemala. **Huehuetenango**: 900–1,300 m, 3 Sep 1942, *J.A. Steyermark 51818* (NY; US); 1,630 m, 9 Jul 2006, *M. Véliz & V. Davila 17042, 17043, 17044* (BIGU); 30 May 1906, *O.F. Cook 51* (US); 1,629 m, 10 Jul 2006, *V. Davila & M. Véliz 1050* (BIGU); 1,622 m, 10. Jul 2006, *V. Davila & M. Véliz 1052, 1053* (BIGU). Mexico**. Chiapas: Mun. Ángel Albino Corzo**, 730 m, 23 Jan 1968, *Alush Shilom Ton 3554* (ENCB); 14 Dec 1980, 1,380 m, *D.E. Breedlove 48678* (NY); 800–1,000 m, 8 Nov 1945, *E.H. Xolocotzi & A.J. Sharp 402* (CHAPA, ENCB, MEXU); 1,000 m, 2 Jun 1987, *E.M. Martínez S. et al. 21586* (MEXU); 1,819 m, 13 Aug 2009, *H. Gómez-Domínguez 2316* (MEXU); 1,650 m, 18 May 1982, *J.I. Calzada et al. 9131* (XAL); 5 Mar 1989, *U. Bachem C. & R. Rojas 405* (CHIP). **Mun. Jaltenango de la Paz**, Jun 1995, *E. Matuda s/n* (MEXU); 1,500 m, 23 Jun 1990, *M. Heath & A. Long 1287* (CHIP); 25 Feb 1995, *Miranda 7042* (MEXU). **Mun. La Concordia**, 1,000 m, 5 Jun 1988, *E. Palacios E. 1050* (CHIP); 1,840 m, 26 Jun 2018, *F. Nicolalde-Morejón et al. 2864–2875* (CIB); 1,840 m, 26 Jun 2018, *L. Martínez-Domínguez et al. 1439–1451* (CIB); 1,156 m, 19 Sep 2001, *M.A. Pérez-Farrera 2621* (XAL); 1,600 m, *M.A. Pérez-Farrera s/n* (CHIP); 1,120 m, 17 Jun 2014, *M.G. Díaz M. 961* (CHIP); 24 Mar 2001, *R. Martínez-Camilo 54* (CHIP); 1,100 m, 11 Jun 1988, *T.G. Cabrera-Cachón 74* (CHIP); 1,700 m, 1 Jun 1989, *U. Bachem C. & R. Rojas 795* (CHIP). **Mun. Mapastepec**, 1,750 m, 13 May 1982, *J.I. Calzada et al. 8874* (IBUG, MEXU, MO, XAL). **Mun. Siltepec**, 28 Feb 2000, *O. Farrera S. 1958* (CHIP).

#### ﻿34. Ceratozamia whitelockiana

Taxon classificationPlantaeCycadalesZamiaceae

Chemnick & T.J.Greg., Phytologia 79(1): 51. 1996 (”1995”)

209342C8-5E72-5D53-B4F2-DAF76CB05429

Fig. 30

##### Type.

Mexico. Oaxaca: Vicinity of Metates, south of Valle Nacional, 628 m, 10 May 1995, *J. Chemnick & T. Gregory 5* (holotype: HNT [n.v.]; isotypes: FTG!, XALU [n.v.]).

##### Description.

***Stem*** 20–80 cm long, 18–30 cm in diameter, epigeous, decumbent. ***Cataphylls*** 2–5 × 2–5 cm wide at the base, persistent, triangular, reddish brown, densely brown tomentose at emergence, partially tomentose at maturity, apex acuminate. ***Leaves*** 3–10, 129–250 cm long, descending, light green and glaucous at emergence with whitish gray trichomes, glabrous at maturity. ***Petiole*** 60–140 cm long, terete, linear, green in mature leaves; with 3–22 thin prickles, 0.06–0.39 cm long. ***Rachis*** 70–160 cm long, terete, linear, greenish in mature leaves, unarmed. ***Leaflets*** 20–48 pairs, opposite to subopposite, insertion in one plane, lanceolate, generally longitudinally planar, not basally falcate, papyraceous, flat, green with adaxial and abaxial sides glabrous, distal end with entire margins, acuminate and symmetrical at the apex, attenuate at base, with conspicuous and green-light veins; median leaflets 25–38.5 × 2.0–3.7 cm, 1.6–3.1 cm between leaflets; articulations 0.5–1.4 cm wide, green. ***Pollen strobili*** 20–30 cm long, 3–5 cm in diameter, generally solitary (1–2), cylindrical, erect, greenish at emergence with reddish trichomes, greenish yellow with reddish brown trichomes at maturity; peduncle 15–25 cm long, 1.2–1.9 cm in diameter, glabrous or with trichomes scarce reddish brown to brown; microsporophylls 1.5–3.0 × 0.8–1.5 cm, elliptic with a non-recurved distal face and a lobate fertile portion, infertile portion 0.40–0.50 cm long and linear with straight horns 0.38–0.50 cm long, 0.50–1.0 cm and a right angle between the horns. ***Ovulate strobili*** 14–20 cm long, 7–10 cm in diameter, solitary, cylindrical, erect, yellowish green with abundant blackish trichomes at emergence, green with brown to blackish trichomes at maturity, apiculate apex; peduncle 1–4 cm long, 1.0–1.8 cm in diameter, erect, glabrous or with trichomes scarce, reddish brown; megasporophylls 24–40, 4–5 orthostichies with 5–8 sporophylls per orthostichy, 1.7–2.2 × 4.5–5.1 cm, with a truncate distal face, horns straight and 0.98–1.4 cm long, 0.95–1.3 cm between horns and an acute angle between the horns. ***Seeds*** 2.1–2.7 cm long, 1.4–1.8 cm in diameter, ovate, sarcotesta whitish yellow to yellow when immature, light brown at maturity.

##### Distribution and habitat.

*Ceratozamiawhitelockiana* is endemic to the Sierra Norte of Oaxaca (Mexico), between 500 to 1,800 m in La Chinantla area (Fig. 29B). It occurs in the elevational gradient of evergreen tropical forest with *Quercus* sp. and cloud forest with *Oreomunneamexicana* (Standl.) J.-F.Leroy on karstic rocks.

##### Etymology.

This species was named in honor of Loran Whitelock for his contributions to cycad biology (Chemnick and Gregory 1996).

##### Common names.

None recorded.

##### Uses.

None recorded.

##### Conservation status.

(IUCN 2021). *Ceratozamiawhitelockiana* is listed as “Endangered (EN)” under criteria A2c; B1ab(i,ii,iii,v)+2ab(i,ii,iii,v); C1.

##### Discussion.

**Figure 30.**
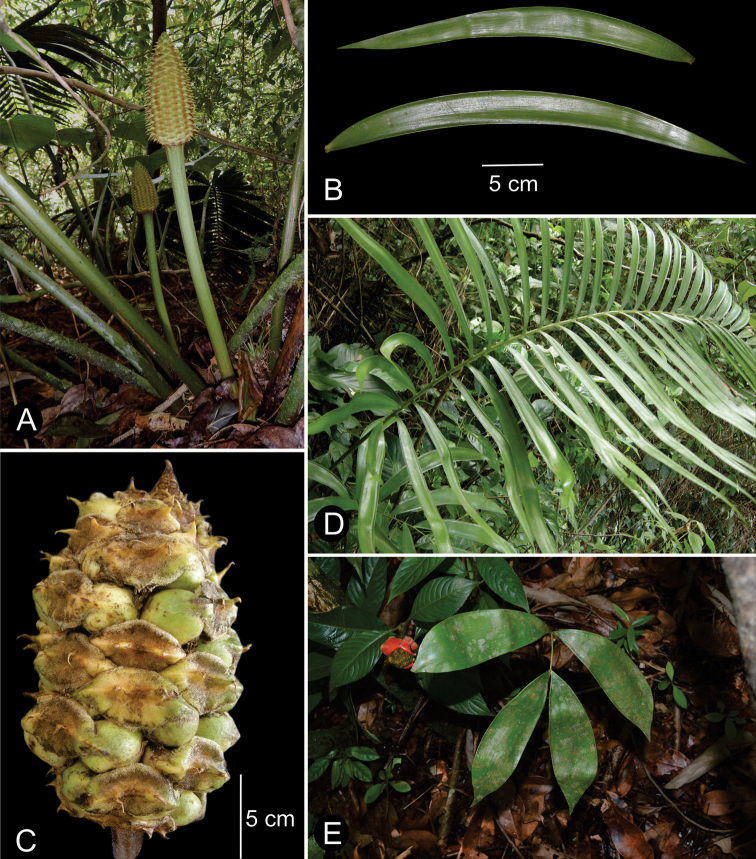
*Ceratozamiawhitelockiana***A** immature pollen strobili **B** leaflet variation **C** mature ovulate strobilus **D** leaves **E** seedling.

*Ceratozamiawhitelockiana* is similar to *C.mixeorum* in leaf morphology; the only difference is the sparse prickles in *C.whitelockiana* (between 3 to 22, in number), whereas *C.mixeorum* has more than 28 prickles. Additionally, the length of the petiole is very long in relation to the total size of the leaf in *C.whitelockiana*. In reproductive structures, the ovulate strobili in *C.whitelockiana* have a long peduncle 12–23 cm and the fertile portion has 4–5 orthostichies with 5–8 sporophylls per orthostichy and the pollen strobili have a long peduncle that is the same size as the fertile part or longer (Fig. 30 A, C). In contrast, *C.mixeorum* has ovulate strobili with a short peduncle from 1–4 cm long and pollen strobili with peduncles shorter than the fertile area.

##### Specimens examined.

Mexico. **Oaxaca**: 660 m, 29 Jun 1977, *T.B. Croat 39751* (MO). **Mun. Ixtlán de Juárez**, 1,640 m, 29 Jan 1998, *Y. Arellanes C. et al. 283* (SERO); 1,640 m, 21 Aug 1998, *Y. Arellanes C. et al. 413* (MEXU, MO, SERO). **Mun. San Felipe Usila**, 24 Oct 1994, *P. Osorio H. 312* (MEXU). **Mun. San Juan Bautista Valle Nacional**, 650 m, 24 Sep 2020, *F. Nicolalde-Morejón et al. 3346* (CIB); 650 m, 24 Sep 2020, *L. Martínez-Domínguez et al. 1968* (CIB, MEXU); 500 m, 22 Jan 2001, *S. Avendaño R. 5375* (MEXU); 518 m, 22 Jan 2001, *T.W. Walters 2001-39-D,E* (XAL). **Mun. San Juquila Vijanos**, 1,900 m, 15 Nov 1996, *X. Munn et al. 233* (XAL). **Mun. San Pedro Sochiapam**, 1,682 m, 2 Nov 2016, *M.B. Velasco-Pichardo et al. 225* (MEXU). **Mun. San Juan Tepeuxila**, 1,538 m, 9 May 2008, *J.E. Rivera-Hernández 4289* (MEXU, XAL); 1,538 m, 30 Jul 2008, *J.E. Rivera-Hernández 4380* (MEXU). **Mun. Santiago Comaltepec**, 1,750 m, 8. Jan 1995, *A. Rincón G. et al. 516* (MEXU, MO, XAL); 560 m, 24 Sep 2020, *F. Nicolalde-Morejón et al. 3348–3355* (CIB); 560 m, 24 Sep 2020, *L. Martínez-Domínguez et al. 1973–1975, 1980* (CIB, MEXU), *1976, 1978, 1979, 1981* (CIB), *1977* (CIB, MEXU, NY); 1,600 m, 10 Jun 1988, *R. López-Luna & G.J. Martin 285* (MEXU), 1,760 m, 26 Jan 1988, *R. Torres C. & E. Martínez S. 11345* (MEXU).

#### ﻿35. Ceratozamia zaragozae

Taxon classificationPlantaeCycadalesZamiaceae

Medellín-Leal, Brittonia 15: 175. 1963

50A0C7C4-D735-5C5B-98D3-23F9207941D0

Fig. 27I

##### Type.

Mexico. San Luis Potosí: Mun. Río Verde, 22 Jul 1962, *F. Medellín-Leal 1452* ♀ (holotype: SLPM! [acc. # 003530]; isotypes: ENCB! [ENCB003716], GH! [00003279], MEXU! [MEXU00162859, MEXU0053418], MICH! [1192896], US! [00011997]).

##### Description.

***Stem*** 10–20 cm long, 10–15 cm in diameter, semi-hypogeous, erect. ***Cataphylls*** 1.8–2.5 × 1–2 cm wide at the base, persistent, triangular, reddish brown, densely brown tomentose at emergence, partially tomentose at maturity, apex acuminate. ***Leaves*** 3–27, 95–202 cm long, ascending, reddish brown at emergence with whitish gray trichomes, glabrous at maturity. ***Petiole*** 11–36 cm long, terete, twisted, green in mature leaves, unarmed. ***Rachis*** 40–77 cm long, terete, twisted, green in mature leaves, unarmed. ***Leaflets*** 25–46, opposite to subopposite, insertion in one plane, linear, generally longitudinally planar, basally falcate, membranaceous, strongly involute, green with adaxial and abaxial sides glabrous, distal end with entire margins, acute and symmetrical at the apex, attenuate at base, with conspicuous and light-green veins; median leaflets 17–31.5 × 0.4–0.7 cm, 0.8–2.3 cm between leaflets; articulations 0.2–0.3 cm wide, yellow. ***Pollen strobili*** 15–19 cm long, 2–3.5 cm in diameter, solitary, cylindrical, erect, greenish with reddish brown trichomes at emergence, reddish brown at maturity; peduncle 5–8 cm long, 1.5–1.8 cm in diameter, tomentose, reddish brown to brown; microsporophylls 0.8–1.2 × 0.3–0.6 cm, obconic with a non-recurved distal face and a lobate fertile portion, infertile portion 0.25–0.35 cm long and rounded with straight horns 0.20–0.30 cm long, 0.22–0.30 cm and an obtuse angle between the horns. ***Ovulate strobili*** 7–12 cm long, 5.5–7.3 cm in diameter, solitary, cylindrical, erect, green with scarce reddish brown trichomes at emergence, dark green at maturity, acute apex; peduncle 6–9 cm long, 0.9–1.2 cm in diameter, erect, tomentose, brown; megasporophylls 24–49, 5–7 orthostichies with 5–6 sporophylls per orthostichy, 2.0–2.6 × 2.2–3.7 cm, with a truncate distal face, horns straight and 0.33–0.45 cm long, 1.95–2.35 cm between horns with an obtuse angle between the horns. ***Seeds*** 2–2.8 cm long, 1.8–2 cm in diameter, ovate, sarcotesta light brown at maturity.

##### Distribution and habitat.

*Ceratozamiazaragozae* is endemic to Mexico in a small mountain range in San Luis Potosí (Fig. 29C), where it occurs in pine-oak forest on karstic rocks at 1,500–1,950 m.

##### Etymology.

The specific epithet refers to General Ignacio Zaragoza, who was a hero in the Battle of Puebla against the French Army in May of 1862.

##### Common names.

None recorded.

##### Uses.

None recorded.

##### Conservation status.

(IUCN 2021). *Ceratozamiazaragozae* is listed as “Critically Endangered (CR)” under criteria A2acd; B1ab(iii,iv,v)+2ab(iii,iv,v); C1. Castillo-Lara et al. (2018) evaluated the population structure and spatial distribution for this species and found that the populations have several individuals with a density variable from four to 209 plants in an area of 2500 m2, but low population growth. These authors suggest that the status for this species could be modified to “Endangered (EN)”.

##### Discussion.

*Ceratozamiazaragozae* and *C.norstogii* are the only species in the genus with twisted petiole and rachis, but the first has an unarmed petiole, whereas *C.norstogii* has abundant and robust prickles on the petiole. Additionally, leaflets are membranaceous in *C.zaragozae*, whereas in *C.norstogii* they are coriaceous.

##### Specimens examined.

Mexico. **San Luis Potosí: Mun. Río Verde**, 1,700 m, 29 Mar 1984, *A.G. Mendoza & L. Vargas 1389* (MEXU); 1,750 m, 20 Sep 1979, *A.P. Vovides 435* (XAL); 22 Jul 1962, *E. Molseed 34* (MEXU; MICH); 1,860 m, 24 Jan 1994, *F. García S. s/n* (SLPM); 1,800 m, 22 Jul 1962, *F. Medellin-Leal 1451* (SLPM; US); 28 Oct 1965, *F. Medellin-Leal s/n* (SLPM); 1,900 m, Sep 1994, *F. Medellin-Leal s/n* (SLPM); 1,956 m, 18 Mar 2016, *F. Nicolalde-Morejón et al. 2307*–*2319* (CIB); 1,750 m, 13 Apr 1968, *J. Rzedowski 25658* (ENCB; MICH); 1,956 m, 18 Mar 2016, *L. Martínez-Domínguez et al. 792, 794–796, 798–808* (CIB), *793, 797, 799* (CIB, MEXU); 2031 m, 31 Jul 2017, *P. Chávez C. et al. 98* (SLPM); 1,532 m, 13 Jan 2001, *T. Walters et al. TW-2001-07* (MEXU, XAL). **Mun. Zaragoza**, 1,869 m, 9 Nov 2012, *P. Castillo-Lara et al. 593* (SLPM); 1,847 m, 14 Jul 2016, *P. Castillo-Lara et al. 1073* (SLPM).

#### ﻿36. Ceratozamia zoquorum

Taxon classificationPlantaeCycadalesZamiaceae

Pérez-Farr., Vovides & Iglesias. Bot. J. Linn. Soc. 137 (1): 77. 2001

4639B9A8-F2DA-5E83-9A7B-50B9F41ECFCD

Figs 1D , 23 A–C

##### Type.

Mexico. Chiapas: Northern mountain range, 18 Nov 1998, *M.A. Pérez-Farrera 1732* ♂ (holotype: CHIP [n.v]).

##### Description.

***Stem*** 12–30 cm long, 8–15 cm in diameter, epigeous, semi-hypogeous, erect. ***Cataphylls*** 2.5–6.9 × 1.3–4.5 cm wide at the base, persistent, triangular, reddish brown, densely brown tomentose at emergence, glabrous at apex when mature, apex acuminate. ***Leaves*** 2–17, 46–216.5 cm long, descending, light green or brown, glaucous at emergence with whitish gray trichomes, glabrous at maturity. ***Petiole*** 23.3–111.5 cm long, terete, linear, pink at emergence, yellowish green green at mature leaves; unarmed to armed with 3–27 thin prickles 0.08–0.15 cm long. ***Rachis*** 17.6–114.5 cm long, terete, linear, pink at emergence, yellowish green at mature leaves, generally unarmed. ***Leaflets*** 6–17 pairs, opposite to subopposite, insertion in one plane, oblong to oblanceolate, longitudinally curved abaxially to planar, generally basally falcate, coriaceous, flat, green with adaxial and/or abaxial side glaucous, distal end with entire margins, acuminate (rarely acute) and asymmetrical (rarely symmetrical in apical leaflets) at the apex, attenuate at base, with conspicuous and light-green veins; median leaflets 22–37 × 3.9–5.6 (7) cm, 4.6–11.1 cm between leaflets; articulations 0.5–1.5 cm wide, green and yellowish. ***Pollen strobili*** solitary (up to 2), 10.8–25 cm long, 2.8–4.3 cm in diameter, cylindrical, erect, green with blackish trichomes at emergence, yellow-cream with blackish trichomes at maturity; peduncle 5–13 cm long, 1.5–1.8 cm in diameter, tomentose, light brown; microsporophylls 0.9–1.4 × 0.7–0.9 cm, discoid with a non-recurved distal face and a deeply lobate fertile portion, infertile portion 0.35–0.40 cm long and linear with straight horns 0.30–0.45 cm long, 0.73–0.80 cm and an acute angle between the horns. ***Ovulate strobili*** 15–26 cm long, 7.5–9.5 cm in diameter, solitary, cylindrical, erect, green with reddish brown trichomes at emergence, green with brown to blackish trichomes at maturity, acute apex; peduncle 5–18.5 cm long, 1.6–2 cm in diameter, pendulous and erect, tomentose, light brown; megasporophylls 30–56, 6–8 orthostichies with 5–7 sporophylls per orthostichy, 1.5–2.0 × 3.5–4.0 cm, with a prominent distal face, horns curved to straight and 0.70–0.90 cm long, 0.92–1.56 cm between horns with a right angle between the horns. ***Seeds*** 2–2.8 cm long, 1.8–2 cm in diameter, ovate, sarcotesta whitish pink at emergence, light brown at maturity.

##### Distribution and habitat.

*Ceratozamiazoquorum* is endemic to the northern mountains of Chiapas, Mexico (Fig. 29D), where it occurs on karstic outcrops in evergreen tropical forest and oak forest between 500 and 1,150 m.

##### Etymology.

The specific epithet was established in honor of the Zoque culture (Pérez-Farrera et al. 2001).

##### Common names.

None recorded.

##### Uses.

None recorded.

##### Conservation status.

(IUCN 2021). *Ceratozamiazoquorum* is listed as “Critically Endangered” under criteria A2c+4c; B1ab(i,ii,iii,v).

##### Discussion.

*Ceratozamiazoquorum* has oblong and coriaceous leaflets and leaves with scarce thin and short prickles. It belongs to a cryptic taxonomic group, and is geographically close to populations of *C.becerrae* and *C.santillanii*, the other two species in this group. The three taxa are distinguishable with the nrITS (Martínez-Domínguez et al. 2017c). Morphologically, *C.zoquorum* differs from *C.santillanii* by its peduncle of ovulate strobili more than 3 cm long.

##### Specimens examined.

Mexico. **Chiapas: Mun. Solosuchiapa**, 530 m, 23 Jan 2014, *F. Nicolalde-Morejón et al. 1931, 1932* (CIB); 550 m, 23 Jan 2014, *F. Nicolalde-Morejón et al. 1933–1935* (CIB); 682 m, 24 Jan 2014, *F. Nicolalde-Morejón et al. 1936–1947* (CIB); 531 m, 23 Jan 2014, *L. Martínez-Domínguez et al. 1–5* (CIB); 500 m, 23 Jan 2014, *L. Martínez-Domínguez et al. 6–14* (CIB); 682 m, 23 Jan 2014, *L. Martínez-Domínguez et al. 15–34* (CIB); 520 m, 17 Apr 1996, *M.A. Pérez-Farrera 905* (CH, CHIP, HEM); 520 m, 16 Apr 1996, *M.A. Pérez-Farrera s/n* (HEM); 19 Jan 2001, *S. Avendaño 5216* (MEXU); 531 m, 20 Jan 2001, *T.W. Walters 2001–2028-A* (XAL). **Mun. Tila**, 1,135 m, 16 Jul 2021, *F. Nicolalde-Morejón et al. 3698–3702* (CIB); 1,135 m, 16 Jul 2021, *L. Martínez-Domínguez et al. 2326–2330* (CIB).

### ﻿“Names” (designations) not validly published

*Ceratozamiaangustifolia* Linden, Illustr. Hort. 28: 32. 1881, nomen nudum, name in list, no description and diagnosis.

*Ceratozamiaensiformis* hort. ex J.Schust., Pflanzenr. 99: 130. 1932, pro syn.

*Ceratozamiaeriolepis* hort. ex J.Schust., Pflanzenr. 99: 132. 1932, pro syn.

*Ceratozamiafusca* hort. ex J.Schust., Pflanzenr. 99: 132. 1932, pro syn.

*Ceratozamiafuscata* hort. ex J.Schust., Pflanzenr. 99: 132.1932, pro syn.

*Ceratozamiaghiesbreghtii* Brongn., Compte Rendue 81: 303. 1875, nomen nudum, name in list, no description and diagnosis.

*Ceratozamia×hybrida* J.Schust., Pflanzenr. 99: 132. 1932, pro syn.

*Ceratozamiakarsteniana* hort. ex Dyer, Biol. Cent.-Amer., Bot. 3: 192. 1884, pro syn. Thiselton-Dyer cited this name as synonym of *C.latifolia*.

*Ceratozamialongipinnata* hort. ex J.Schust., Pflanzenr. 99: 130. 1932, pro syn.

*Ceratozamiamiquelii* hort., Vilm. Blumengärtn., ed. 3. 1: 1246. 1895, pro syn.

*Ceratozamiamuricata* Miq. ex Linden, Illustr. Hort. 32. 1881, nomen nudum.

*Ceratozamiaottonis* hort. ex J.Schust., Pflanzenr. 99: 130. pro syn. 1932, pro syn.

*Ceratozamiapurpurea* Matte, Recherches Appareil Libéro-Lign. Cycad. 125. 1914, nomen nudum.

*Dipsacozamia* Lehm. ex Lindl., The Vegetable Kingdom, 225. 1846, nomen nudum, not validly published; no diagnosis and description (Art. 39).

*Dipsacozamiamexicana* Liebm. ex Dyer, Biol. Cent.-Amer., Bot. 3: 193. 1884, pro syn. Thiselton-Dyer cited this name as synonym of *C.mexicana*.

### ﻿Excluded names

*Ceratozamiaboliviana* Brongn., Ann. Sci. Nat., Bot. ser. 3, 5: 9. 1846. Lectotype: P [P02441739]. Taxonomic Status: synonym of *Zamiaboliviana* (Brongn.) A.DC.

*Ceratozamiakatzeriana* Regel, Trudy Imp. S.-Peterburgsk. Bot. Sada 4(4): 298. 1876. Lectotype: LE [LE00009045]. Taxonomic Status: synonym of *Zamiakatzeriana* (Regel) E.Rettig.

## Supplementary Material

XML Treatment for Ceratozamia

XML Treatment for Ceratozamia alvarezii

XML Treatment for Ceratozamia aurantiaca

XML Treatment for Ceratozamia becerrae

XML Treatment for Ceratozamia brevifrons

XML Treatment for Ceratozamia chamberlainii

XML Treatment for Ceratozamia chimalapensis

XML Treatment for Ceratozamia decumbens

XML Treatment for Ceratozamia delucana

XML Treatment for Ceratozamia euryphyllidia

XML Treatment for Ceratozamia fuscoviridis

XML Treatment for Ceratozamia hildae

XML Treatment for Ceratozamia hondurensis

XML Treatment for Ceratozamia huastecorum

XML Treatment for Ceratozamia kuesteriana

XML Treatment for Ceratozamia latifolia

XML Treatment for Ceratozamia leptoceras

XML Treatment for Ceratozamia matudae

XML Treatment for Ceratozamia mexicana

XML Treatment for Ceratozamia miqueliana

XML Treatment for Ceratozamia mirandae

XML Treatment for Ceratozamia mixeorum

XML Treatment for Ceratozamia morettii

XML Treatment for Ceratozamia norstogii

XML Treatment for Ceratozamia oliversacksii

XML Treatment for Ceratozamia osbornei

XML Treatment for Ceratozamia robusta

XML Treatment for Ceratozamia sabatoi

XML Treatment for Ceratozamia sancheziae

XML Treatment for Ceratozamia santillanii

XML Treatment for Ceratozamia subroseophylla

XML Treatment for Ceratozamia tenuis

XML Treatment for Ceratozamia totonacorum

XML Treatment for Ceratozamia vovidesii

XML Treatment for Ceratozamia whitelockiana

XML Treatment for Ceratozamia zaragozae

XML Treatment for Ceratozamia zoquorum

## Supplementary materials

Supplementary material 1 Glossary Data type Text Explanation note Glossary for characters associated to reproductive structures. https://binary.pensoft.net/file/746090This dataset is made available under the Open Database License (http://opendatacommons.org/licenses/odbl/1.0/). The Open Database License (ODbL) is a license agreement intended to allow users to freely share, modify, and use this Dataset while maintaining this same freedom for others, provided that the original source and author(s) are credited.L. Martínez-Domínguez, F. Nicolalde-Morejón, D. W. Stevenson

Supplementary material 2 Herbarium specimens for *Ceratozamiasancheziae* and *C.zoquorum* Data type Images Explanation note Specimens: M.A. Pérez-Farrera 1635; D.E. Breedlove 25506. https://binary.pensoft.net/file/746091This dataset is made available under the Open Database License (http://opendatacommons.org/licenses/odbl/1.0/). The Open Database License (ODbL) is a license agreement intended to allow users to freely share, modify, and use this Dataset while maintaining this same freedom for others, provided that the original source and author(s) are credited.Author: L. Martínez-Domínguez
