# Supplementary material for: ﻿Monograph of Ceratozamia (Zamiaceae, Cycadales): an endangered genus
Source: PhytoKeys. 2022 Sep 21;208:1–102. doi: 10.3897/phytokeys.208.80382 (PMC9849018; doi:10.3897/phytokeys.208.80382)
Supplement: Supplementary material 1 — Glossary [file phytokeys-208-001_article-80382__-s001.docx]

**Supplementary material 1.** Glossary for characters associated with reproductive structures. The character states are defined in relation to the structure and position.

Acute: Area between the horns of a microsporophyll or megasporophyll forms an angle < 90º.

Curved: Horns of the microsporophylls are curved. This term also is used for horns of the megasporophylls.

Deeply lobate: Converging point between infertile and fertile portion of the microsporophyll forms an extended lobe.

Discoid: Microsporophyll shape rounded like a disk.

Elliptic: Microsporophylls similar to a narrow oval or ellipse.

Erect: The orientation of peduncle of ovulate and pollen strobili not curved.

Globose: Seed circular with regular outline.

Linear: The distal end of the microsporophylls have an almost straight outline.

Lobate: Converging point between infertile and fertile portion of the microsporophyll forms a lobe.

Non-recurved: Distal end of the microsporophylls straight. This term also is used for horns of the megasporophylls.

Obconic: Microsporophyll gradually reduced from the distal end towards the cone axis.

Obtuse: Area between the horns of a microsporophyll or megasporophyll forms an angle >90º.

Orbicular: The distal end of the microsporophylls is circular.

Ovate: Shape egg shaped, i.e., broad funiculus attachment.

Pendulous: The orientation of the peduncle of an ovulate or pollen strobilus droops downward.

Prominent: Distal face of the megasporophylls is thick.

Recurved: Distal end of the microsporophylls is curved downward (abaxially). This term also is used for horns of the megasporophylls.

Right: Area between the horns of a microsporophyll or megasporophyll forms angle of 90º.

Robust horns: horns are thick and strong.

Rounded: The distal end of the microsporophylls have an outline slightly curved in proximal to fertile portion.

Spherical: Seed shape a sphere that may also be may be irregular.

Straight: Horns of the microsporophyll are not curved.

Thin horns: horns are narrow and break easily.

Truncate: Distal face of the megasporophylls almost planar or slightly concave.
