# Supplementary material for: ﻿Monograph of Ceratozamia (Zamiaceae, Cycadales): an endangered genus
Source: PhytoKeys. 2022 Sep 21;208:1–102. doi: 10.3897/phytokeys.208.80382 (PMC9849018; doi:10.3897/phytokeys.208.80382)

C. zoquorum

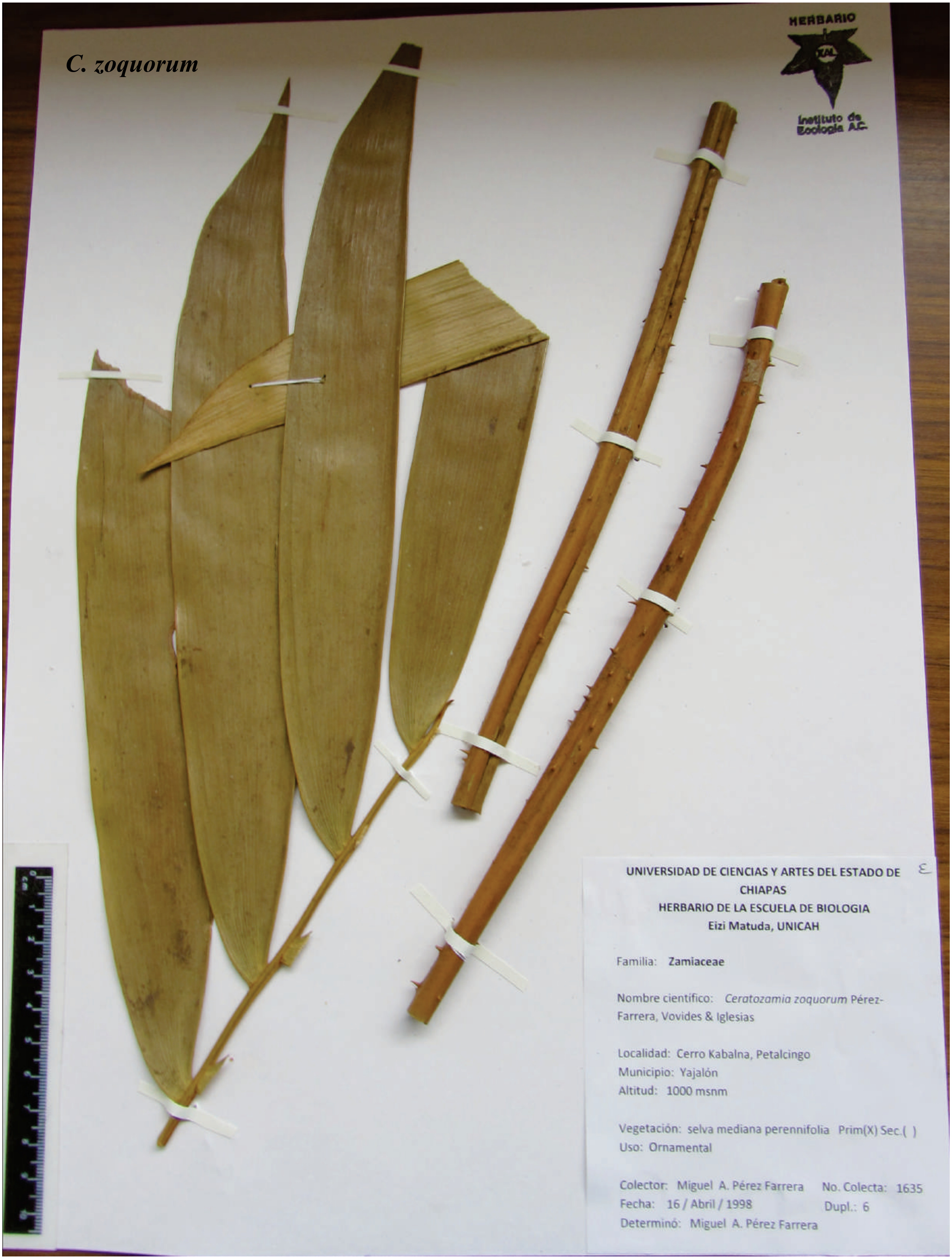
HERBARIO
XAL
Instituto de Ecología A.C.
UNIVERSIDAD DE CIENCIAS Y ARTES DEL ESTADO DE CHIAPAS
HERBARIO DE LA ESCUELA DE BIOLOGIA
Eizi Matuda, UNICAH
Familia: Zamiaceae
Nombre científico: Ceratozamia zoquorum Pérez-Farrera, Vovides & Iglesias
Localidad: Cerro Kabalna, Petalcingo
Municipio: Yajalón
Altitud: 1000 msnm
Vegetación: selva mediana perennifolia Prim(X) Sec.( )
Uso: Ornamental
Colector: Miguel A. Pérez Farrera
No. Colecta: 1635
Fecha: 16 / Abril / 1998
Dupl.: 6
Determinó: Miguel A. Pérez Farrera

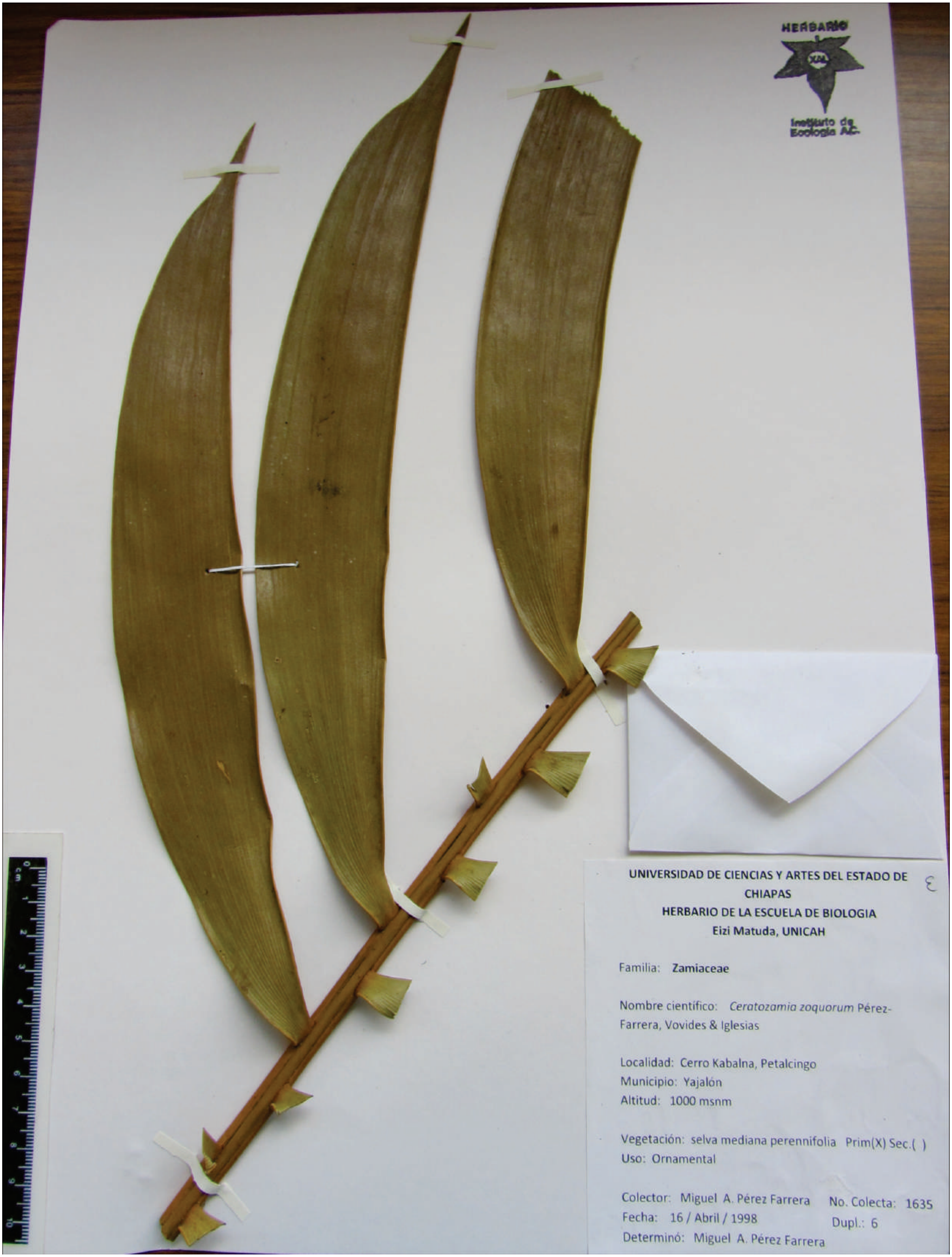
HERBARIO
XAL
Instituto de Ecología A.C.
UNIVERSIDAD DE CIENCIAS Y ARTES DEL ESTADO DE CHIAPAS
HERBARIO DE LA ESCUELA DE BIOLOGIA
Eizi Matuda, UNICAH
Familia: Zamiaceae
Nombre científico: Ceratozamia zoquorum Pérez-Farrera, Vovides & Iglesias
Localidad: Cerro Kabalna, Petalcingo
Municipio: Yajalón
Altitud: 1000 msnm
Vegetación: selva mediana perennifolia Prim(X) Sec.( )
Uso: Ornamental
Colector: Miguel A. Pérez Farrera
No. Colecta: 1635
Fecha: 16 / Abril / 1998
Dupl.: 6
Determinó: Miguel A. Pérez Farrera

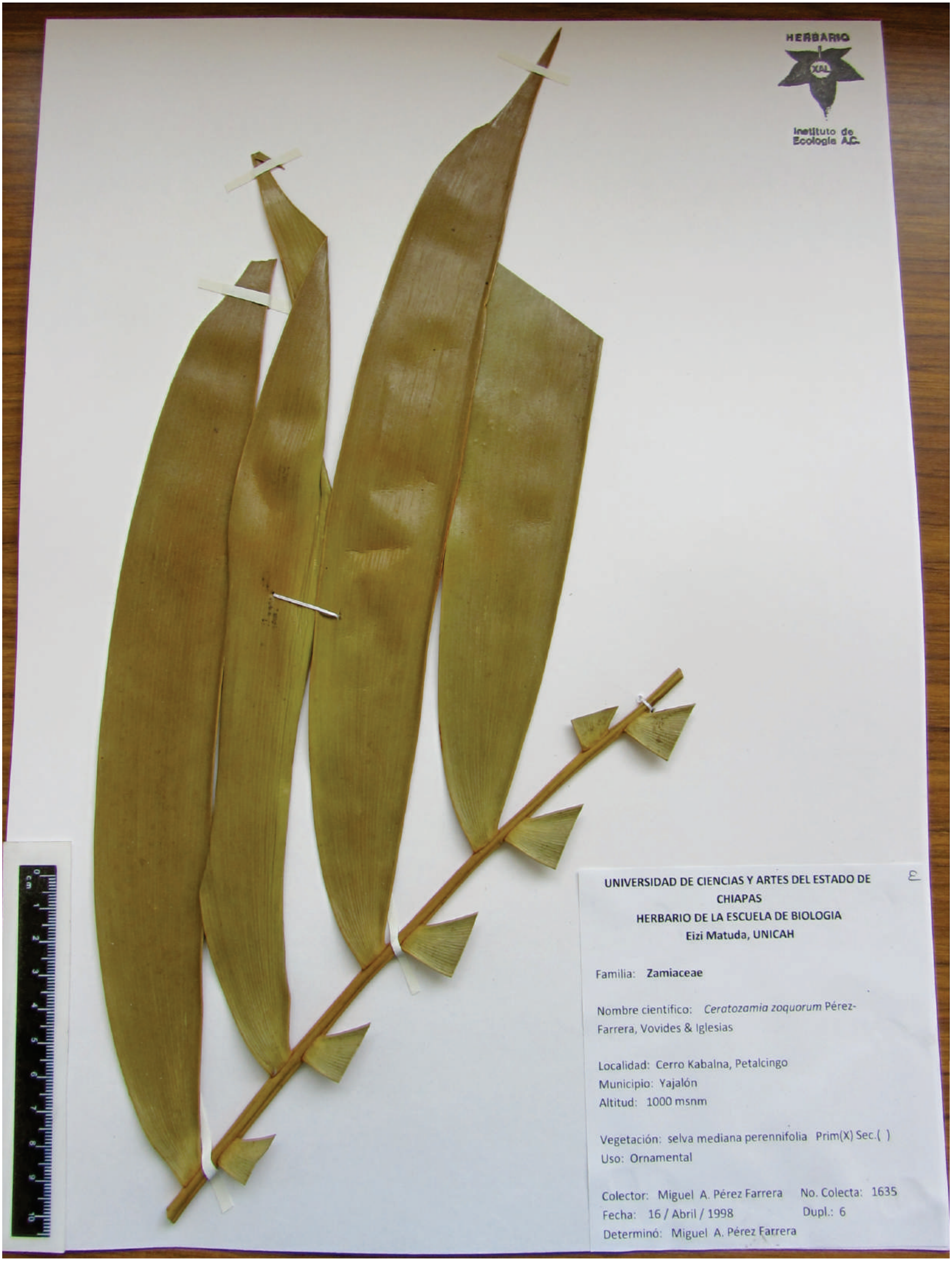
HERBARIO
XAL
Instituto de Ecología A.C.
UNIVERSIDAD DE CIENCIAS Y ARTES DEL ESTADO DE CHIAPAS
HERBARIO DE LA ESCUELA DE BIOLOGIA
Eizi Matuda, UNICAH
Familia: Zamiaceae
Nombre cientifico: Ceratozamia zoquorum Pérez-Farrera, Vovides & Iglesias
Localidad: Cerro Kabalna, Petalcingo
Municipio: Yajalón
Altitud: 1000 msnm
Vegetación: selva mediana perennifolia Prim(X) Sec.( )
Uso: Ornamental
Colector: Miguel A. Pérez Farrera
No. Colecta: 1635
Fecha: 16 / Abril / 1998
Dupl.: 6
Determinó: Miguel A. Pérez Farrera

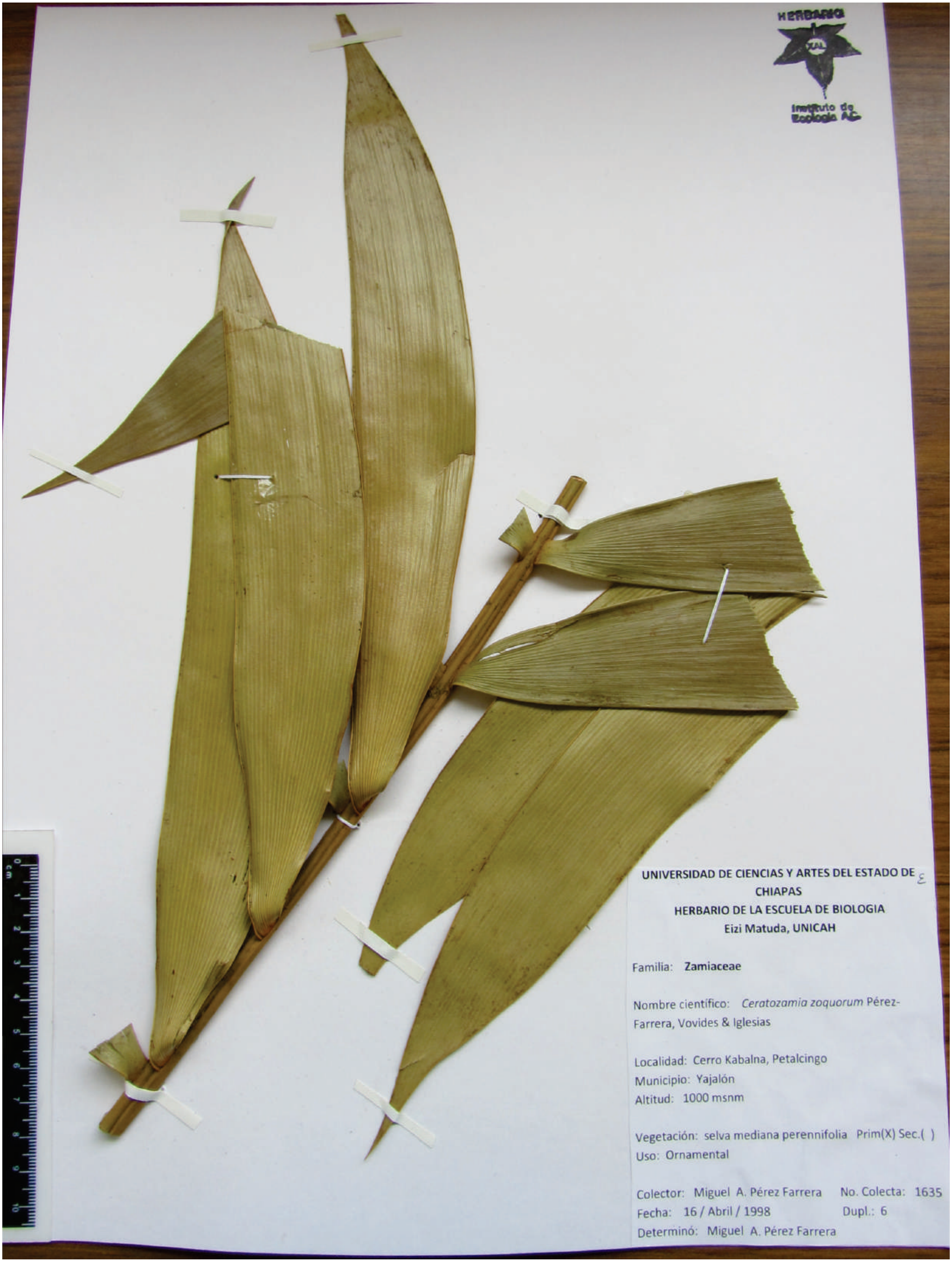
HERBARIO
XAL
Instituto de Ecología A.C.
UNIVERSIDAD DE CIENCIAS Y ARTES DEL ESTADO DE CHIAPAS
HERBARIO DE LA ESCUELA DE BIOLOGIA
Eizi Matuda, UNICAH
Familia: Zamiaceae
Nombre científico: Ceratozamia zoquorum Pérez-Farrera, Vovides & Iglesias
Localidad: Cerro Kabalna, Petalcingo
Municipio: Yajalón
Altitud: 1000 msnm
Vegetación: selva mediana perennifolia Prim(X) Sec.( )
Uso: Ornamental
Colector: Miguel A. Pérez Farrera No. Colecta: 1635
Fecha: 16 / Abril / 1998 Dupl.: 6
Determinó: Miguel A. Pérez Farrera

C. sancheziae - Paratype

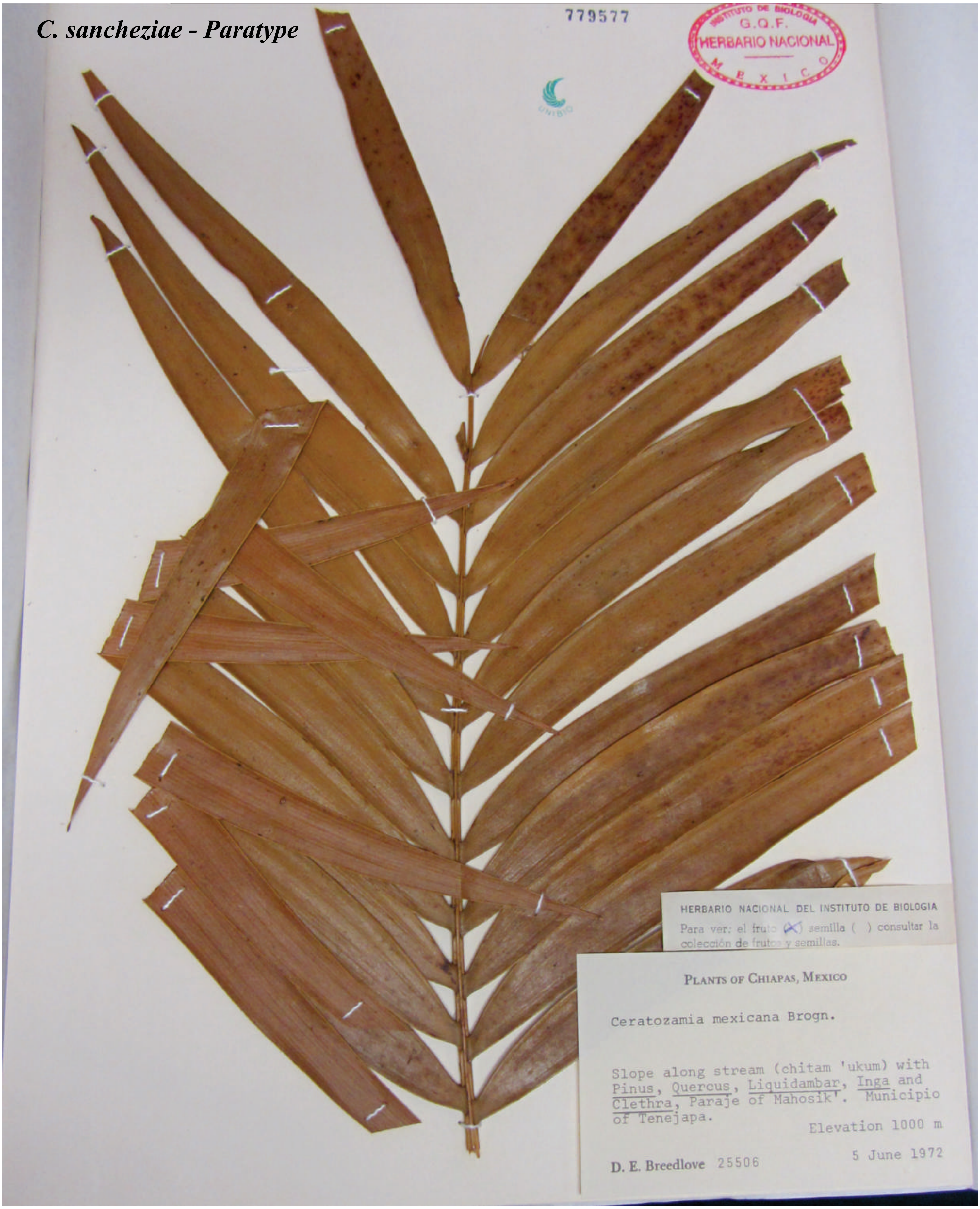

Supplement: Supplementary material 2 — Herbarium specimens for Ceratozamiasancheziae and C.zoquorum [file phytokeys-208-001_article-80382__-s002.pdf]
